# Supplementary material for: Unusual Tetrahydropyridoindole-Containing Tetrapeptides with Human Nicotinic Acetylcholine Receptors Targeting Activity Discovered from Antarctica-Derived Psychrophilic Pseudogymnoascus sp. HDN17-933
Source: Mar Drugs. 2022 Sep 22;20(10):593. doi: 10.3390/md20100593 (PMC9604729; doi:10.3390/md20100593)
Supplement: Supplementary file 1 [file marinedrugs-20-00593-s001.zip › marinedrugs-1909373-supplementary.pdf]

SUPPLEMENTARY MATERIAL

Unusual Tetrahydropyridoindole-Containing Tetrapeptides with Human Nicotinic Acetylcholine Receptors targeting Activity Discovered from Antarctica-Derived Psychrophilic Pseudogymnoascus sp. HDN17-933

Xuewen Hou [1], Changlong Li [1], Runfang Zhang [1], Yinping Li [1], Huadong Li [1], Yundong Zhang [1], HanShen Tae [2], Rilei Yu [1], Qian Che [1], Tianjiao Zhu [1], Dehai Li [1,3,4,*] ,and Guojian Zhang [1,3,5,*]

[1] Key Laboratory of Marine Drugs, Chinese Ministry of Education, School of Medicine and Pharmacy, Ocean University of China, Qingdao 266003, China

[2] Illawarra Health and Medical Research Institute (IHMRI), University of Wollongong, Wollongong, New South Wales 2522, Australia

[3] Laboratory for Marine Drugs and Bioproducts, Pilot National Laboratory for Marine Science and Technology, Qingdao 266237, China

[4] Open Studio for Druggability Research of Marine Natural Products, Pilot National Laboratory for Marine Science and Technology, Qingdao 266237, China

[5] Marine Biomedical Research Institute of Qingdao, Qingdao 266101, China

[*] Correspondence: zhangguojian@ouc.edu.cn (G.Z.); Tel: +86-532-82032971

dehaili@ouc.edu.cn (D.L.); Tel.: +86-532-8203-1619

ABSTRACT

Chemical investigation of the psychrophilic fungus *Pseudogymnoascus* sp. HDN17-933 derived from Antarctica led to the discovery of six new tetrapeptides psegynamides A-F (**1-6**), whose planar structures were elucidated by extensive NMR and MS spectrometric analyses. Structurally, psegynamides D-F (**4-6**) possess unique backbones bearing a tetrahydropyridoindoles unit, which make them the first examples discovered in naturally occurring peptides. The absolute configurations of structures were unambiguously determined using solid-phase total synthesis assisted by Marfey's method, and all compounds were evaluated for their inhibition of human (h) nicotinic acetylcholine receptor subtypes. Compound **2** showed significant inhibitory activity. Preliminary structure-activity relationship investigation revealed that the tryptophan residue and the C-terminal with methoxy group were important to the inhibitory activity. Further, the high binding affinity of compound **2** to h$\alpha 4\beta 2$ was explained by by molecular docking studies.

Table of Contents

Figure S1. 18S rRNA sequences data of *Pseudogymnoascus* sp. HDN17-933

Figure S2. HPLC analysis of the crude extract of HDN17-933.

Figure S3. ^{1}H-NMR spectrum (600 MHz, DMSO-d_6) of compound **1**

Figure S4. ^{13}C-NMR spectrum (150 MHz, DMSO-d_6) of compound **1**

Figure S5. DEPT (150 MHz, DMSO-d_6) spectrum of compound **1.**

Figure S6. HSQC spectrum (600 MHz, DMSO-d_6) of compound **1**

Figure S7. ^{1}H-^{1}H COSY spectrum (600 MHz, DMSO-d_6) of compound **1**

Figure S8. HMBC spectrum (600 MHz, DMSO-d_6) of compound **1**

Figure S9. NOESY spectrum (600 MHz, DMSO-d_6) of compound **1**

Figure S10. TOCSY spectrum (600 MHz, DMSO-d_6) of compound **1**

Figure S11. HRESIMS spectrum of compound **1**.

Figure S12. IR spectrum of compound **1.**

Figure S13. ^{1}H-NMR spectrum (600 MHz, DMSO-d_6) of compound **2**

Figure S14. ^{13}C-NMR spectrum (150 MHz, DMSO-d_6) of compound **2**

Figure S15. DEPT (150 MHz, DMSO-d_6) spectrum of compound **2**

Figure S16. HSQC spectrum (600 MHz, DMSO-d_6) of compound **2**

Figure S17. ^{1}H-^{1}H COSY spectrum (600 MHz, DMSO-d_6) of compound **2**

Figure S18. HMBC spectrum (600 MHz, DMSO-d_6) of compound **2**

Figure S19. TOCSY spectrum (600 MHz, DMSO-d_6) of compound **2**

Figure S20. HRESIMS spectrum of compound **2**

Figure S21. IR spectrum of compound **2**

Figure S22. ^{1}H-NMR spectrum (600 MHz, DMSO-d_6) of compound **3**

Figure S23. ^{13}C-NMR spectrum (150 MHz, DMSO-d_6) of compound **3**

Figure S24. DEPT (150 MHz, DMSO-d_6) spectrum of compound **3**

Figure S25. HSQC spectrum (600 MHz, DMSO-d_6) of compound **3**

Figure S26. ^{1}H-^{1}H COSY spectrum (600 MHz, DMSO-d_6) of compound **3**

Figure S27. HMBC spectrum (600 MHz, DMSO-d_6) of compound **3**

Figure S28. TOCSY spectrum (600 MHz, DMSO-d_6) of compound **3**

Figure S29. HRESIMS spectrum of compound **3**

Figure S30. IR spectrum of compound **3**

Figure S31. ^{1}H-NMR spectrum (600 MHz, DMSO-d_6) of compound **4**

Figure S32. ^{13}C-NMR spectrum (150 MHz, DMSO-d_6) of compound **4**

Figure S33. DEPT (150 MHz, DMSO-d_6) spectrum of compound **4**

Figure S34. HSQC spectrum (600 MHz, DMSO-d_6) of compound **4**

Figure S35. HMBC spectrum (600 MHz, DMSO-d_6) of compound **4**

Figure S36. NOESY spectrum (600 MHz, DMSO-d_6) of compound **4**

Figure S37. TOCSY spectrum (600 MHz, DMSO-d_6) of compound **4**

Figure S38. HRESIMS spectrum of compound **4**

Figure S39. IR spectrum of compound **4**

Figure S40. ^{1}H-NMR spectrum (600 MHz, DMSO-d_6) of compound **5**

Figure S41. ^{13}C-NMR spectrum (150 MHz, DMSO-d_6) of compound **5**

Figure S42. DEPT (150 MHz, DMSO-d_6) spectrum of compound **5**

Figure S43. HSQC spectrum (600 MHz, DMSO-d_6) of compound **5**

Figure S44. ^{1}H-^{1}H COSY spectrum (600 MHz, DMSO-d_6) of compound **5**

Figure S45. HMBC spectrum (600 MHz, DMSO-d_6) of compound **5**

Figure S46. TOCSY spectrum (600 MHz, DMSO-d_6) of compound **5**

Figure S47. HRESIMS spectrum of compound **5**

Figure S48. IR spectrum of compound **5**

Figure S49. ^{1}H-NMR spectrum (600 MHz, DMSO-d_6) of compound **6**

Figure S50. ^{13}C-NMR spectrum (150 MHz, DMSO-d_6) of compound **6**

Figure S51. DEPT (150 MHz, DMSO-d_6) spectrum of compound **6**

Figure S52. HSQC spectrum (600 MHz, DMSO-d_6) of compound **6**

Figure S53. ^{1}H-^{1}H COSY spectrum (600 MHz, DMSO-d_6) of compound **6**

Figure S54. HMBC spectrum (600 MHz, DMSO-d_6) of compound **6**

Figure S55. TOCSY spectrum (600 MHz, DMSO-d_6) of compound **6**

Figure S56. HRESIMS spectrum of compound **6**

Figure S57. IR spectrum of compound **6**

Figure S58. Advanced Marfey's acid hydrolytic analysis of compound **1**

Figure S59. Advanced Marfey's alkaline hydrolytic analysis of compound **1**

Figure S60. ^{1}H-NMR spectrum of **1a** (400 MHz, DMSO-d_6)

Figure S61. ^{13}C-NMR spectrum of **1a** (100 MHz, DMSO-d_6)

Figure S62. ^{1}H-NMR spectrum of **1b** (400 MHz, DMSO-d_6)

Figure S63. ^{13}C-NMR spectrum of **1b** (100 MHz, DMSO-d_6)

Figure S64. Comparison of ^{1}H-NMR spectra of authentic **1, 1a,** and **1b**

Figure S65. Advanced Marfey's acid hydrolytic analysis of compound **2**

Figure S66. Advanced Marfey's alkaline hydrolytic analysis of compound **2**

Figure S67. ^{1}H-NMR spectrum of **2a** (400 MHz, DMSO-d_6)

Figure S68. ^{13}C-NMR spectrum of **2a** (100 MHz, DMSO-d_6)

Figure S69. ^{1}H-NMR spectrum of **2b** (400 MHz, DMSO-d_6)

Figure S70. ^{13}C-NMR spectrum of **2b** (100 MHz, DMSO-d_6)

Figure S71. Comparison of ^{1}H-NMR spectra of authentic **2, 2a,** and 2**b**

Figure S72. Advanced Marfey's acid hydrolytic analysis of compound **3**

Figure S73. Advanced Marfey's alkaline hydrolytic analysis of compound **3**

Figure S74. ^{1}H-NMR spectrum of synthetic **3** (400 MHz, DMSO-d_6)

Figure S75. ^{13}C-NMR spectrum of synthetic **3** (100 MHz, DMSO-d_6)

Figure S76. Comparison of ^{1}H-NMR spectra of **3** with synthetic **3**

Figure S77. Advanced Marfey's acid hydrolytic analysis of compound **4**

Figure S78. ^{1}H-NMR spectrum of synthetic **4** (400 MHz, DMSO-d_6)

Figure S79. ^{13}C-NMR spectrum of synthetic **4** (100 MHz, DMSO-d_6)

Figure S80. Comparison of ^{1}H-NMR spectra of **4,** with synthestic **4**

Figure S81. Advanced Marfey's acid hydrolytic analysis of compound **5**

Figure S82. ^{1}H-NMR spectrum of synthetic **5** (400 MHz, DMSO-d_6)

Figure S83. ^{13}C-NMR spectrum of synthetic **5** (100 MHz, DMSO-d_6)

Figure S84. Comparison of ^{1}H-NMR spectra of **5** with synthetic **5**

Figure S85. Advanced Marfey's acid hydrolytic analysis of compound **6**

Figure S86. ^{1}H-NMR spectrum of synthetic **6** (400 MHz, DMSO-d_6)

Figure S87. ^{13}C-NMR spectrum of synthetic **6** (100 MHz, DMSO-d_6)

Figure S88. Comparison of ^{1}H-NMR spectra of **6** with synthetic **6**

Table S1. Table of compounds (**1-6**) (100 μM) inhibition of ACh-evoked peak current amplitude mediated by human (h) $\alpha1\beta1\varepsilon\delta$, $\alpha1\beta1\gamma\delta$, $\alpha3\beta2$, $\alpha3\beta4$, $\alpha4\beta2$, $\alpha7$ and $\alpha9\alpha10$ nAChRs.

Table S2. Free binding energy estimation and molecular interactions of compounds **1-6**

GGGCAACGTGTCGACGGGTTGCCGCAGGCCTCCCGGGTAACCT
ACCACCCTTTGTTTATTATACTTTGTTGCTTTGGCAGGCCTGCCC
TCGGGCTGCTGGCTCCGGCCGGCGAGCGCTTGCCAGAGGACCT
AAACTCTGTTTGTCTATACTGTCTGAGTACTATATAATAGTTAAA
ACTTTCAACAACGGATCTCTTGGTTCTGGCATCGATGAAGAACG
CAGCGAAATGCGATAAGTAATGTGAATTGCAGAATTCAGTGAAT
CATCGAATCTTTGAACGCACATTGCGCCCCCTGGTATTCCGGGG
GGCATGCCTGTCCGAGCGTCATTACAACCCTCAAGCTCAGCTTG
GTATTGGGCCCCGCCGACCCGGCGGGCCCTAAAGTCAGTGGCG
GTGCCGTCCGGCTCCGAGCGTAGTAATTCTTCTCGCTCTGGAGG
CCCGGTCGTGTGCTCGCCAGCAACCCCCAATTTTTTTCAGGTTG
ACCTCGGATCAGGTAGGGATACCCGCTGAACTTAAGCATATCAA
TAAGCGGAGGAAATTACAG

Figure S1. 18S rRNA sequences data of *Pseudogymnoascus* sp. HDN17-933

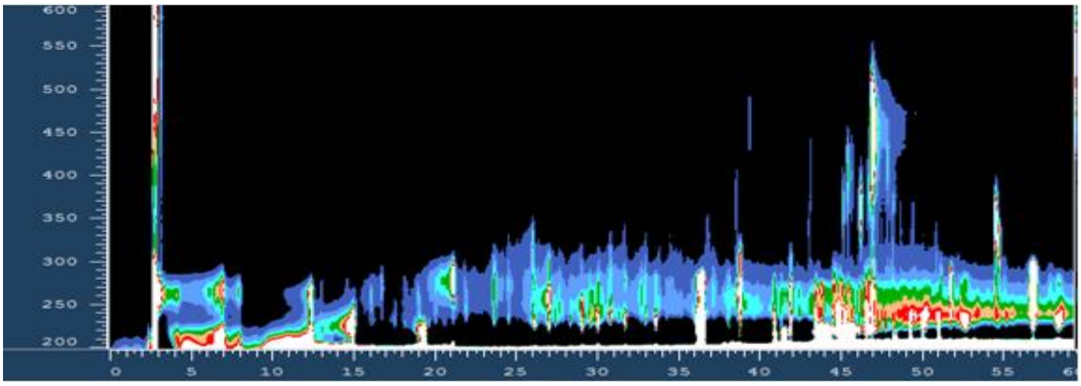

Figure S2. HPLC analysis of the crude extract of HDN17-933.

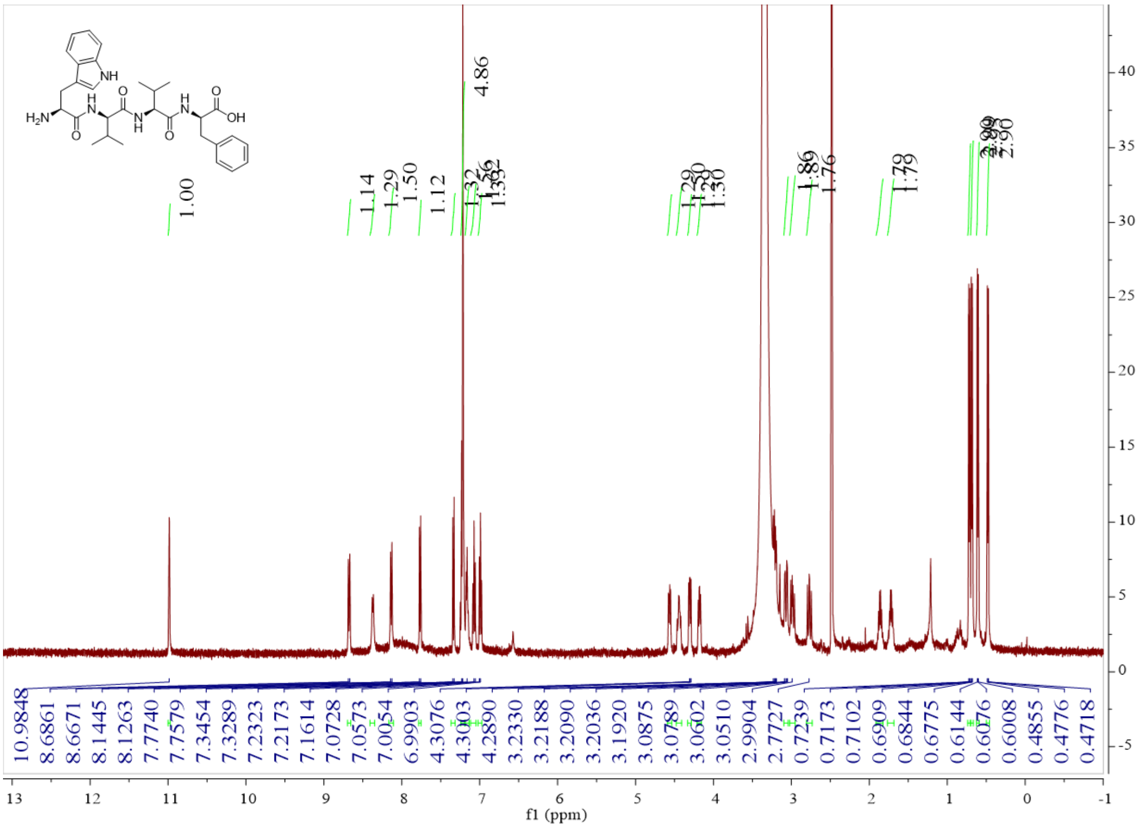

Figure S3. ^{1}H-NMR spectrum (600 MHz, DMSO-d_6) of compound **1**

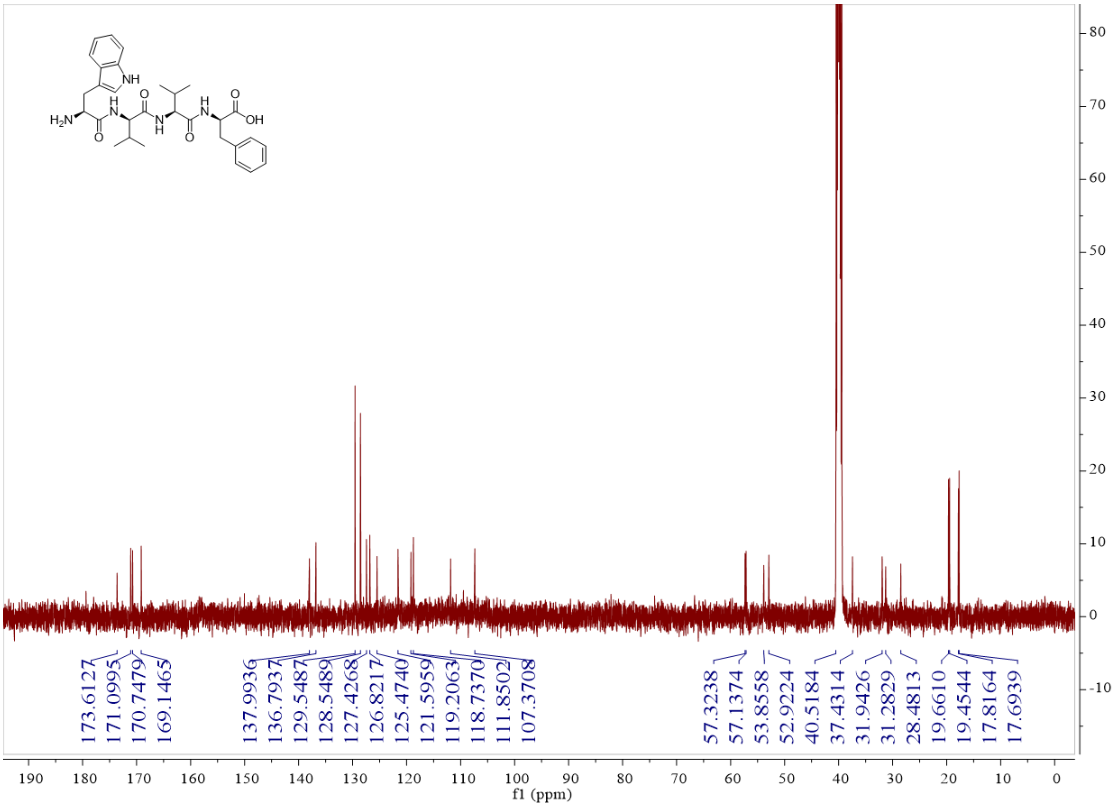

Figure S4. ^{13}C-NMR spectrum (150 MHz, DMSO-d_6) of compound **1**

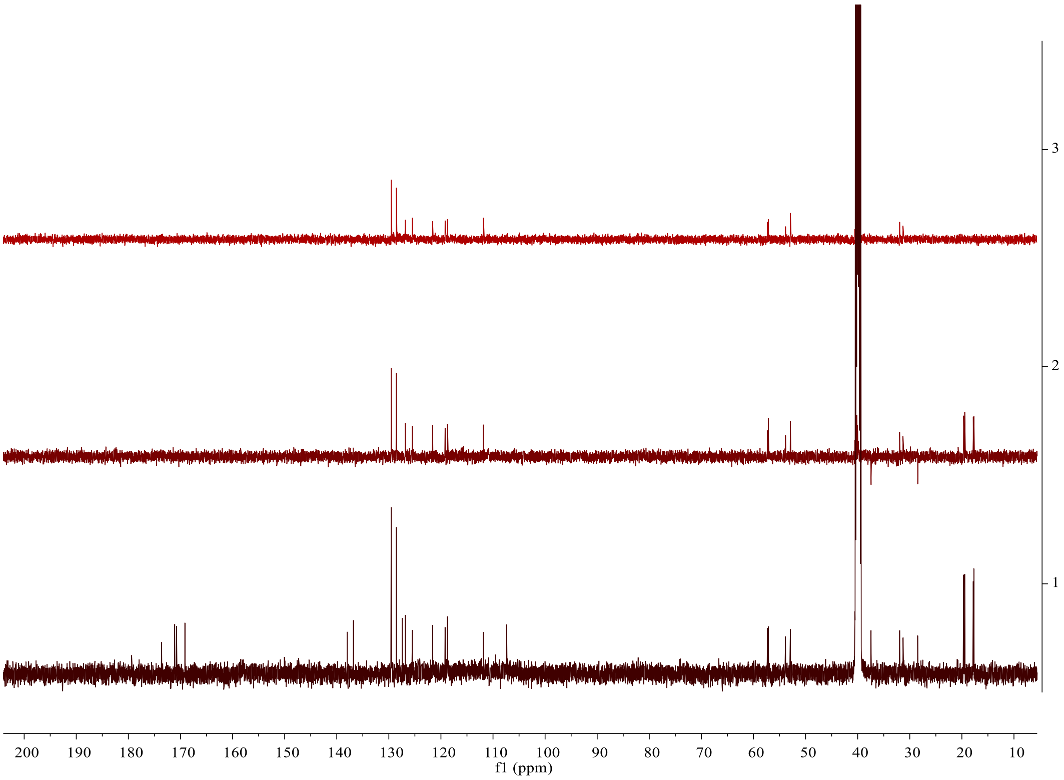

Figure S5. DEPT (150 MHz, DMSO-d_6) spectrum of compound **1.**

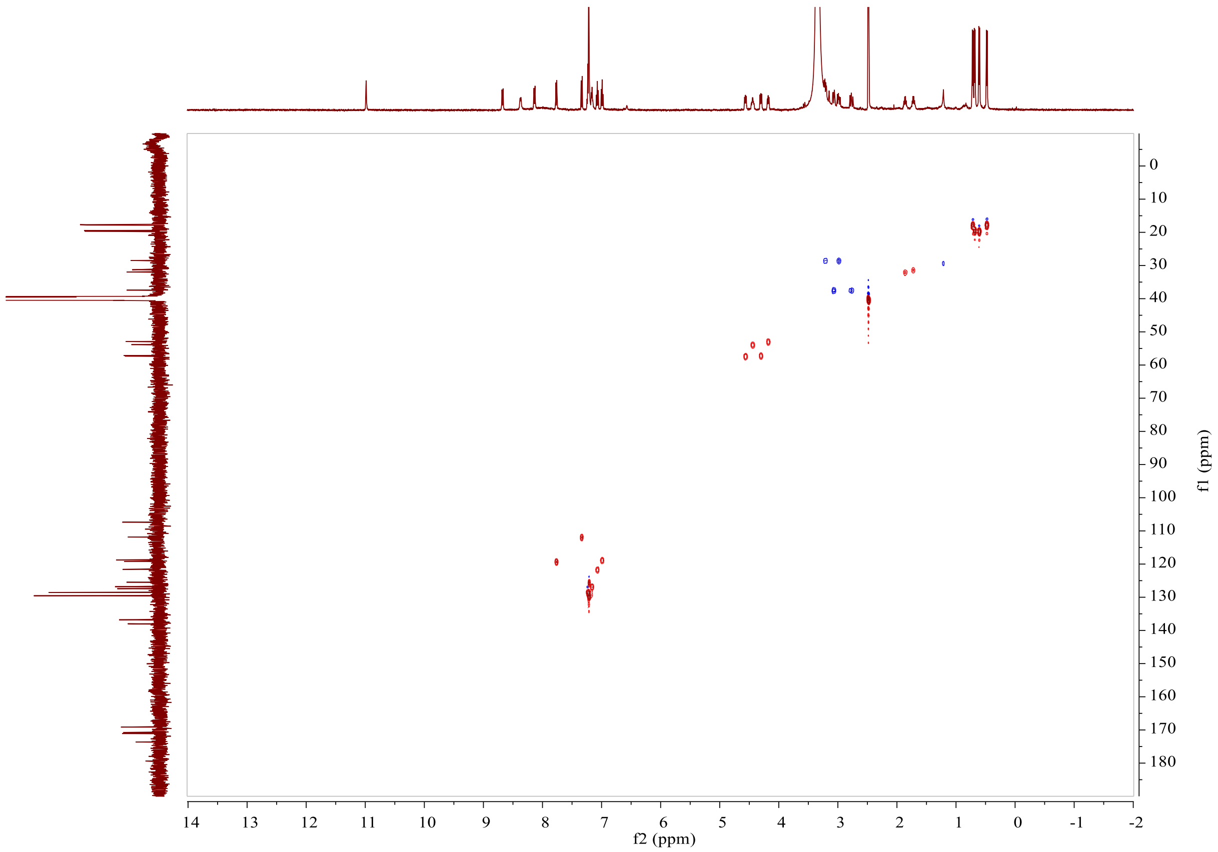

Figure S6. HSQC spectrum (600 MHz, DMSO-d_6) of compound **1.**

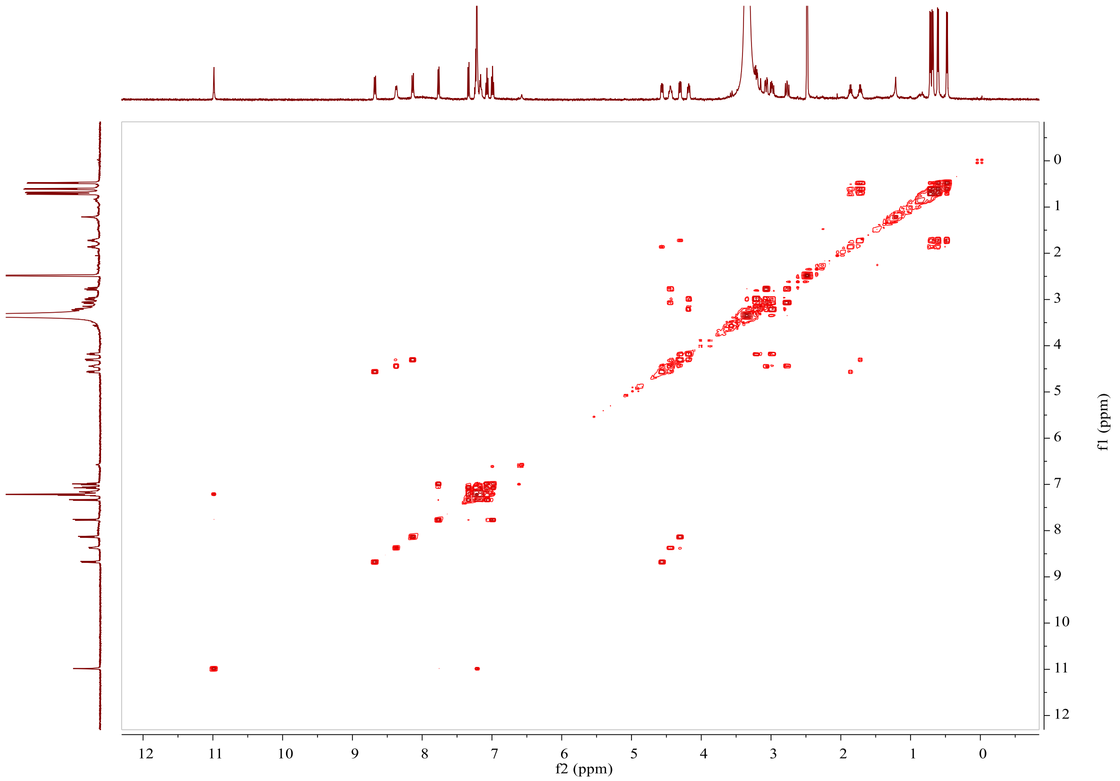

Figure S7. ^{1}H-^{1}H COSY spectrum (600 MHz, DMSO-d_6) of compound **1**

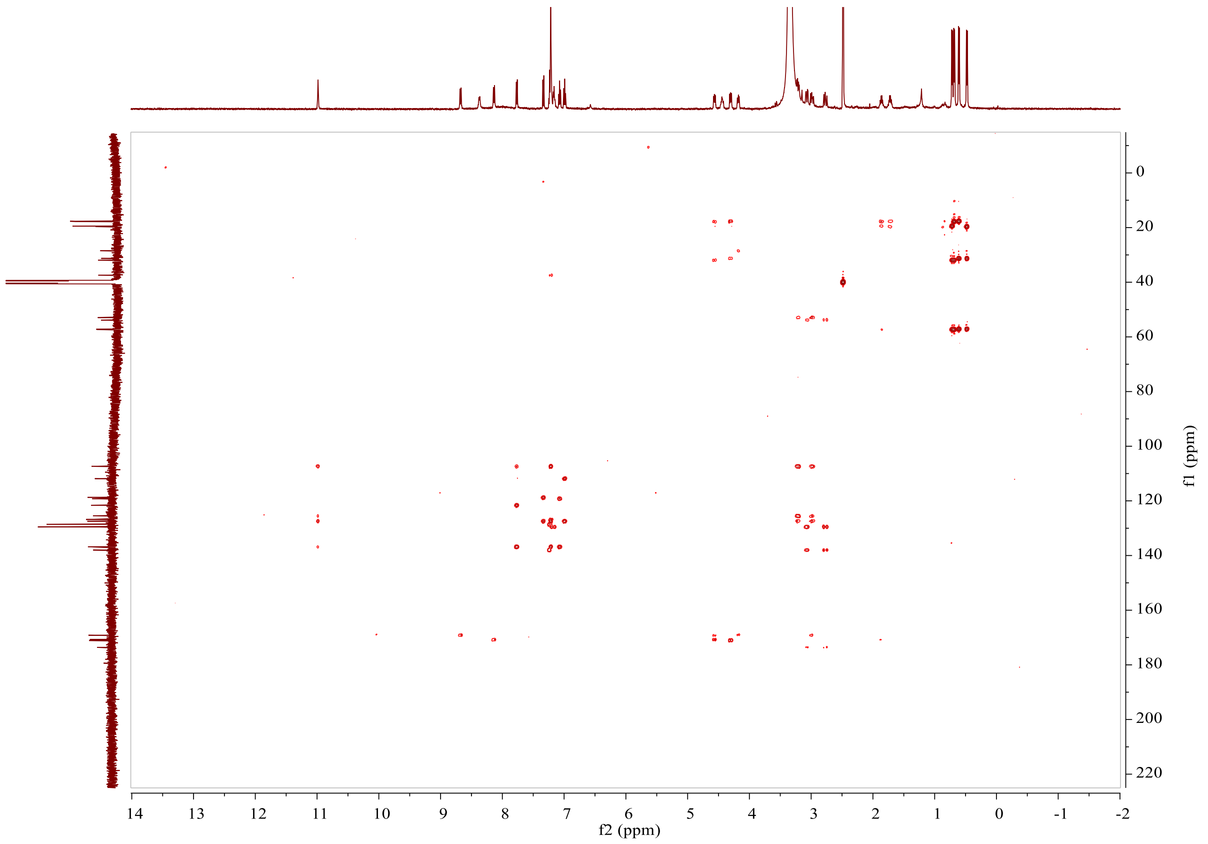

Figure S8. HMBC spectrum (600 MHz, DMSO-d_6) of compound **1**

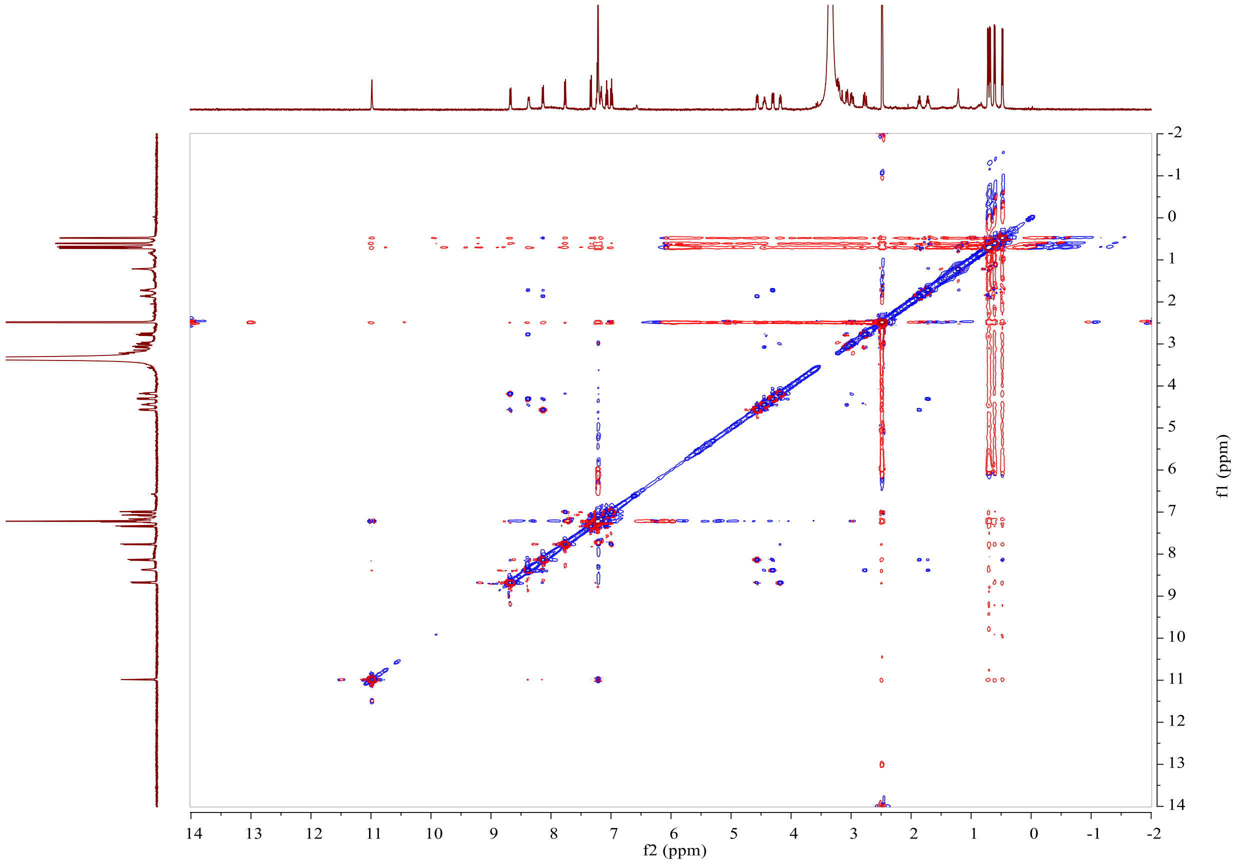

Figure S9. NOESY spectrum (600 MHz, DMSO-d_6) of compound **1**

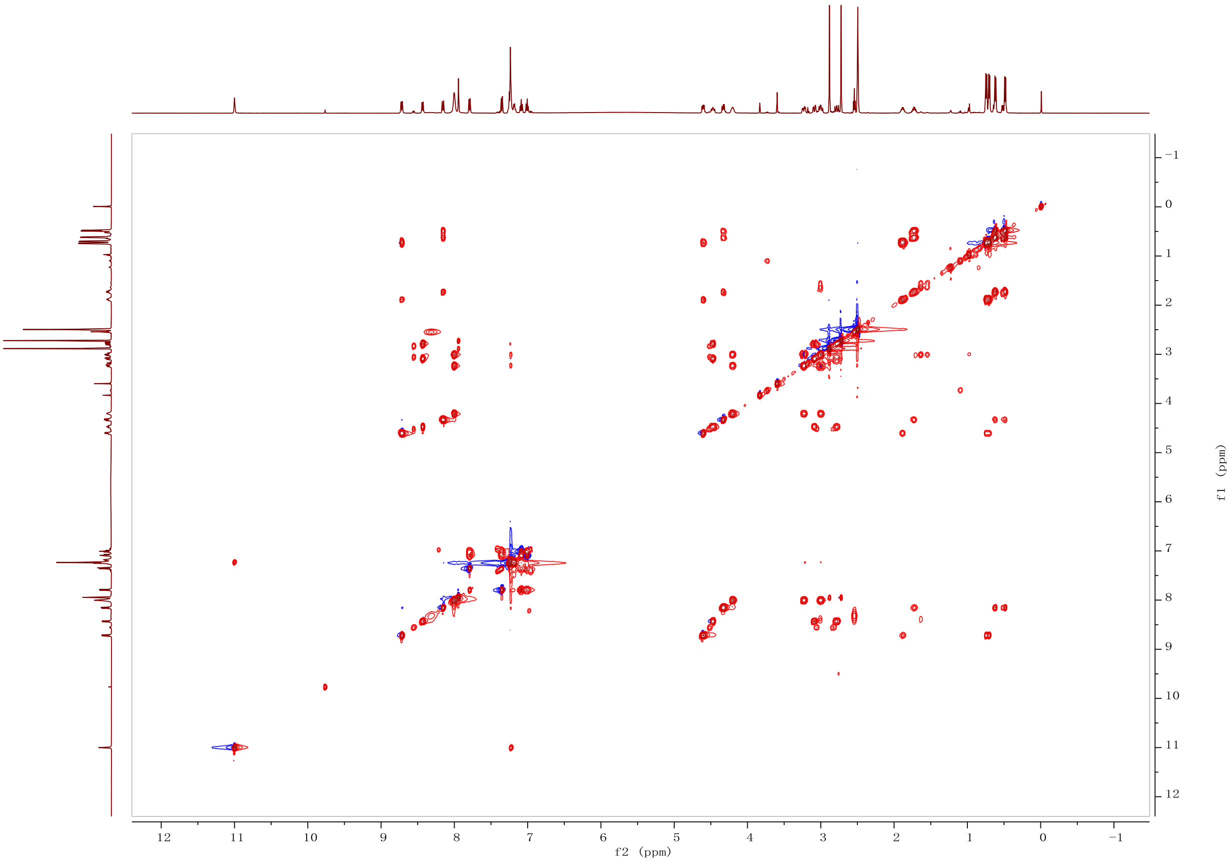

Figure S10. TOCSY spectrum (600 MHz, DMSO-d_6) of compound **1**

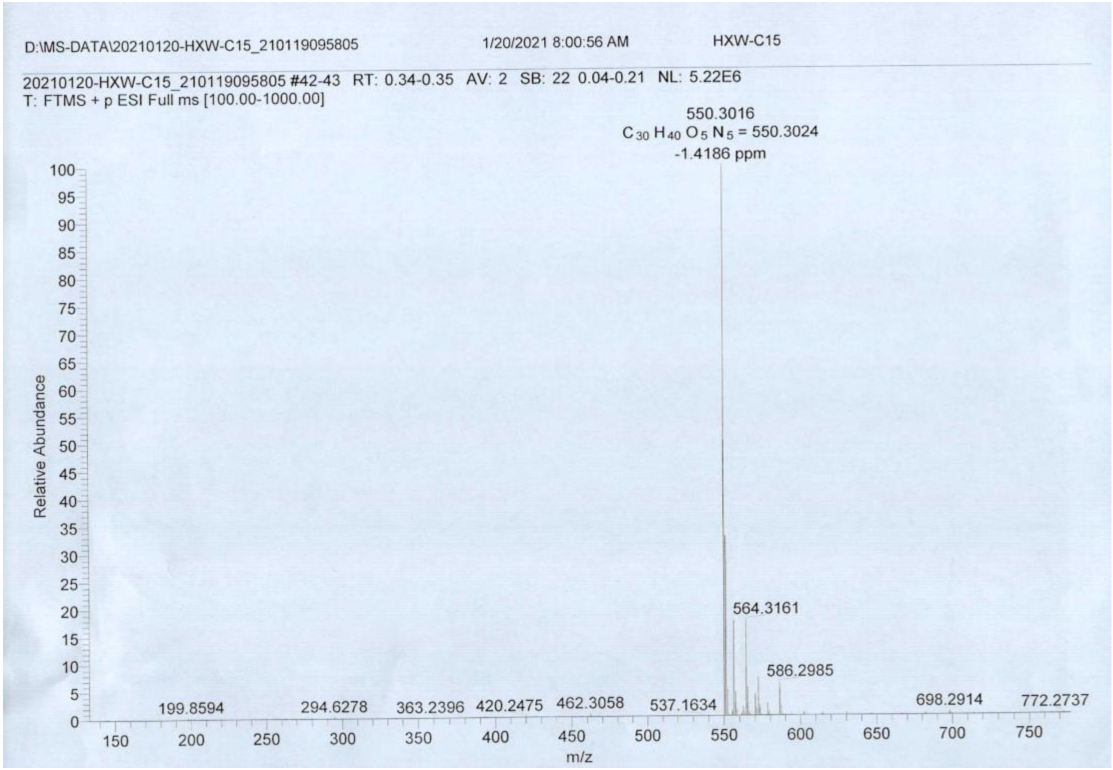

Figure S11. HRESIMS spectrum of compound **1**.

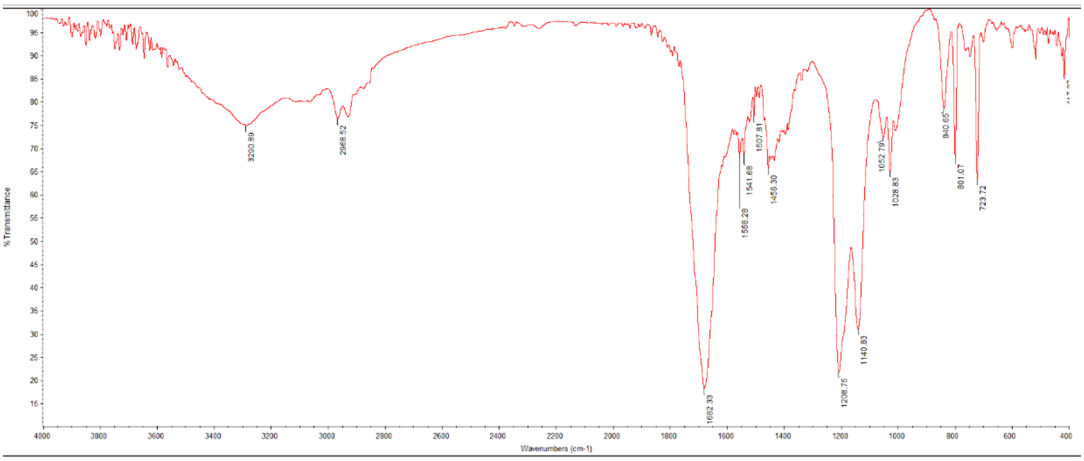

Figure S12. IR spectrum of compound **1.**

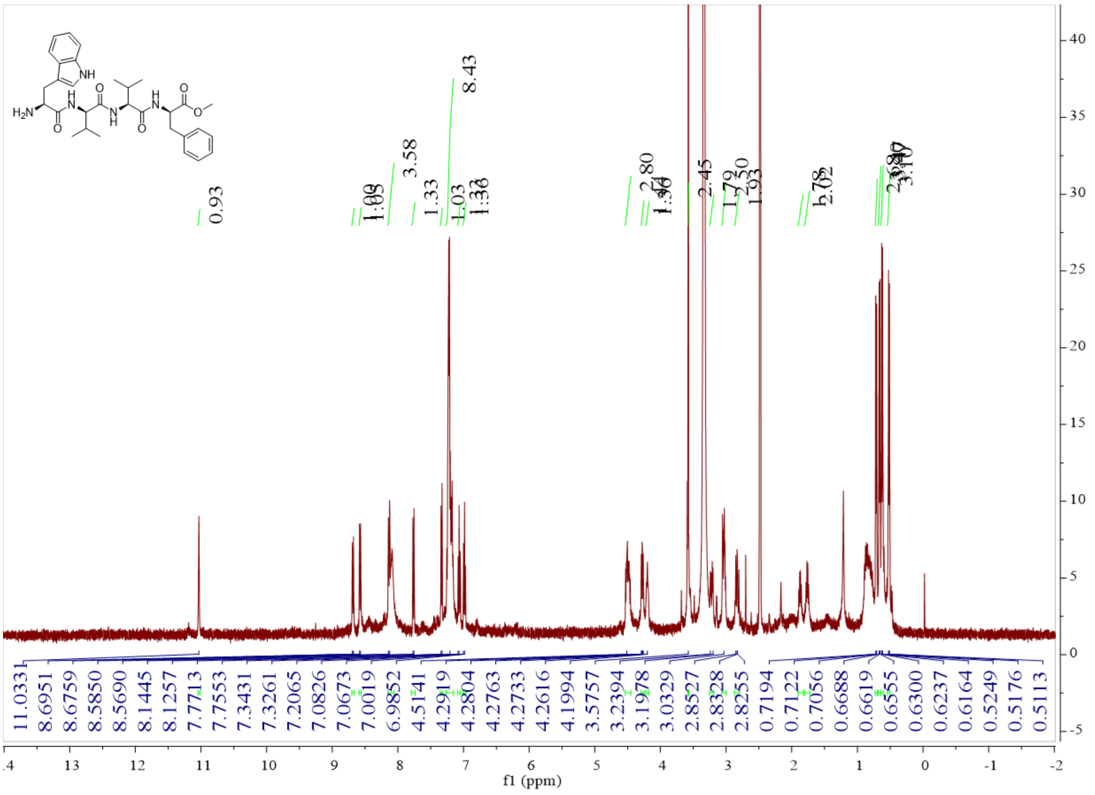

Figure S13. ^{1}H-NMR spectrum (600 MHz, DMSO-d_6) of compound **2**

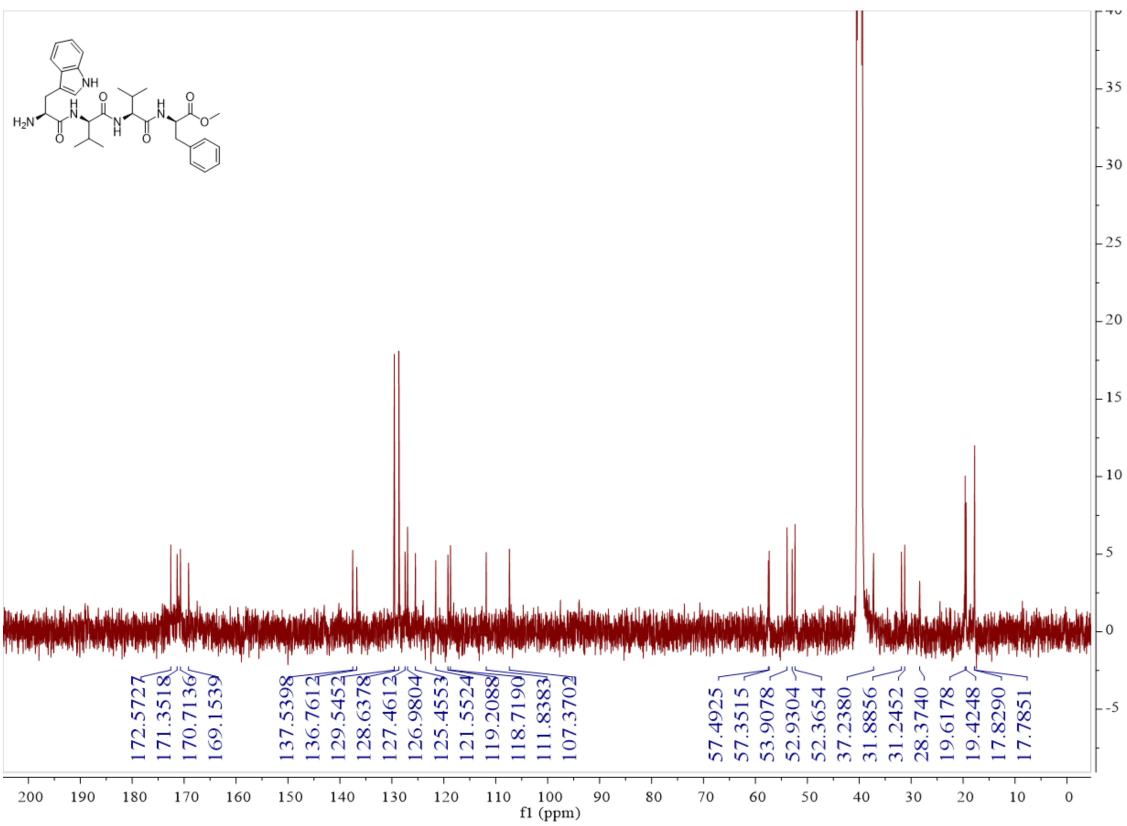

Figure S14. ^{13}C-NMR spectrum (150 MHz, DMSO-d_6) of compound **2**

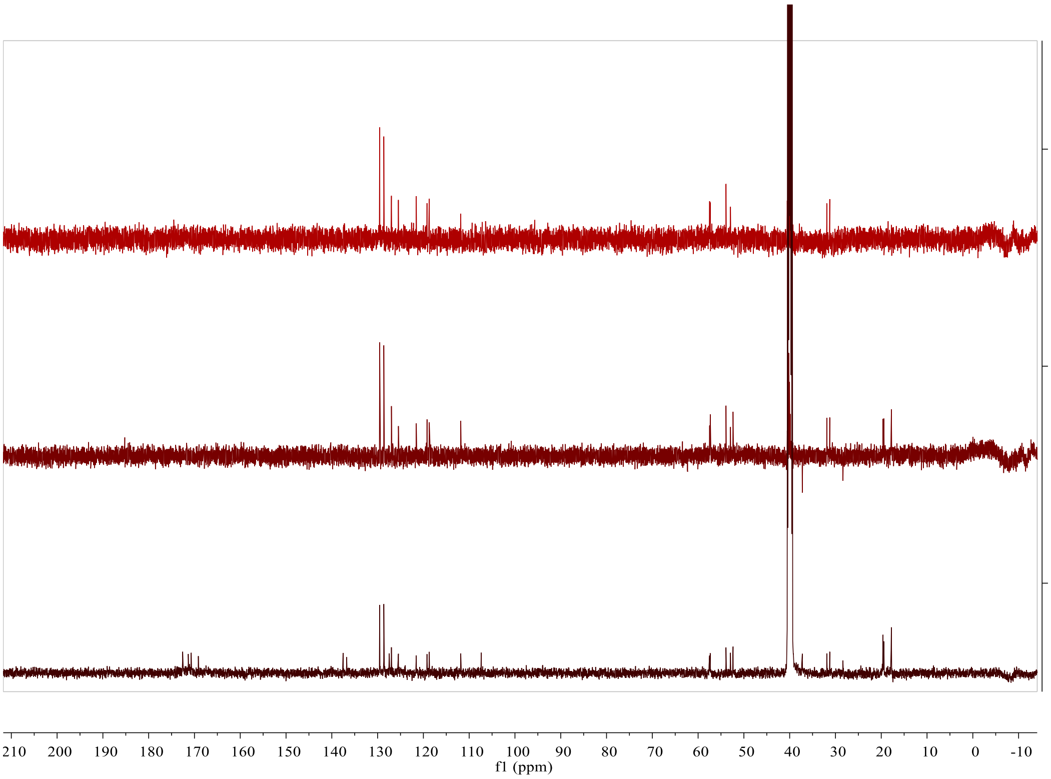

Figure S15. DEPT (150 MHz, DMSO-d_6) spectrum of compound **2**

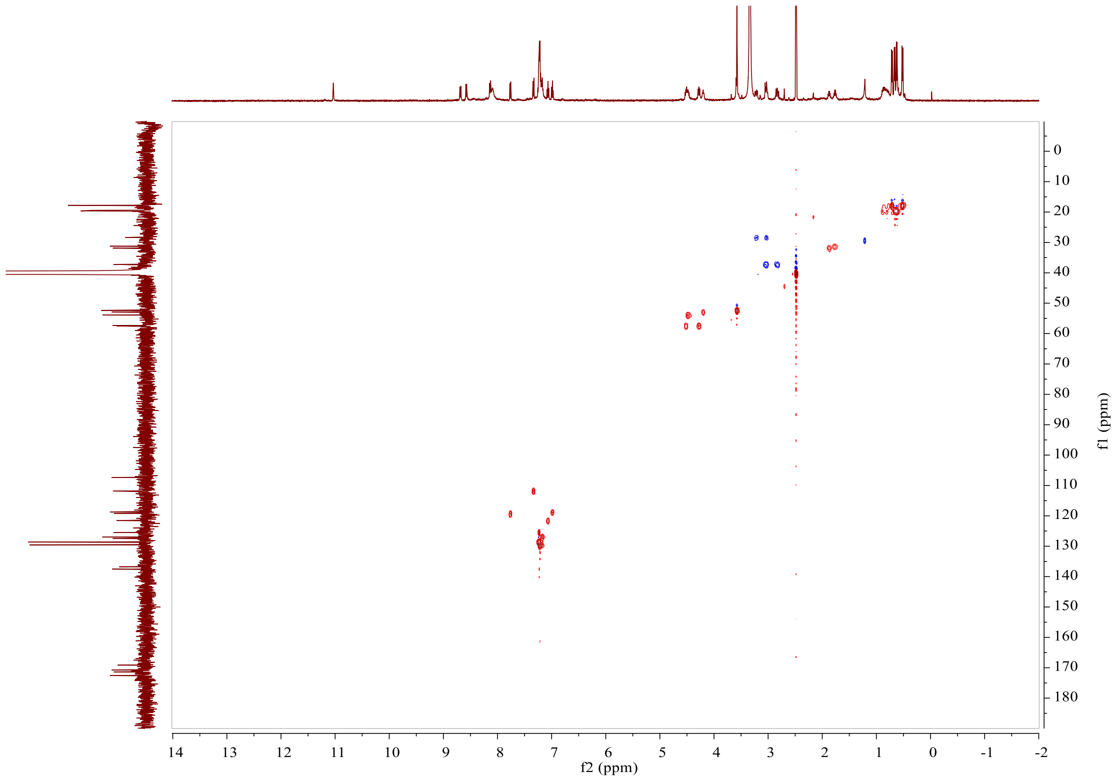

Figure S16. HSQC spectrum (600 MHz, DMSO-d_6) of compound **2**

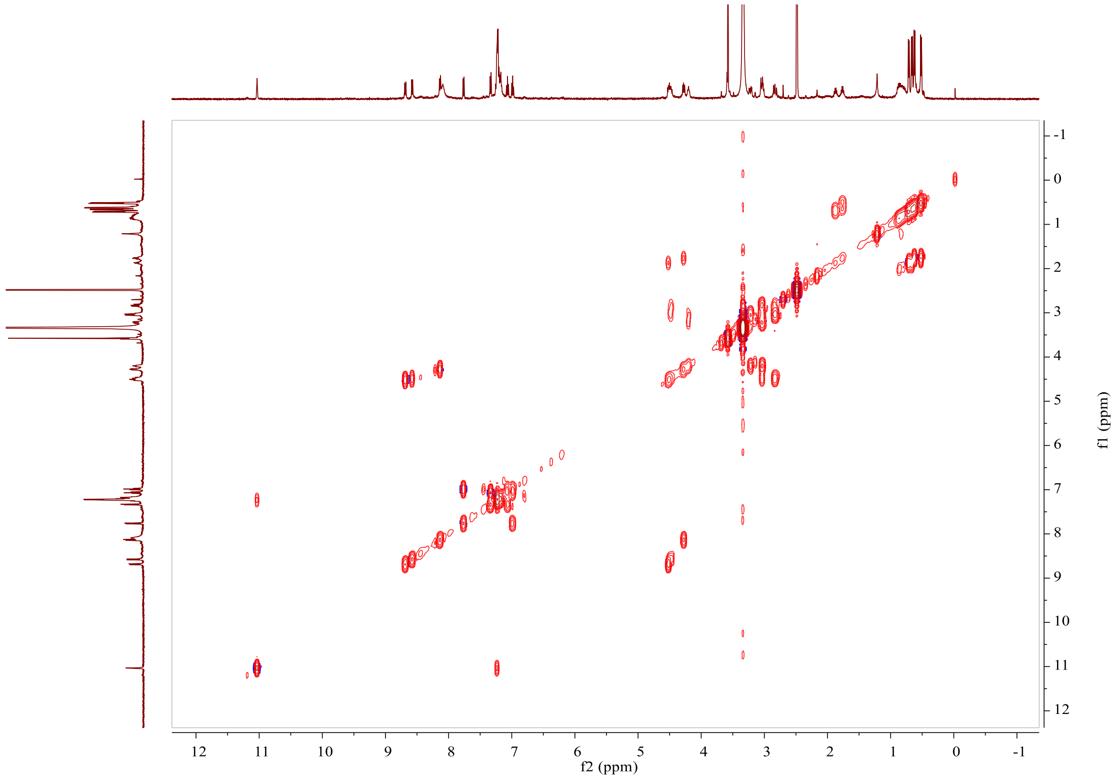

Figure S17. ^{1}H-^{1}H COSY spectrum (600 MHz, DMSO-d_6) of compound **2**

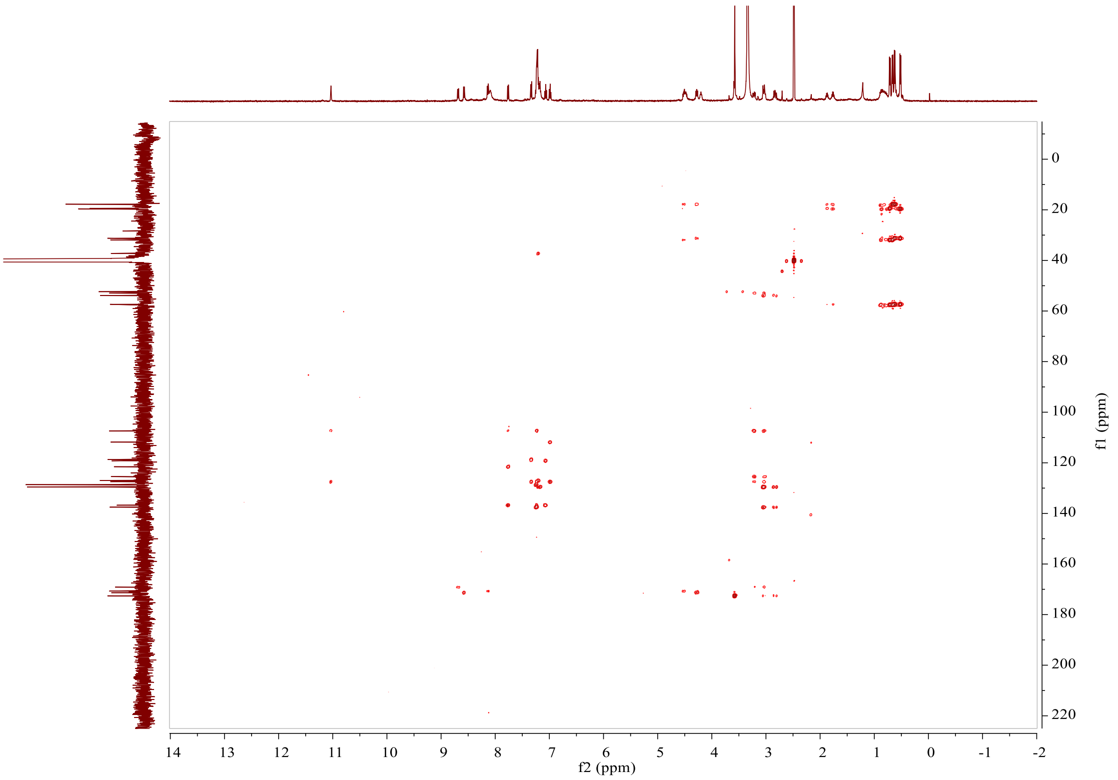

Figure S18. HMBC spectrum (600 MHz, DMSO-d_6) of compound **2**

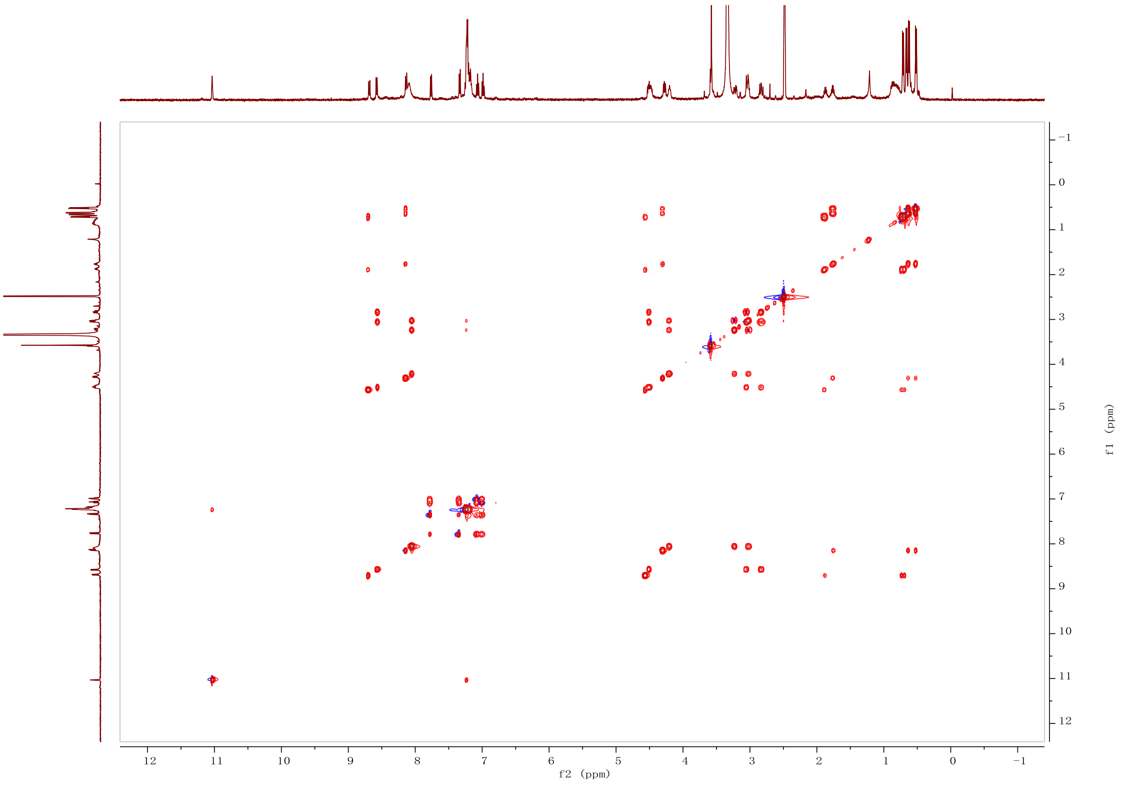

Figure S19. TOCSY spectrum (600 MHz, DMSO-d_6) of compound **2**

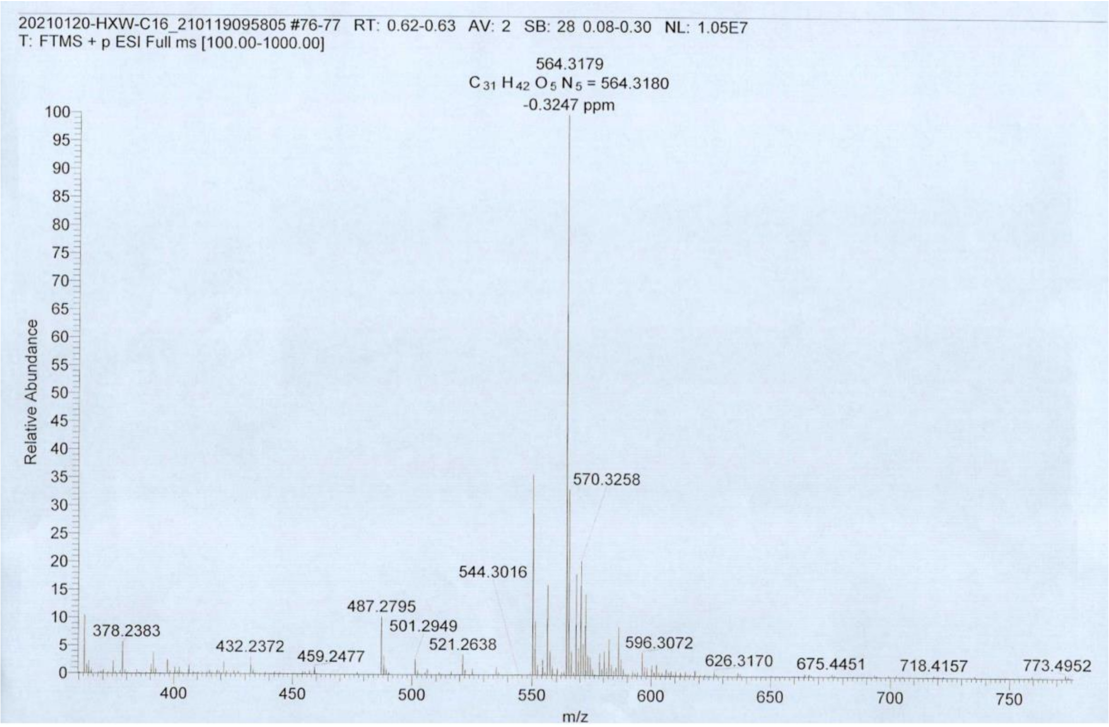

Figure S20. HRESIMS spectrum of compound **2**

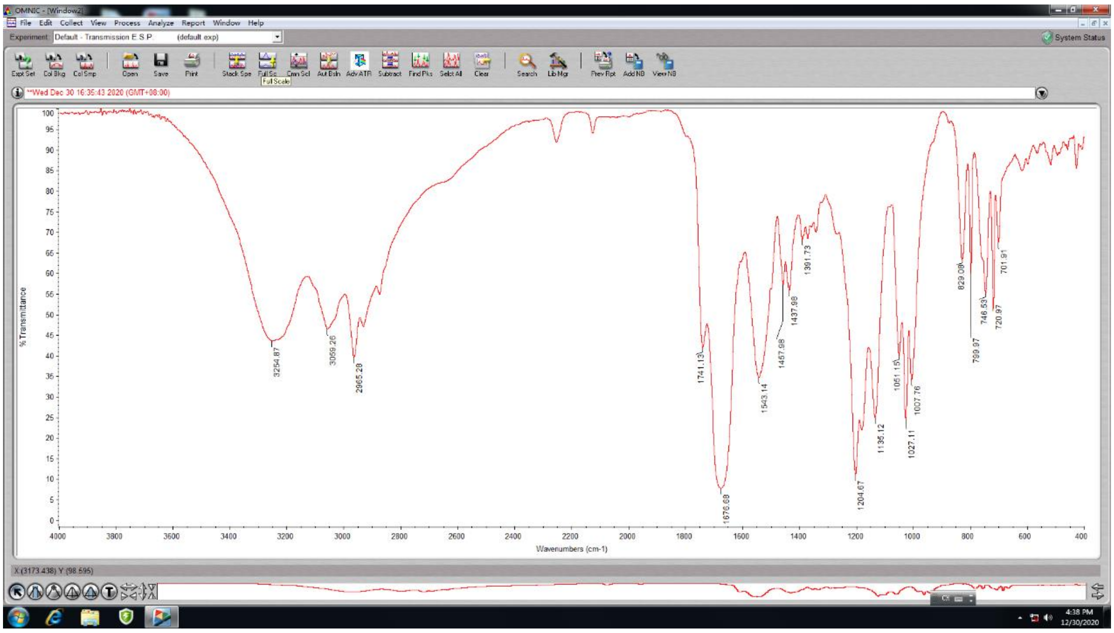

Figure S21. IR spectrum of compound **2**

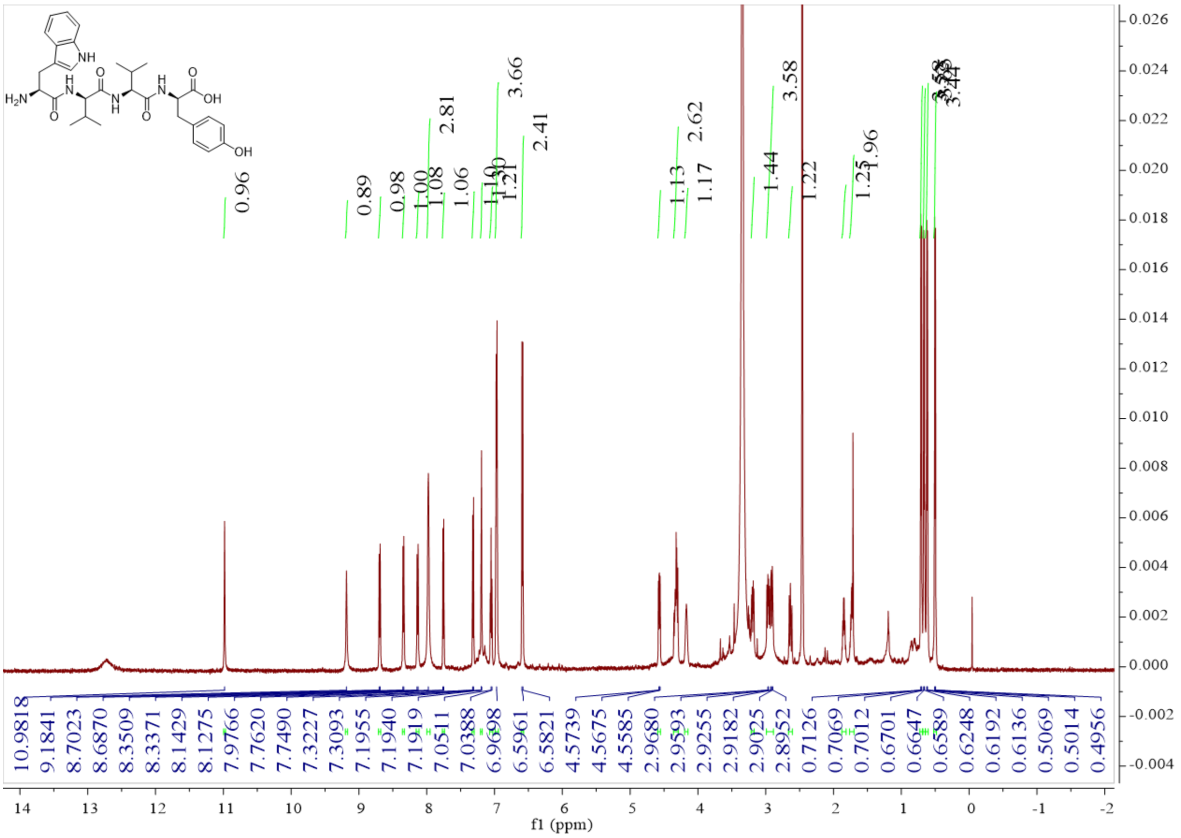

Figure S22. ^{1}H-NMR spectrum (600 MHz, DMSO-d_6) of compound **3**

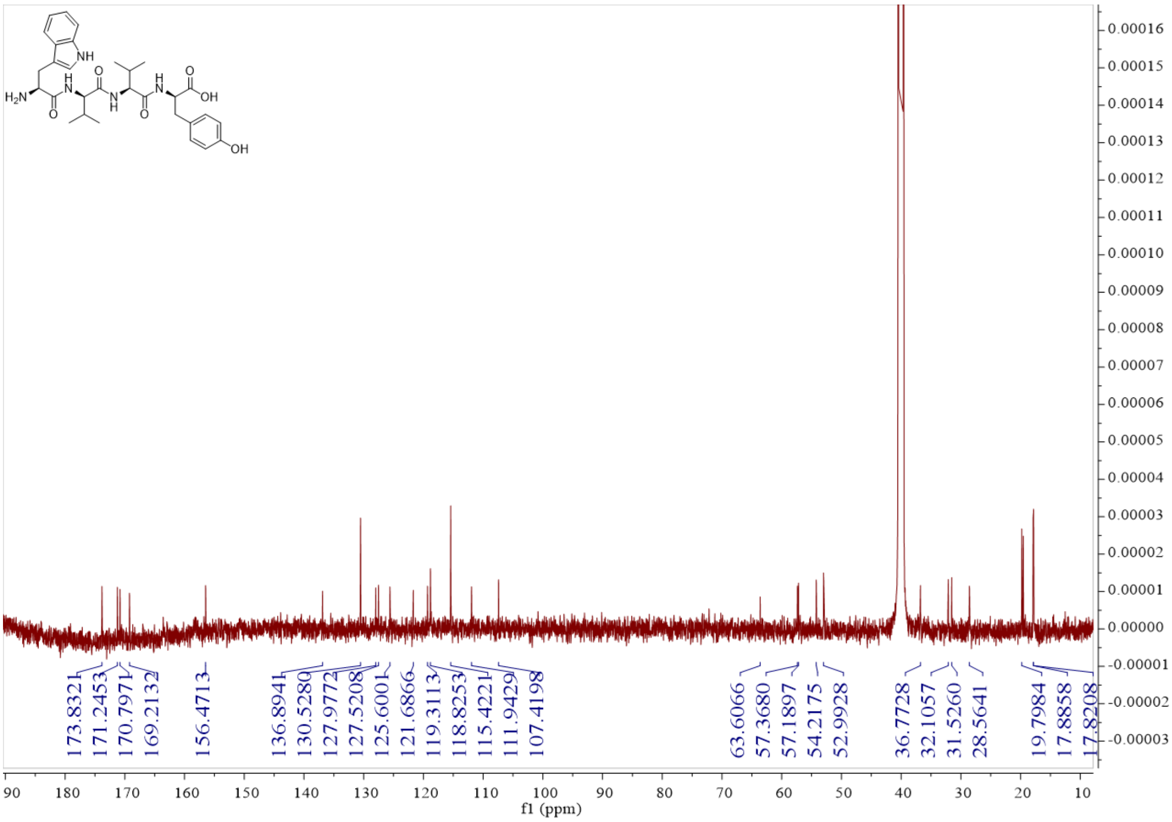

Figure S23. ^{13}C-NMR spectrum (150 MHz, DMSO-d_6) of compound **3**

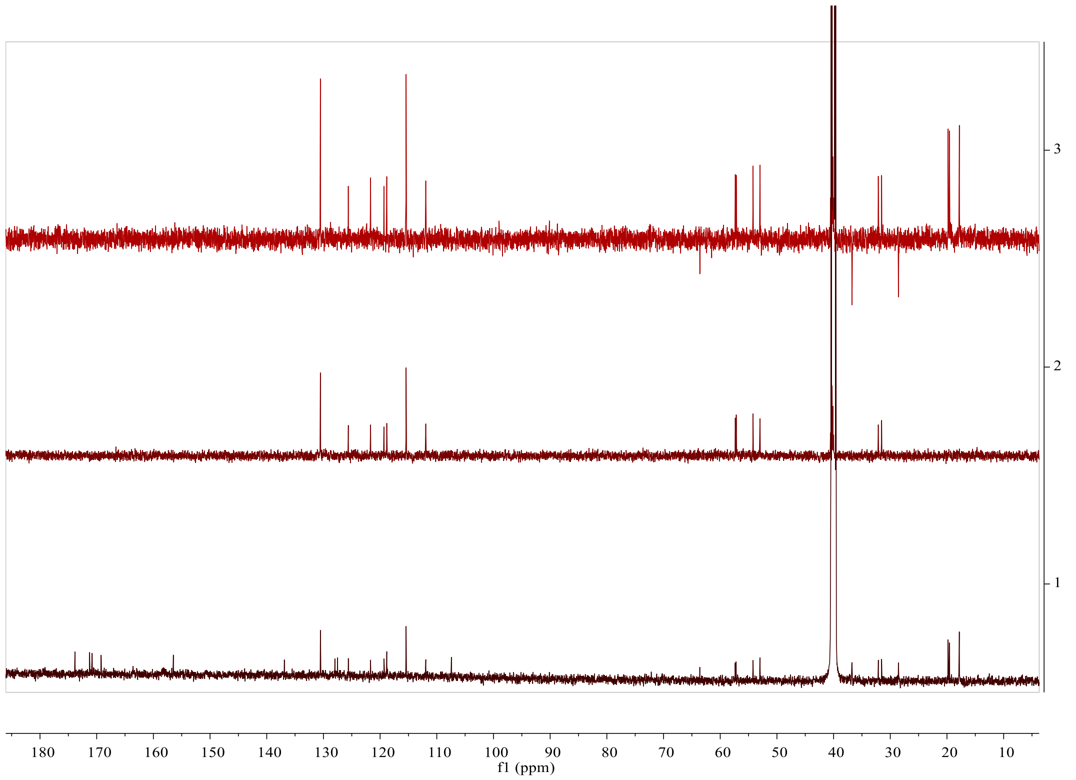

Figure S24. DEPT (150 MHz, DMSO-d_6) spectrum of compound **3**

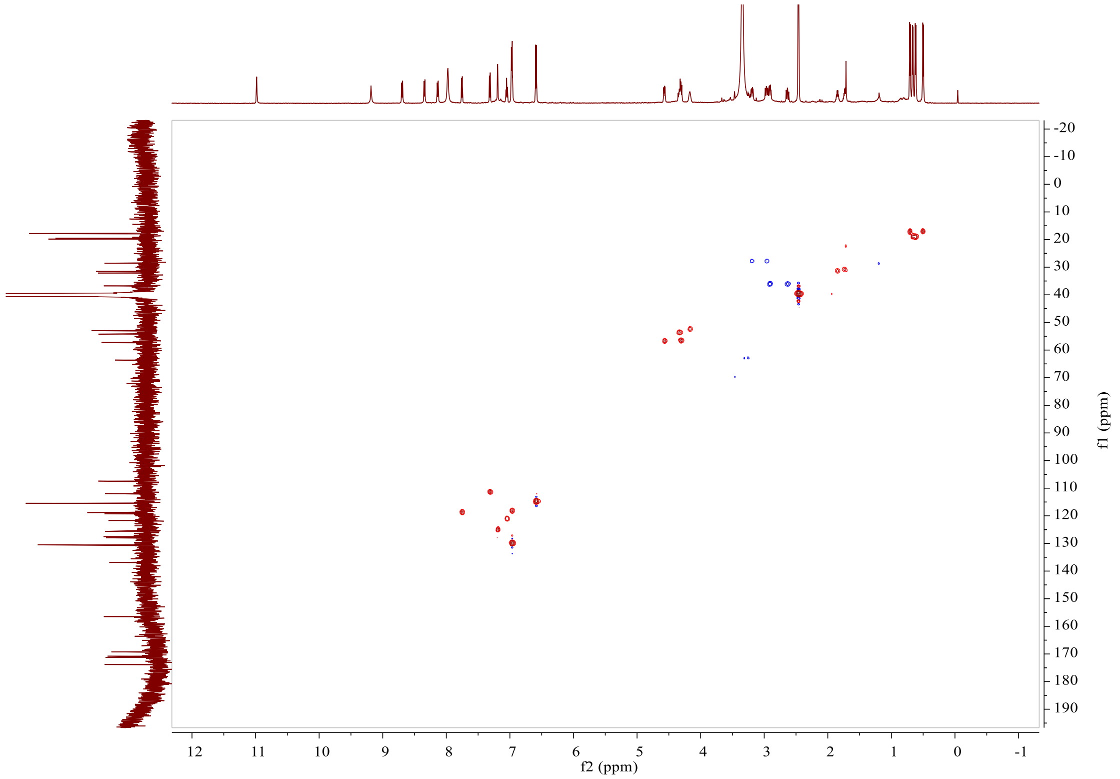

Figure S25. HSQC spectrum (600 MHz, DMSO-d_6) of compound **3**

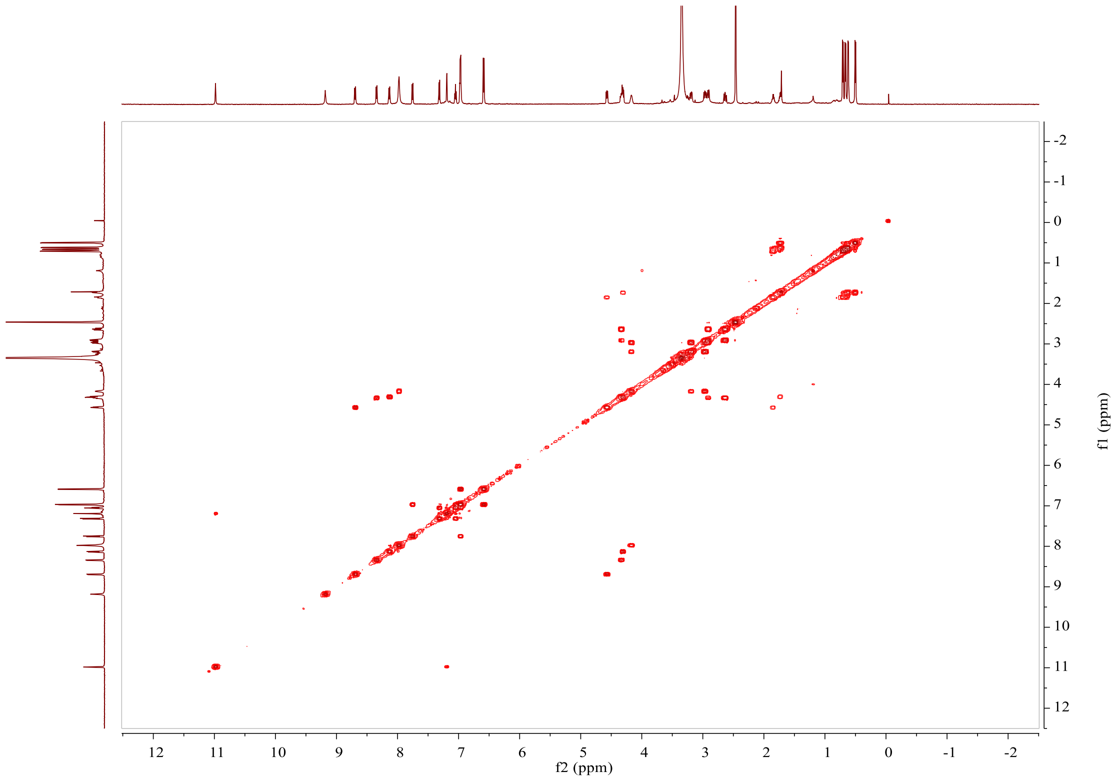

Figure S26. ^{1}H-^{1}H COSY spectrum (600 MHz, DMSO-d_6) of compound **3**

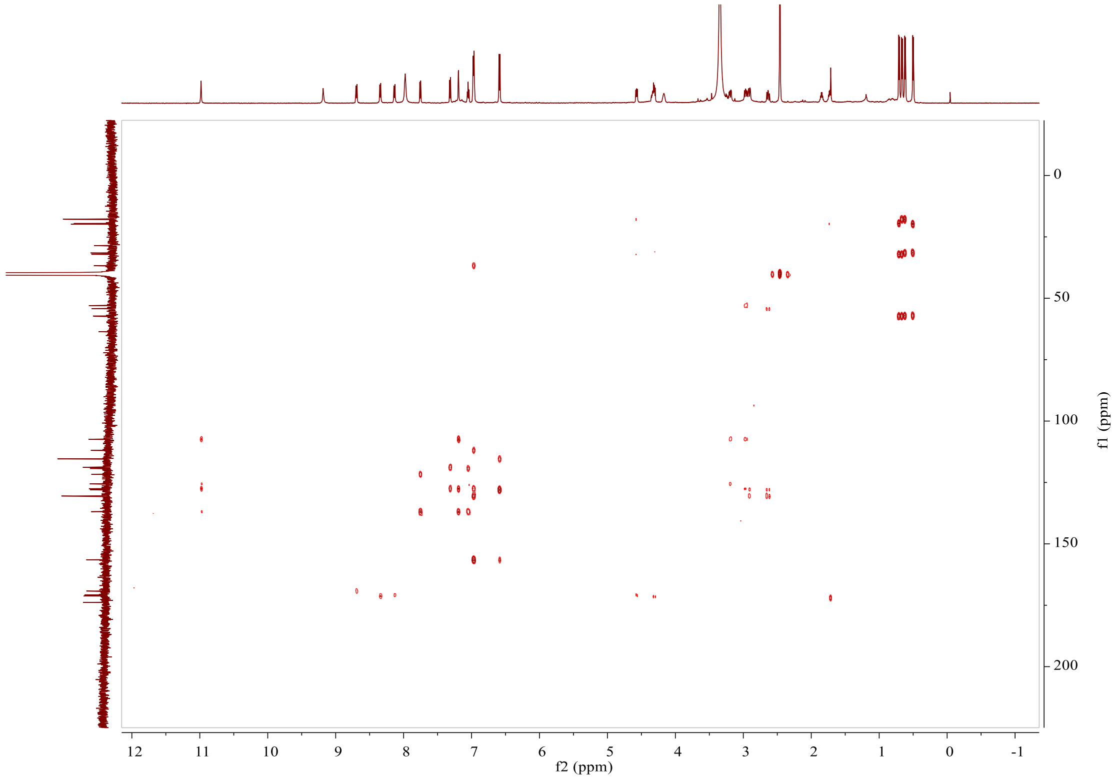

Figure S27. HMBC spectrum (600 MHz, DMSO-d_6) of compound **3**

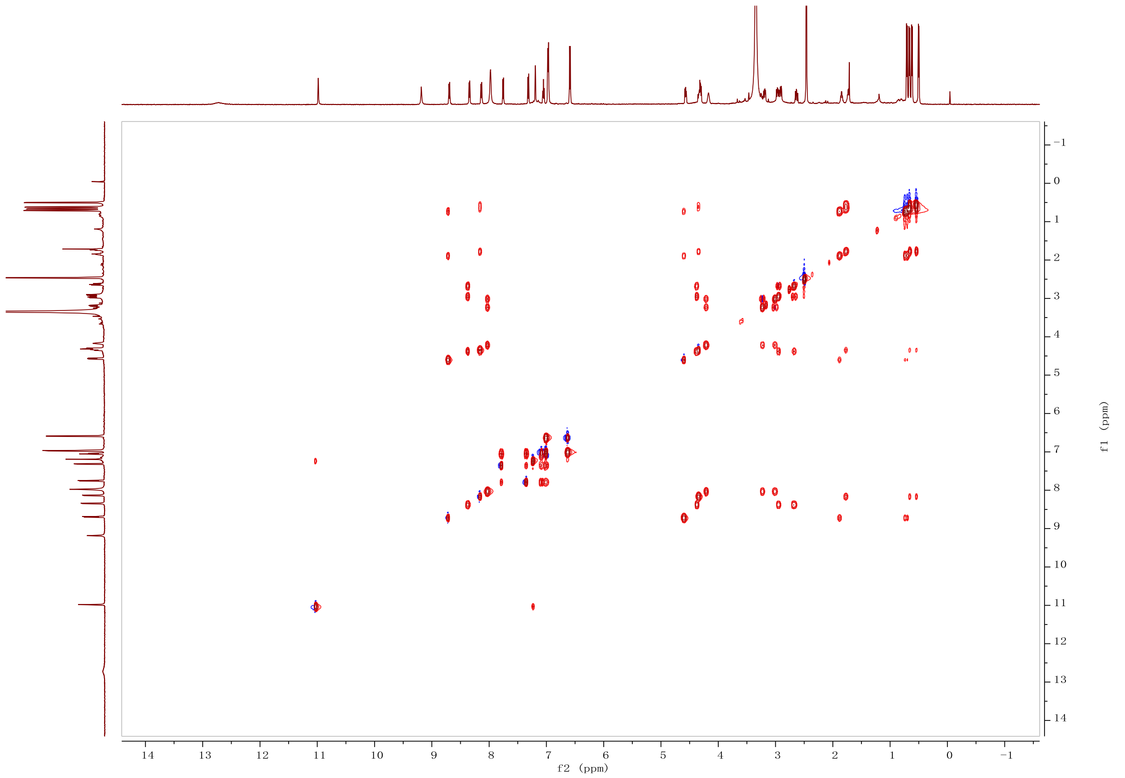

Figure S28. TOCSY spectrum (600 MHz, DMSO-d_6) of compound **3**

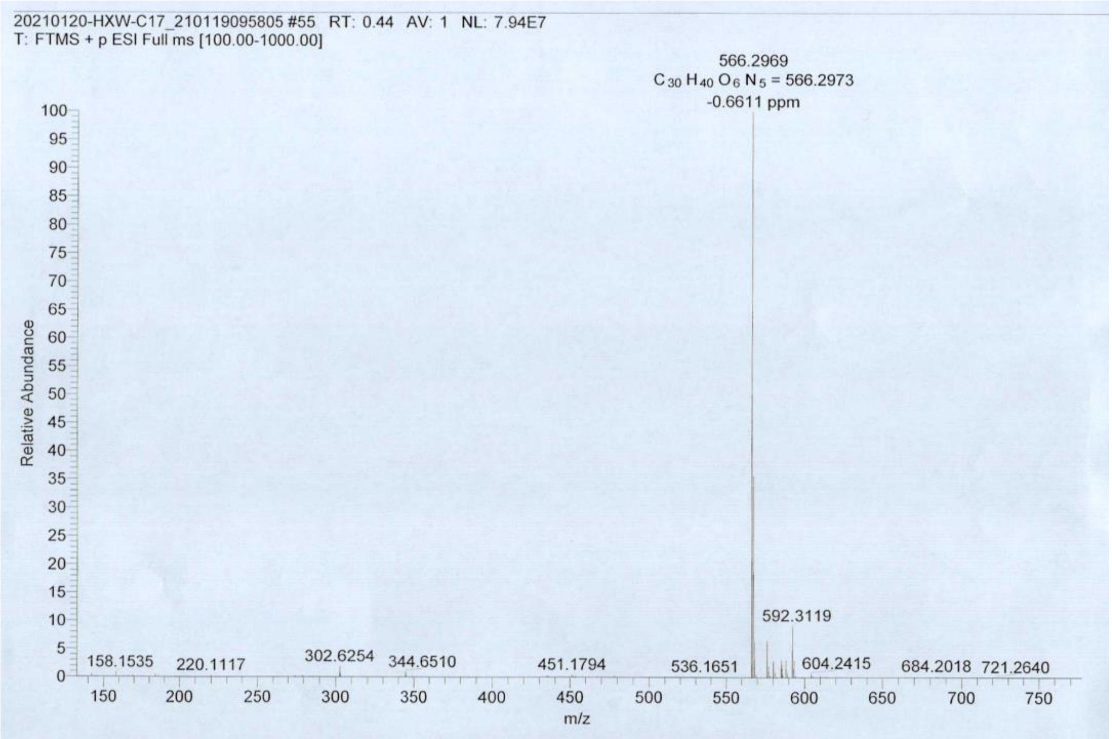

Figure S29. HRESIMS spectrum of compound **3**

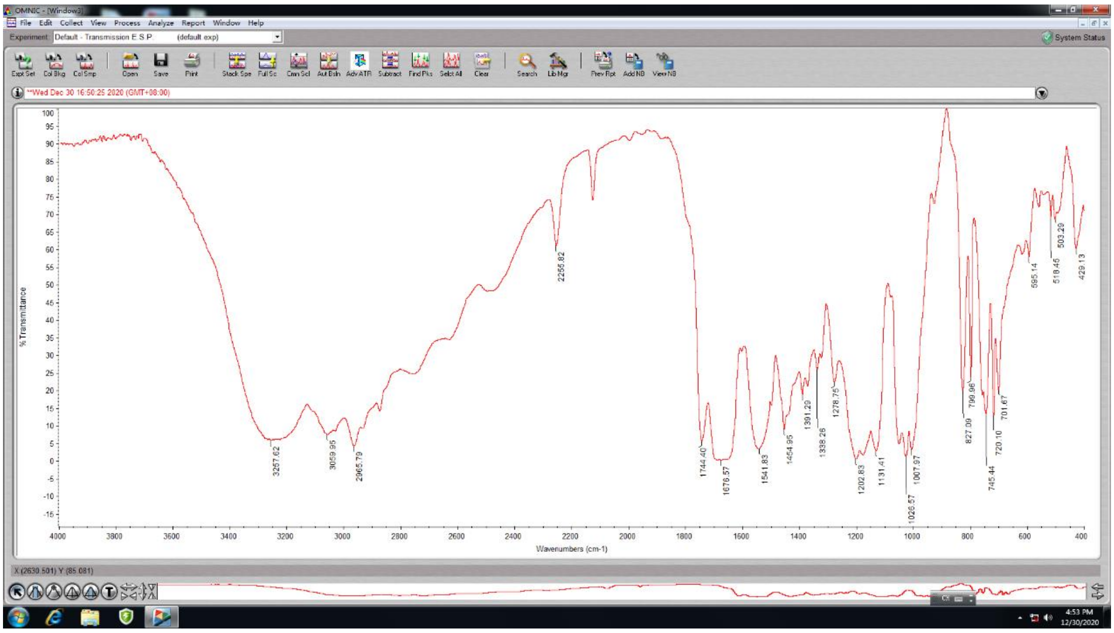

Figure S30. IR spectrum of compound **3**

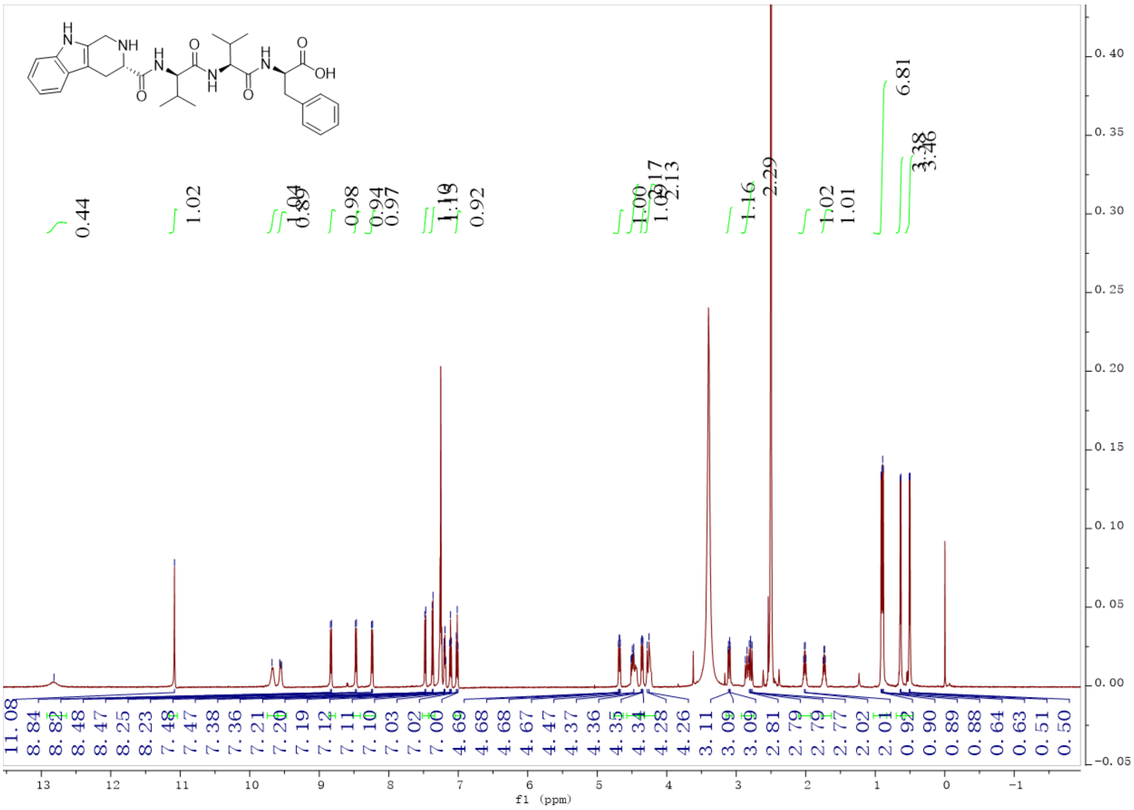

Figure S31. ^{1}H-NMR spectrum (600 MHz, DMSO-d_6) of compound **4**

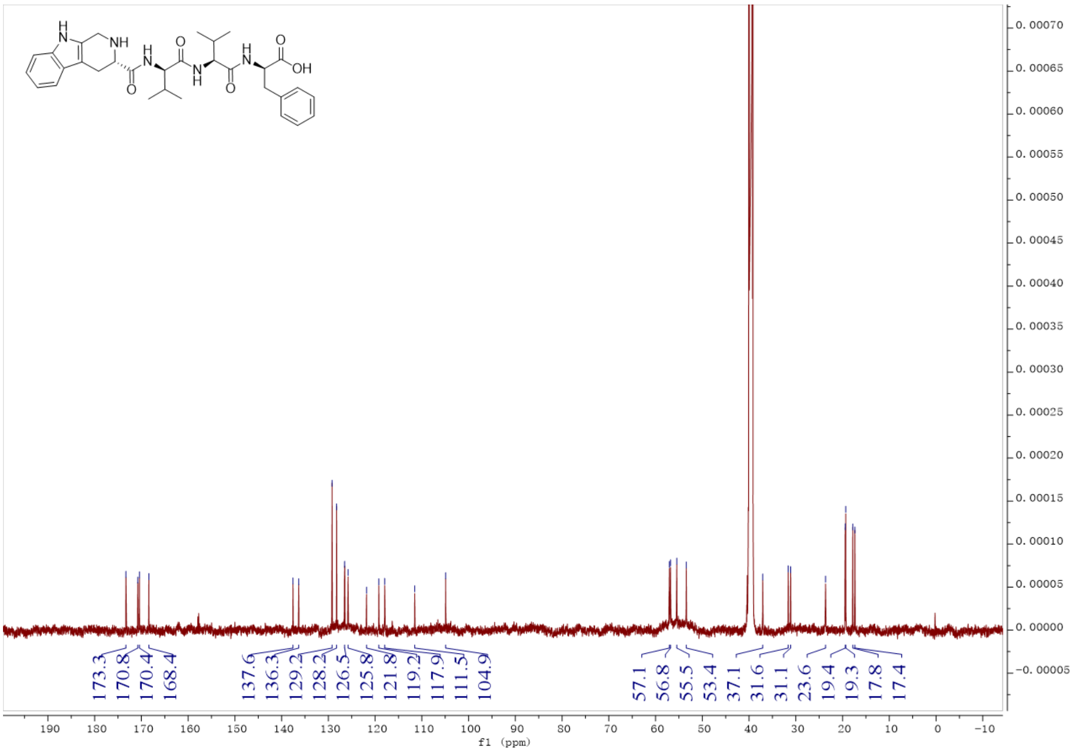

Figure S32. ^{13}C-NMR spectrum (150 MHz, DMSO-d_6) of compound **4**

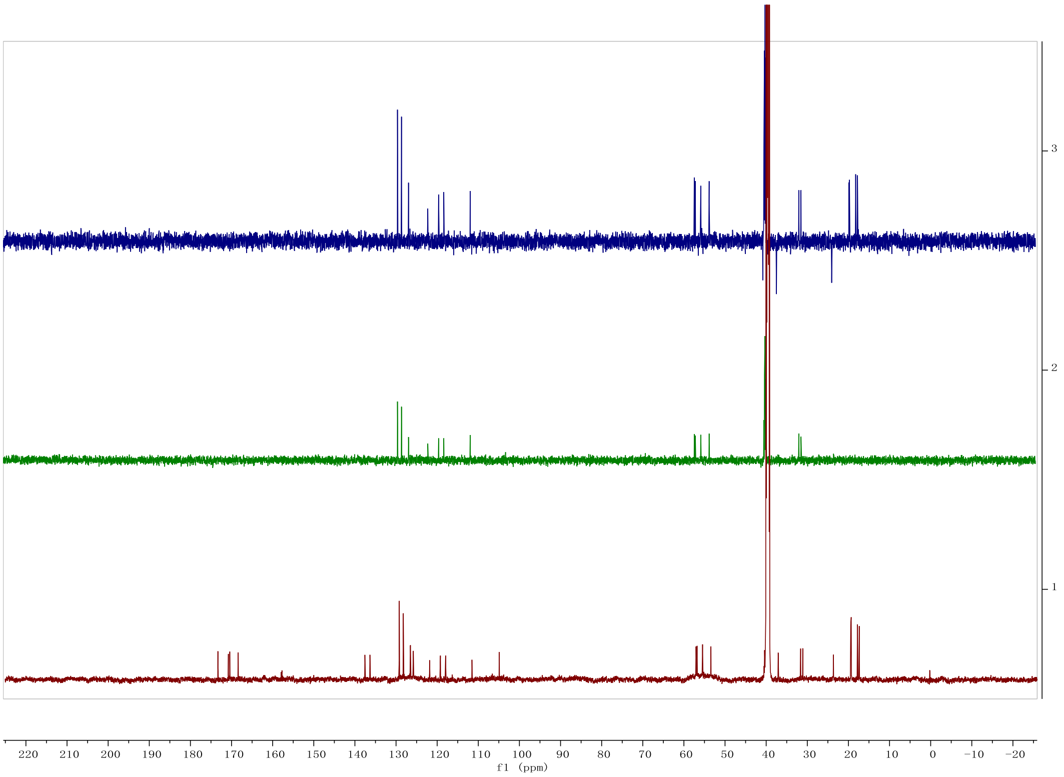

Figure S33. DEPT (150 MHz, DMSO-d_6) spectrum of compound **4**

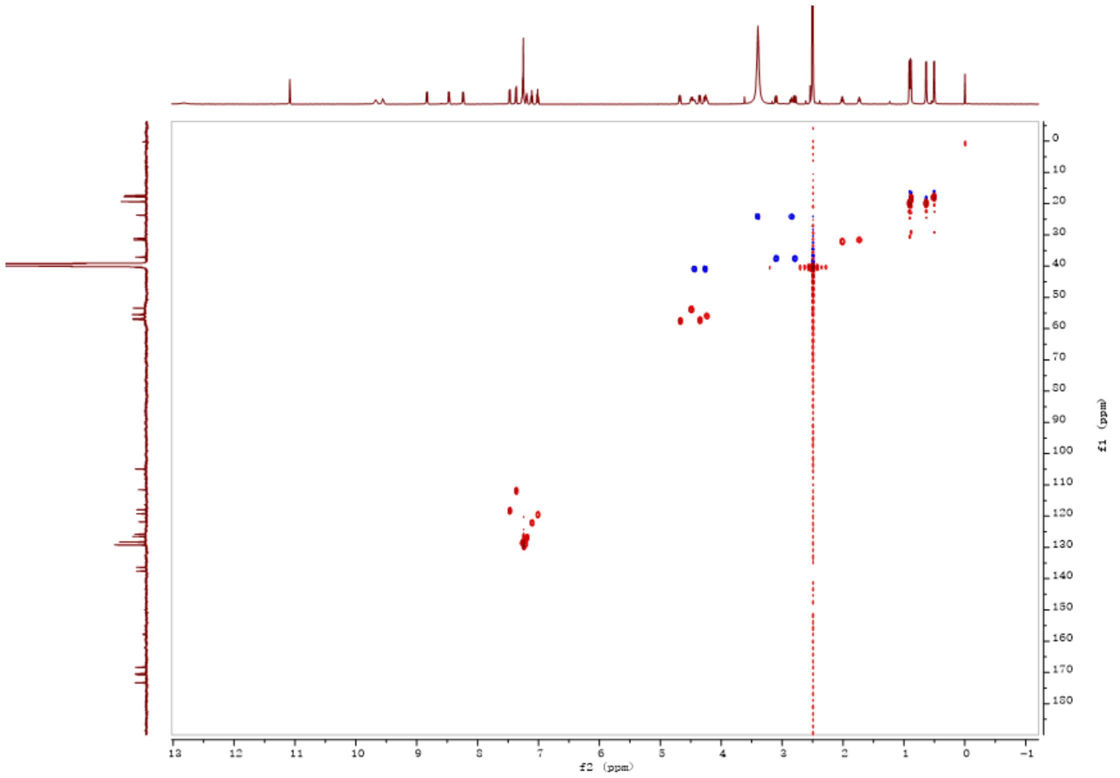

Figure S34. HSQC spectrum (600 MHz, DMSO-d_6) of compound **4**

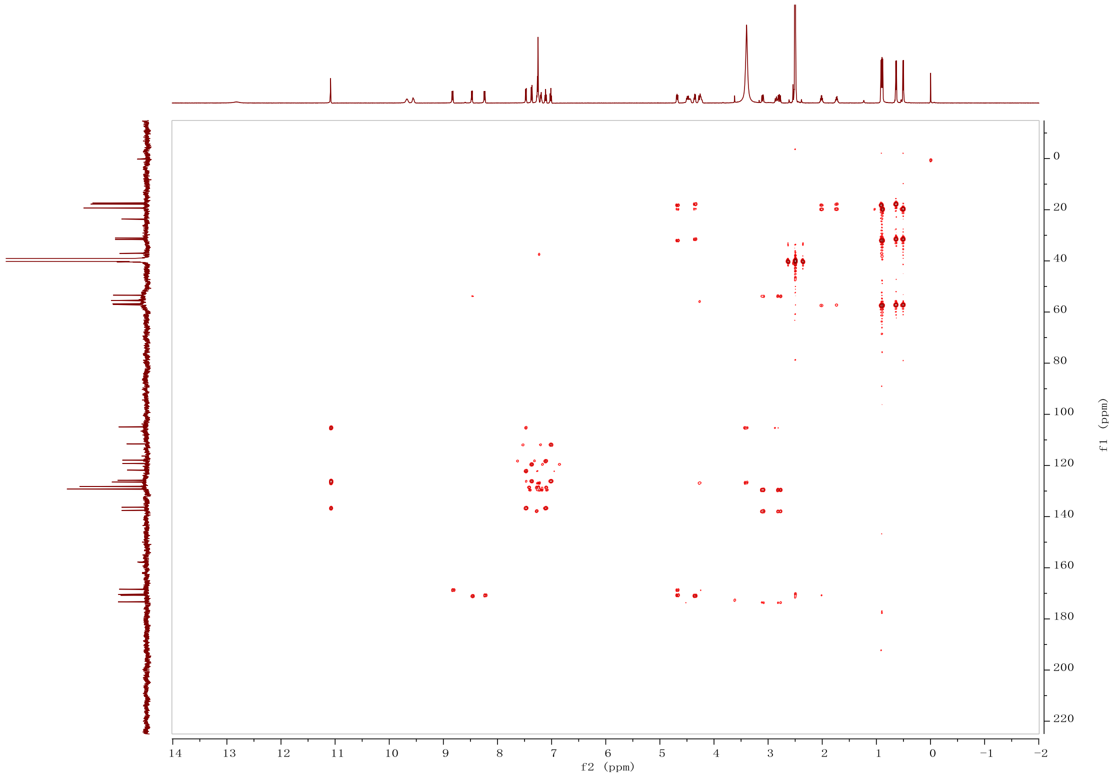

Figure S35. HMBC spectrum (600 MHz, DMSO-d_6) of compound **4**

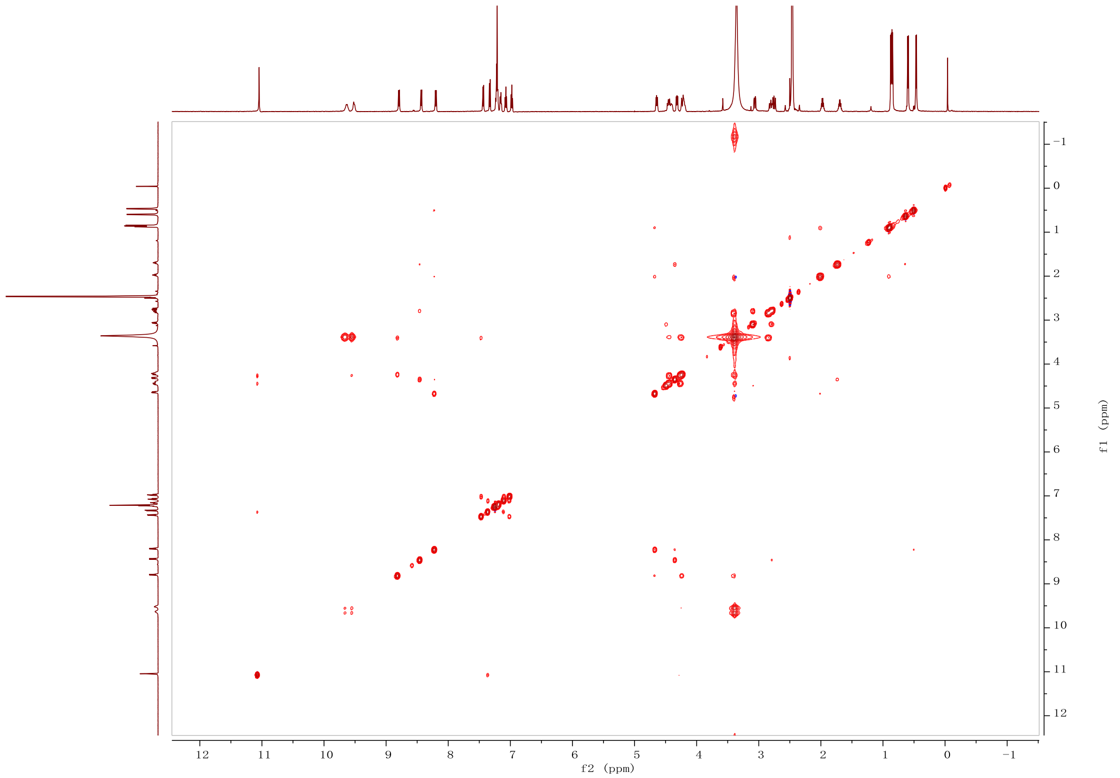

Figure S36. NOESY spectrum (600 MHz, DMSO-d_6) of compound **4**

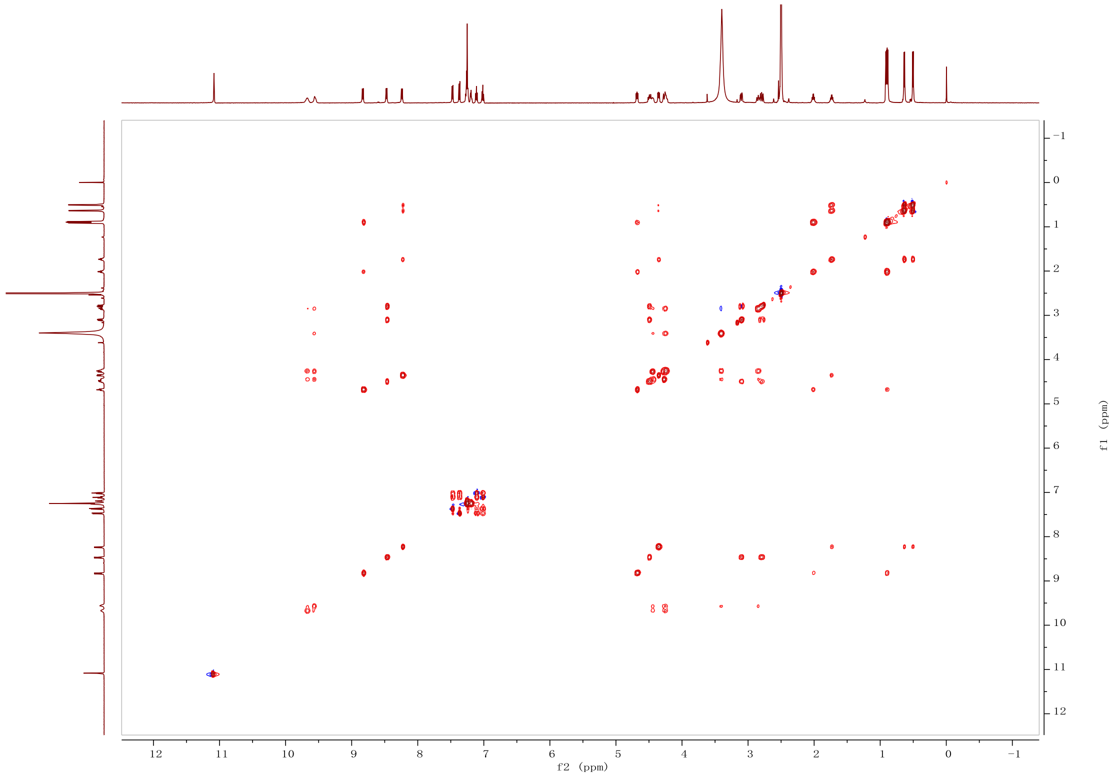

Figure S37. TOCSY spectrum (600 MHz, DMSO-d_6) of compound **4**

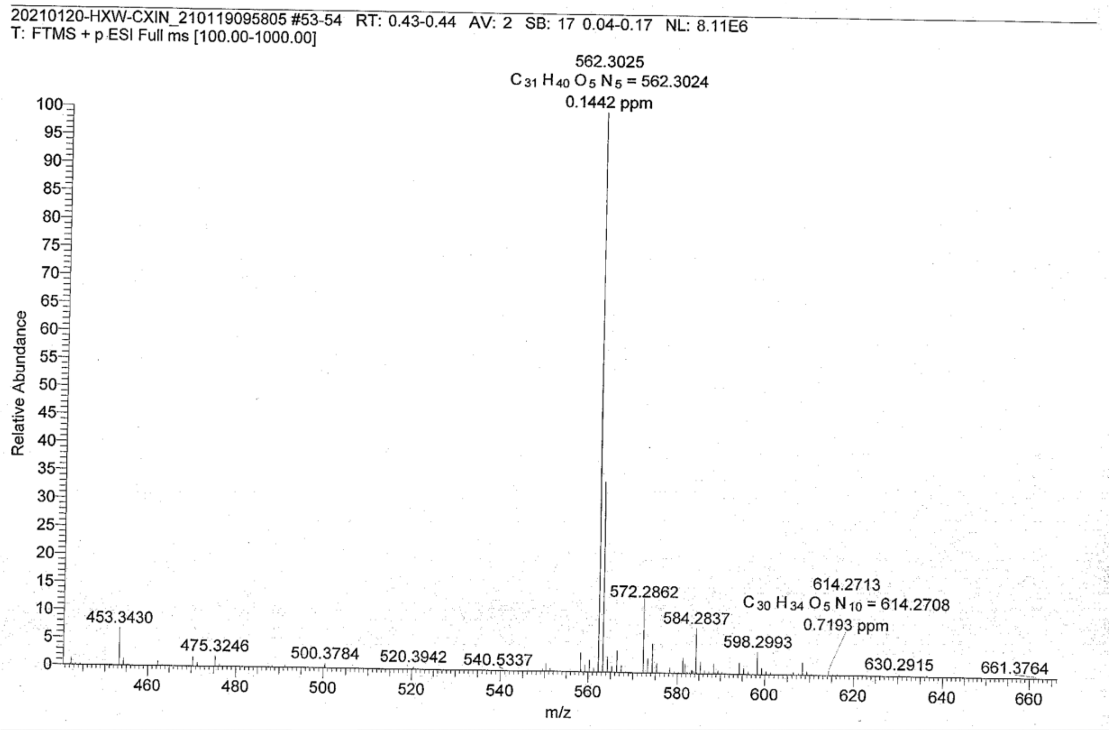

Figure S38. HRESIMS spectrum of compound **4**

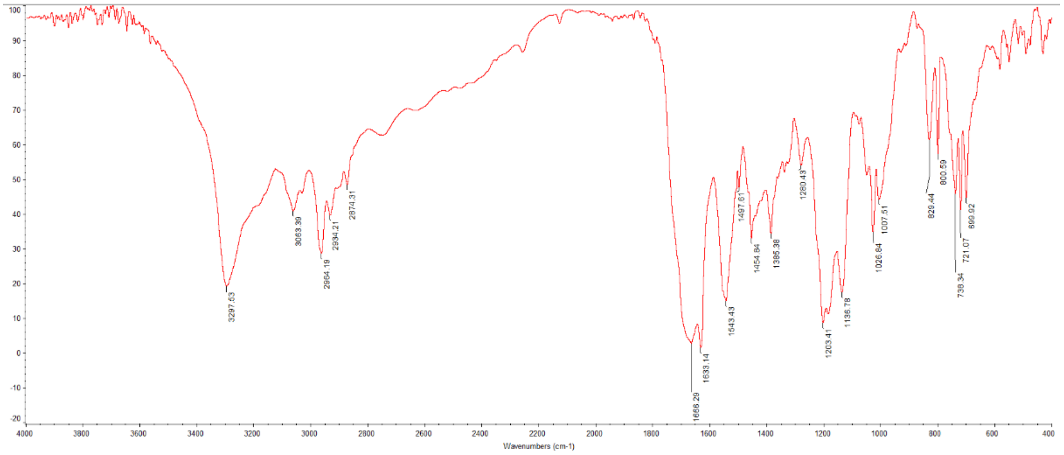

Figure S39. IR spectrum of compound **4**

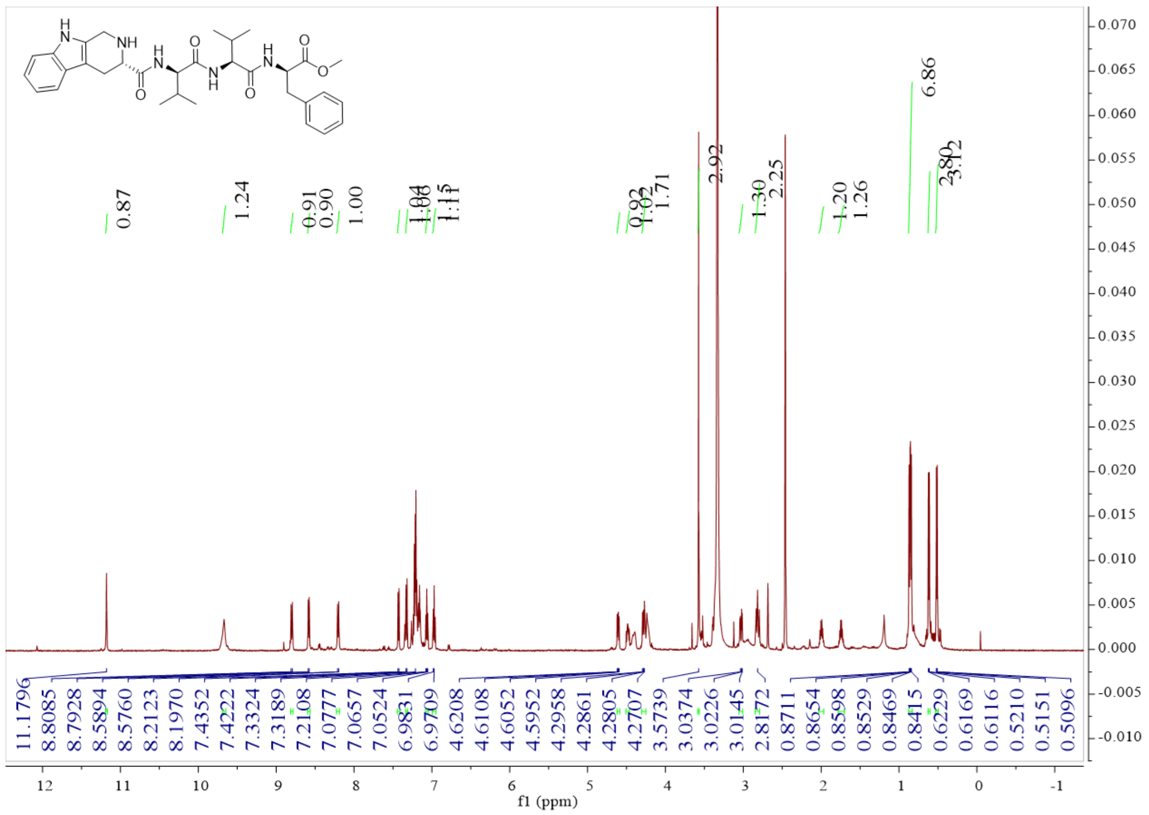

Figure S40. ^{1}H-NMR spectrum (600 MHz, DMSO-d_6) of compound **5**

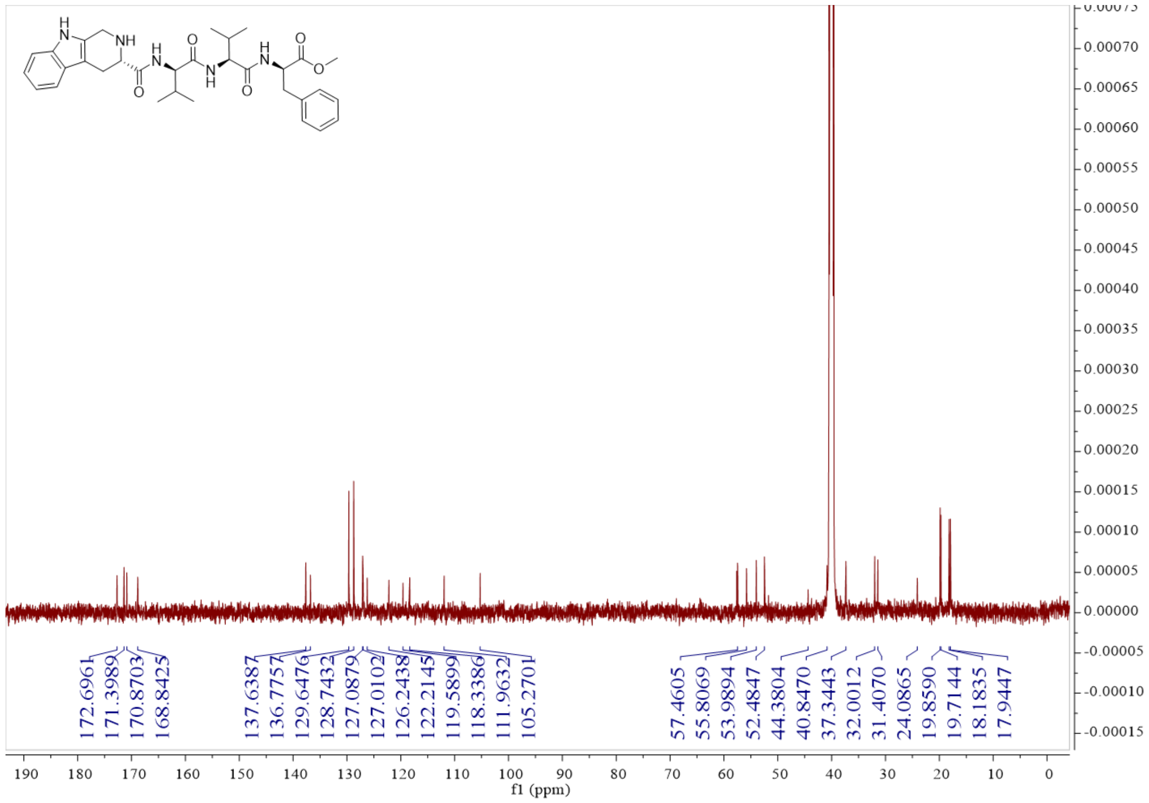

Figure S41. ^{13}C-NMR spectrum (150 MHz, DMSO-d_6) of compound **5**

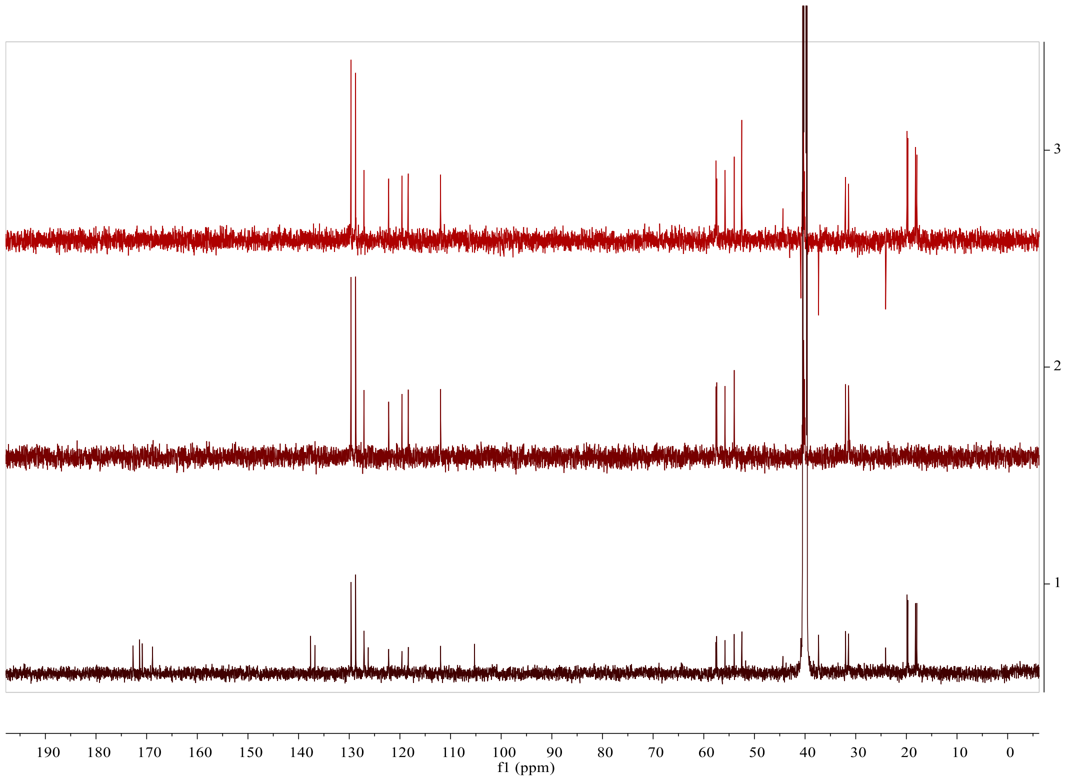

Figure S42. DEPT (150 MHz, DMSO-d_6) spectrum of compound **5**

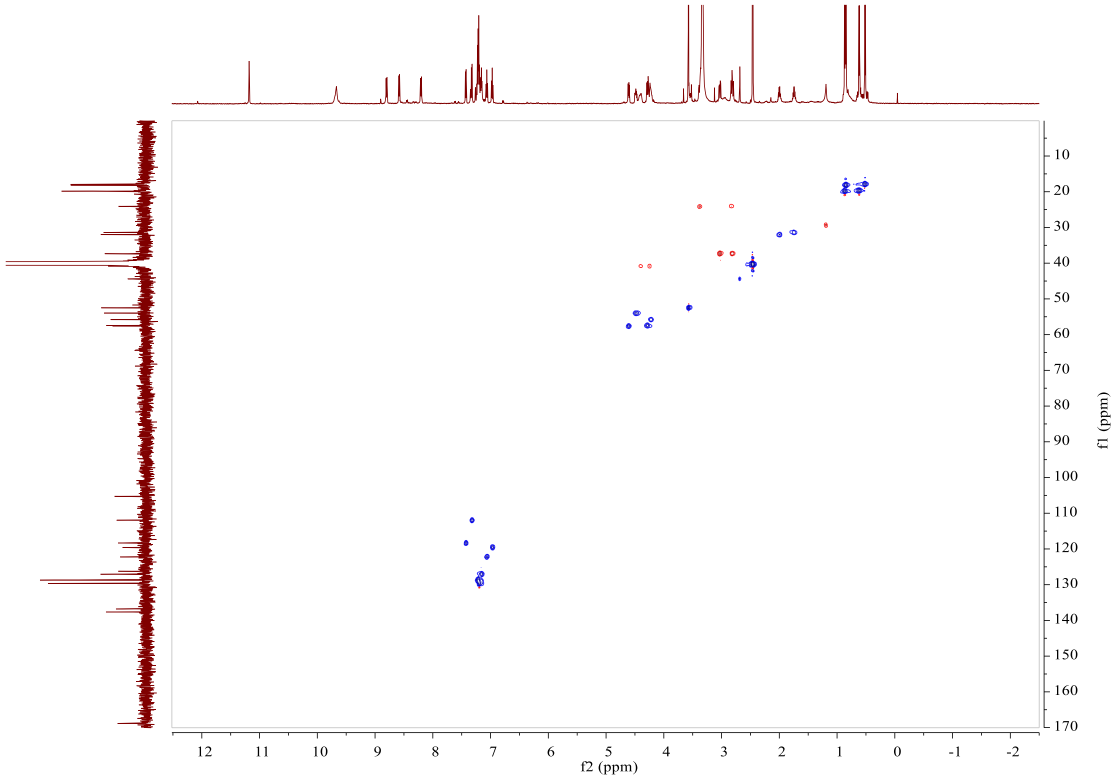

Figure S43. HSQC spectrum (600 MHz, DMSO-d_6) of compound **5**

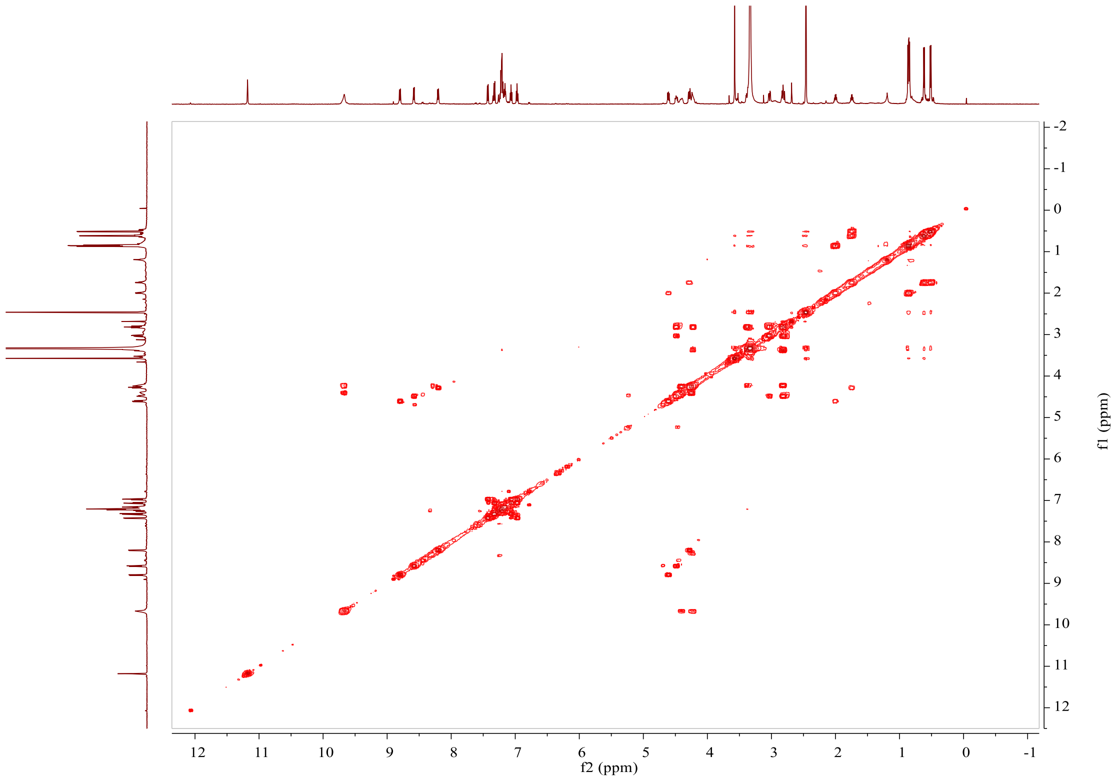

Figure S44. ^{1}H-^{1}H COSY spectrum (600 MHz, DMSO-d_6) of compound **5**

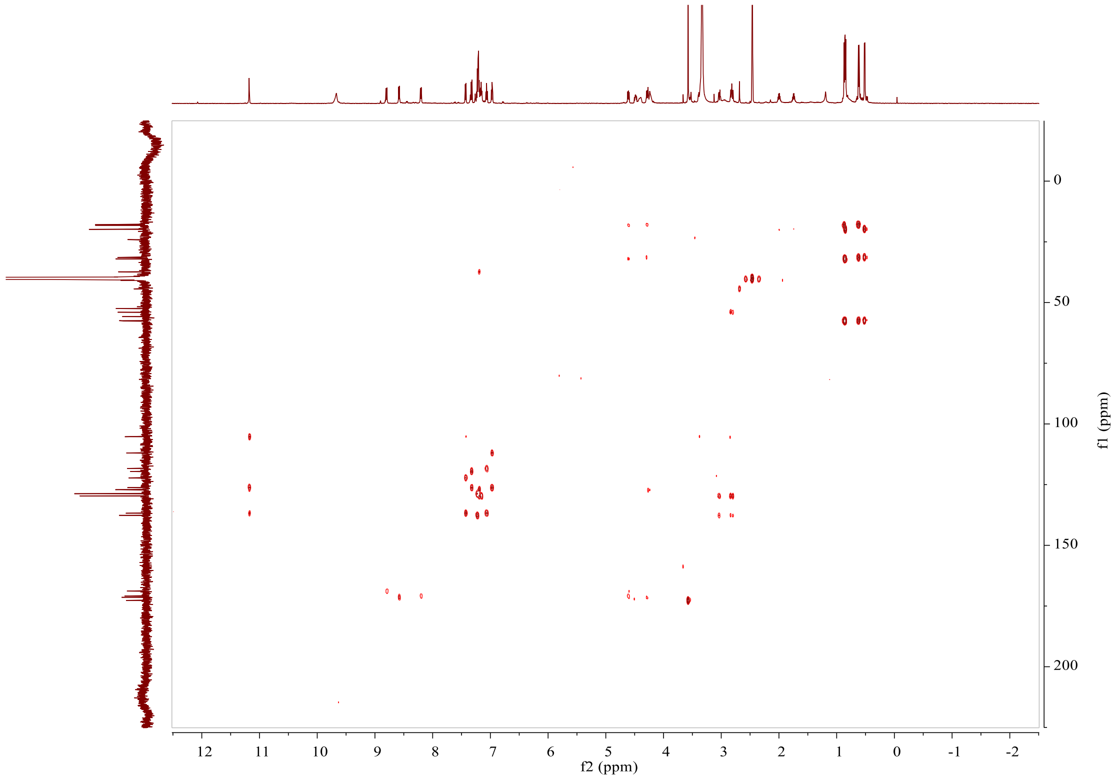

Figure S45. HMBC spectrum (600 MHz, DMSO-d_6) of compound **5**

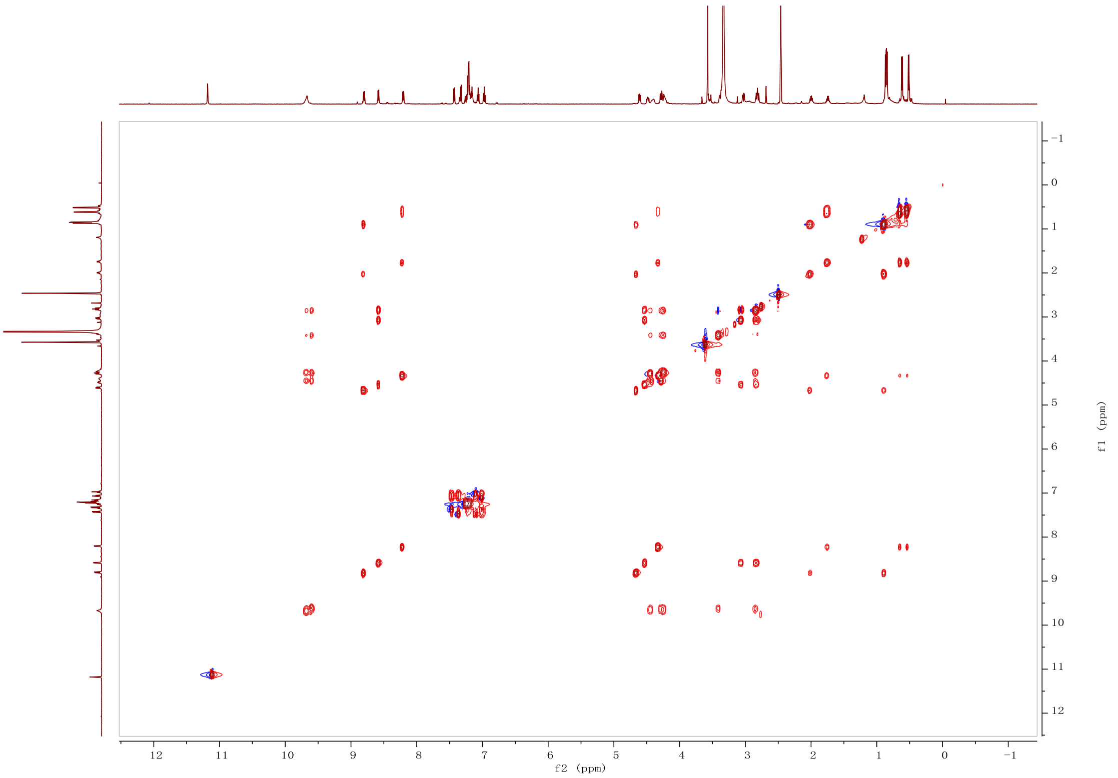

Figure S46. TOCSY spectrum (600 MHz, DMSO-d_6) of compound **5**

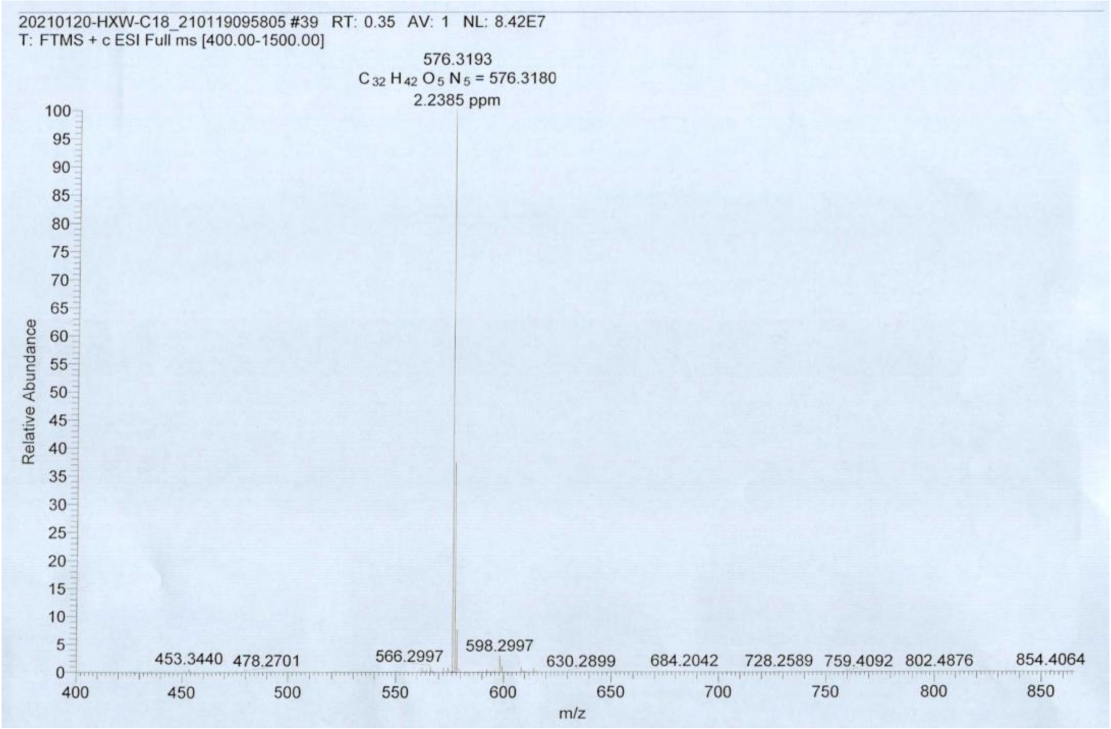

Figure S47. HRESIMS spectrum of compound **5**

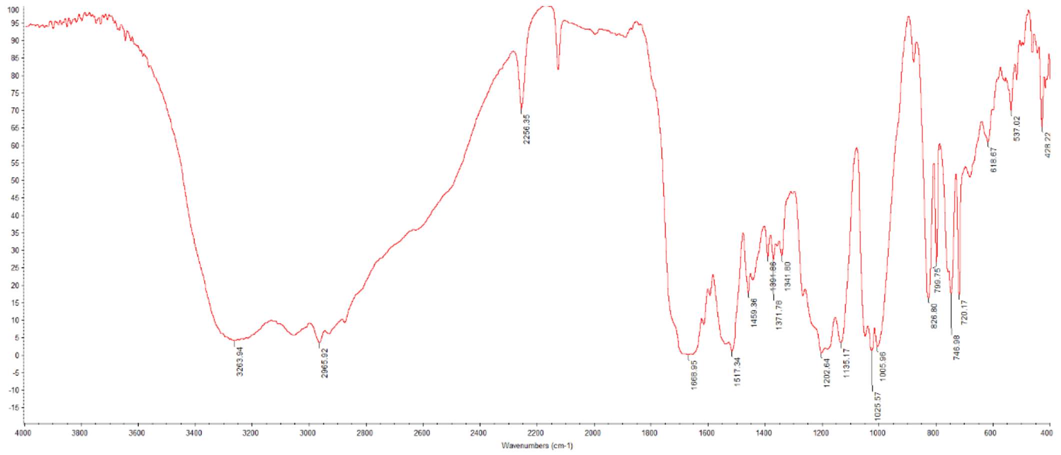

Figure S48. IR spectrum of compound **5**

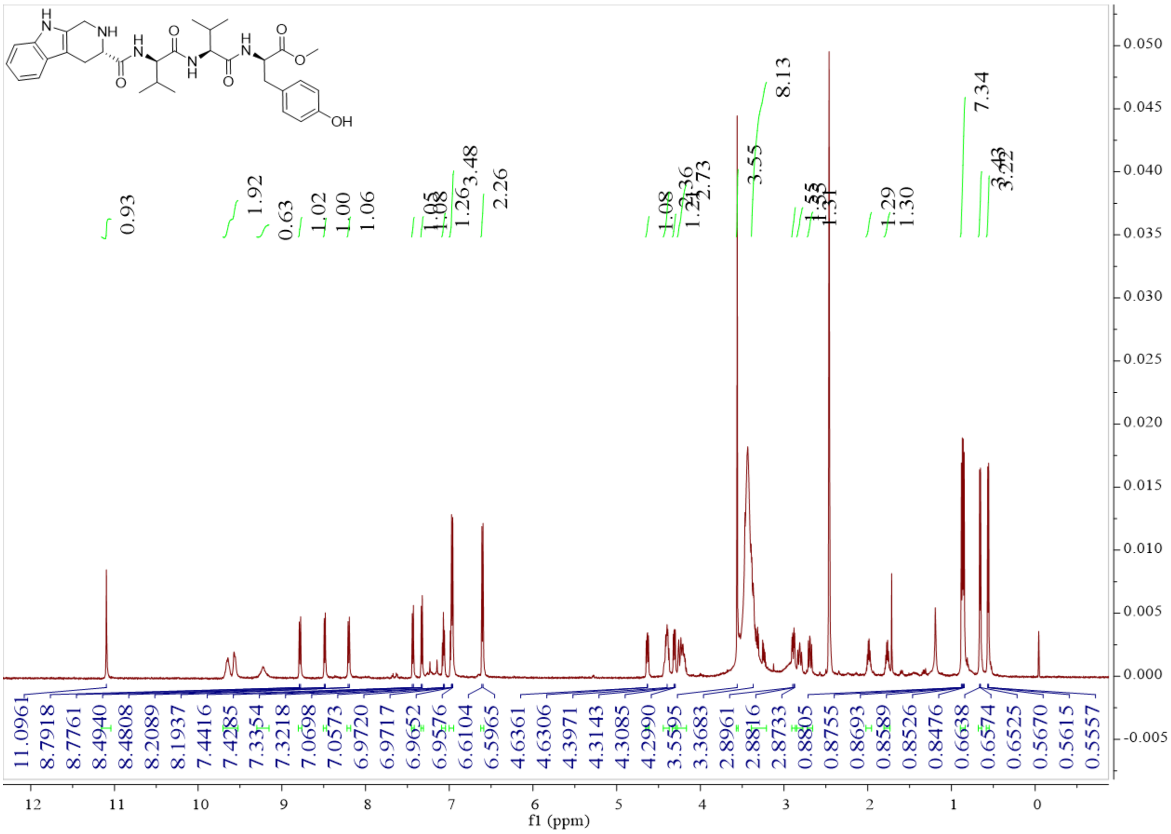

Figure S49. ^{1}H-NMR spectrum (600 MHz, DMSO-d_6) of compound **6**

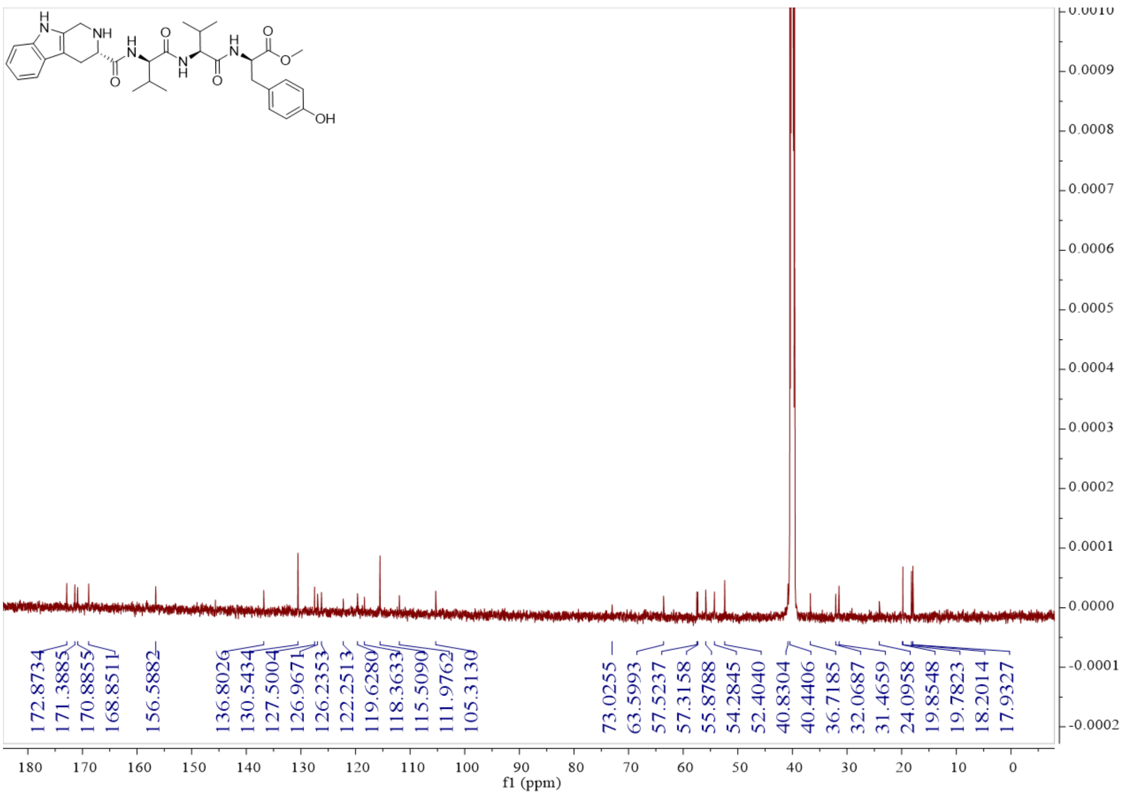

Figure S50. ^{13}C-NMR spectrum (150 MHz, DMSO-d_6) of compound **6**

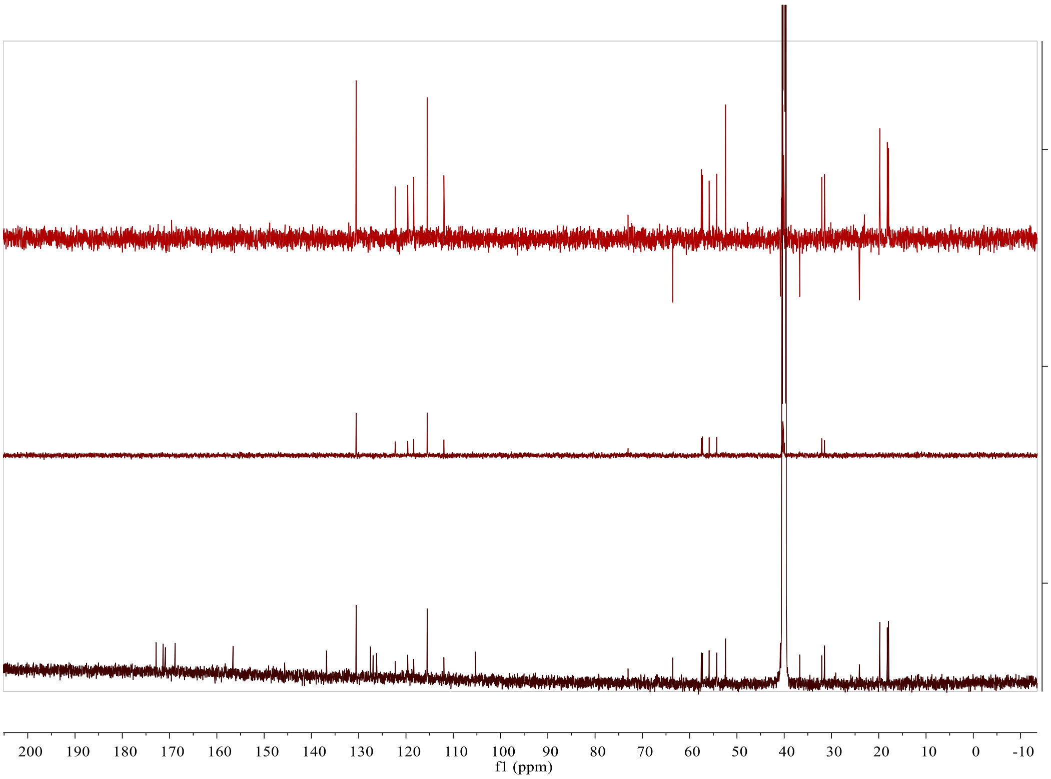

Figure S51. DEPT (150 MHz, DMSO-d_6) spectrum of compound **6**

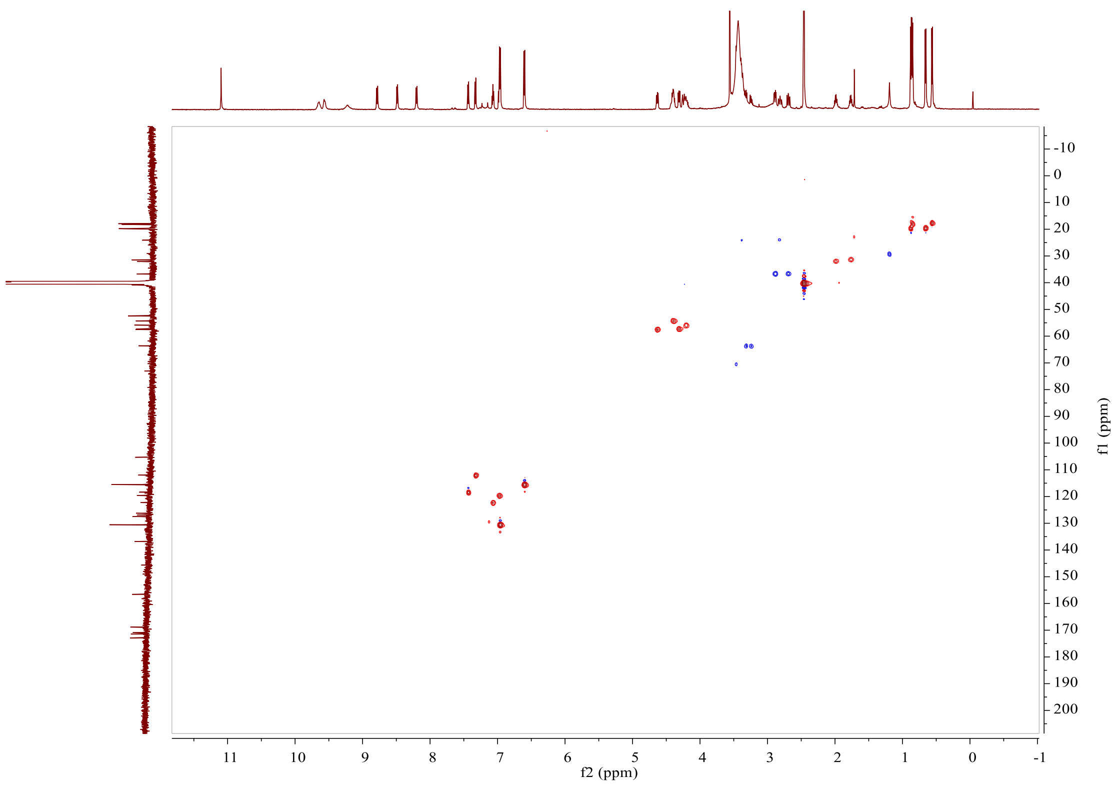

Figure S52. HSQC spectrum (600 MHz, DMSO-d_6) of compound **6**

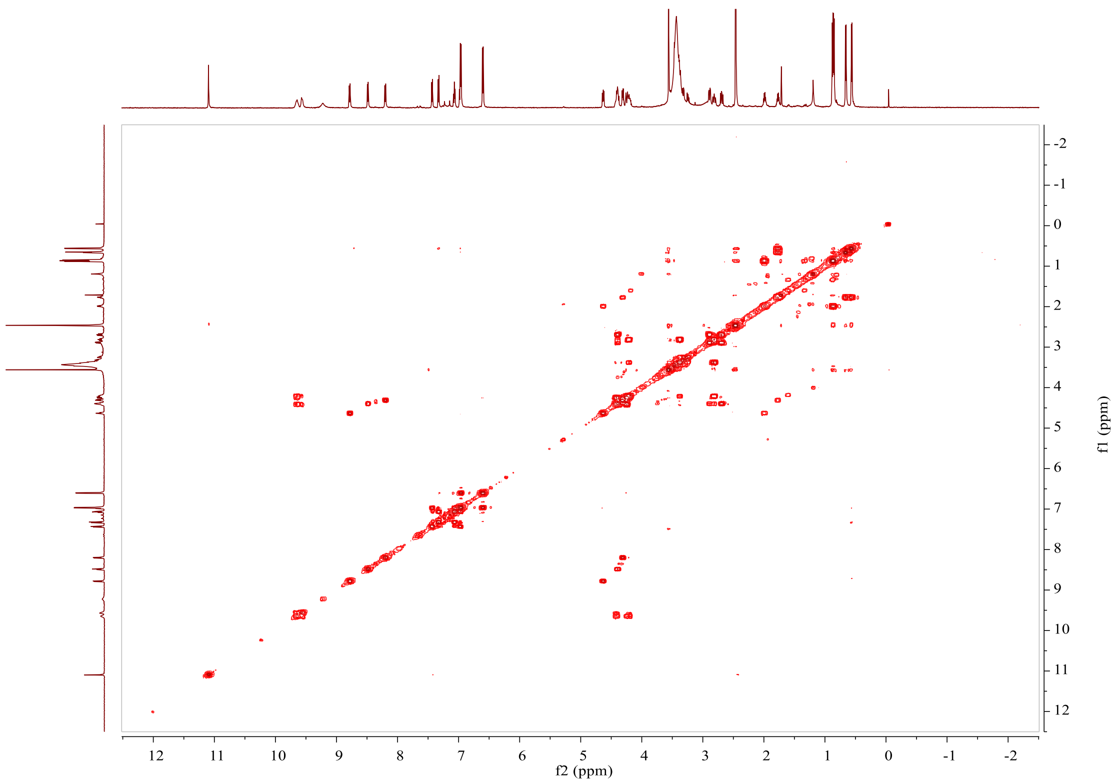

Figure S53. ^{1}H-^{1}H COSY spectrum (600 MHz, DMSO-d_6) of compound **6**

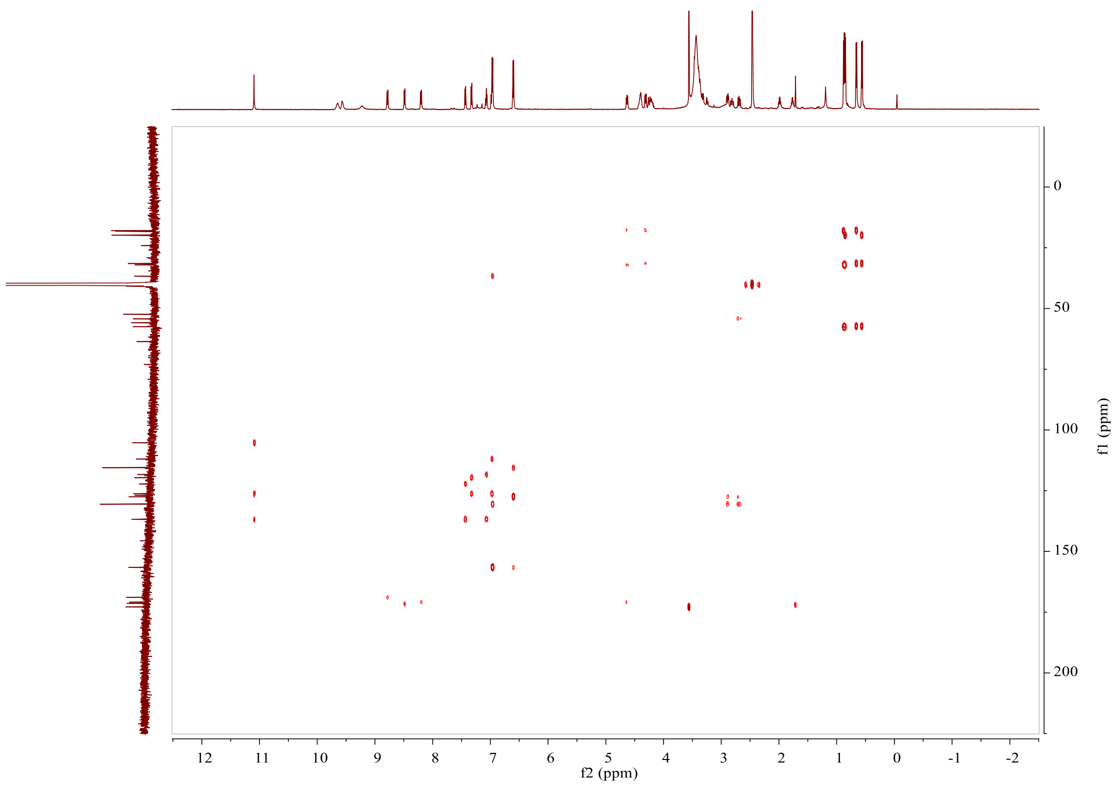

Figure S54. HMBC spectrum (600 MHz, DMSO-d_6) of compound **6**

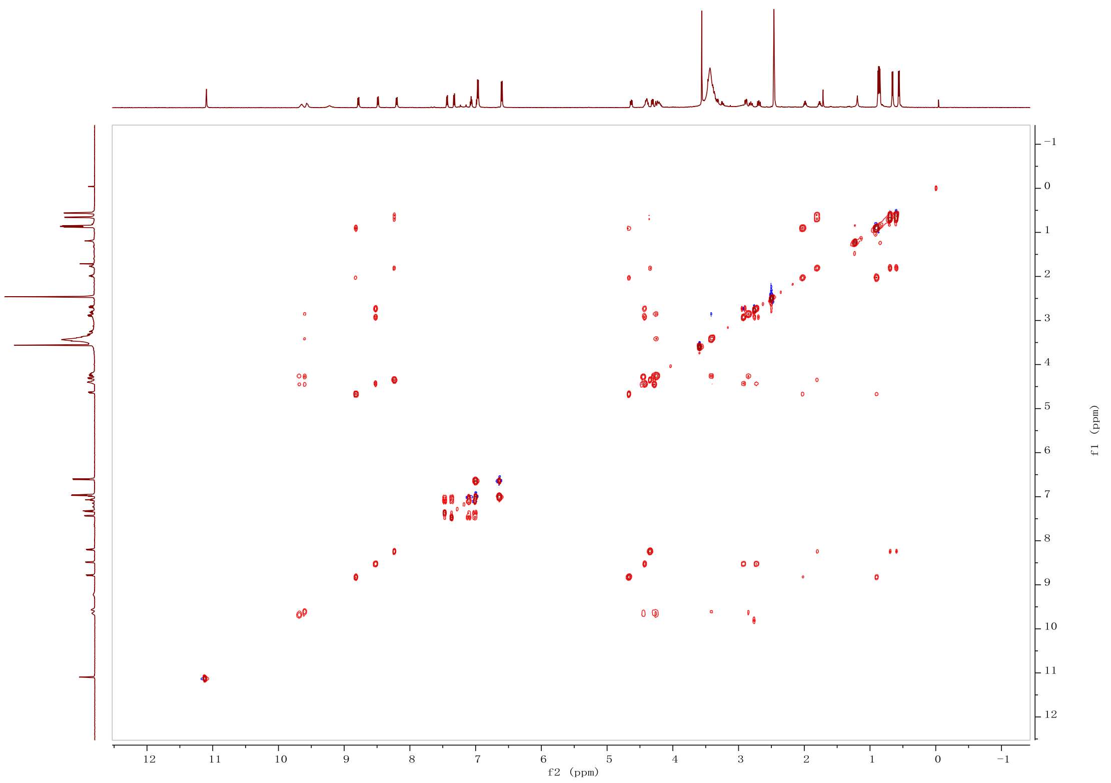

Figure S55. TOCSY spectrum (600 MHz, DMSO-d_6) of compound **6**

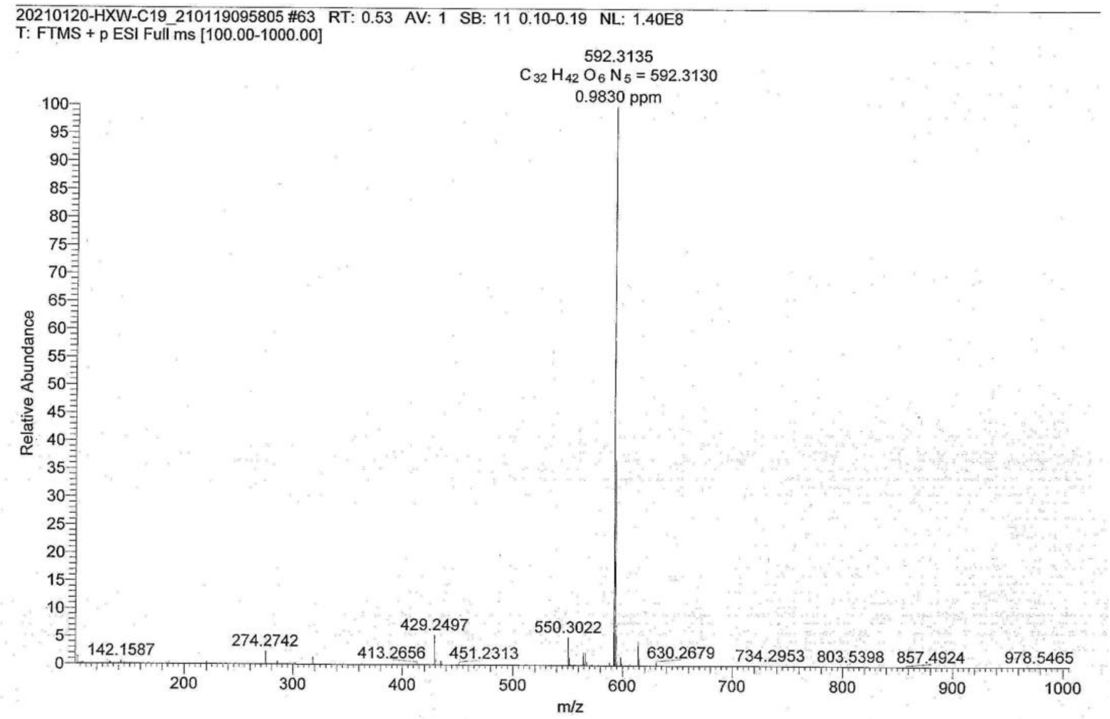

Figure S56. HRESIMS spectrum of compound **6**

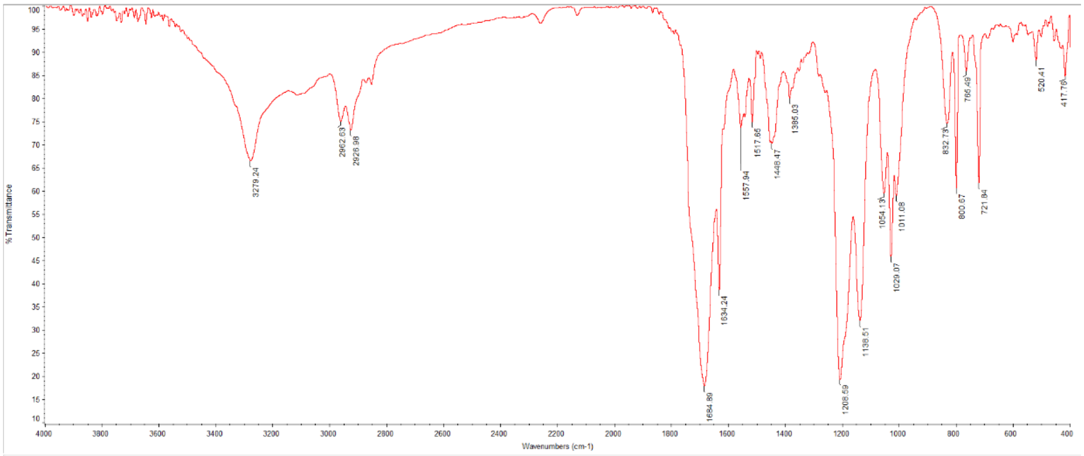

Figure S57. IR spectrum of compound **6**

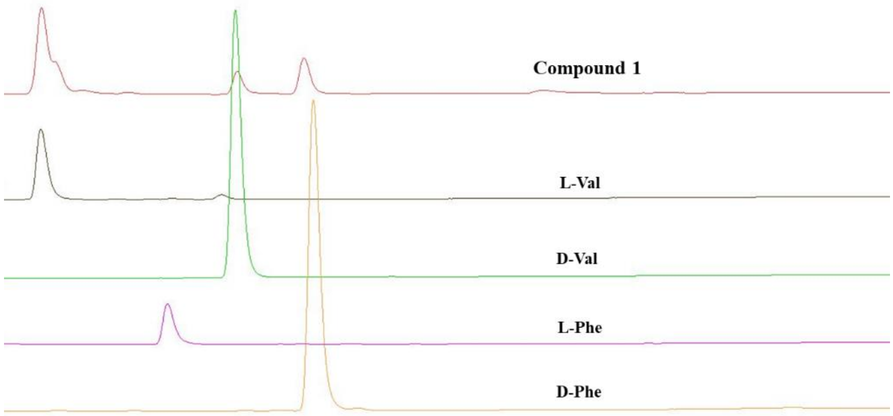

Figure S58. Advanced Marfey's acid hydrolytic analysis of compound **1**

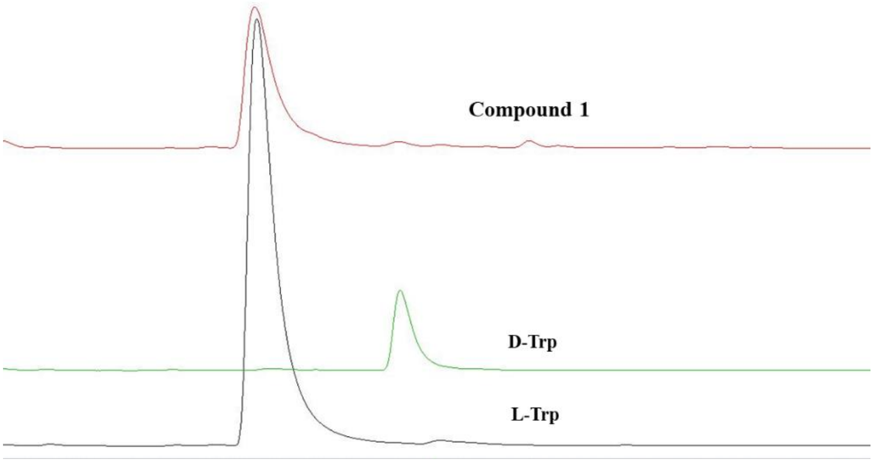

Figure S59. Advanced Marfey's alkaline hydrolytic analysis of compound **1**

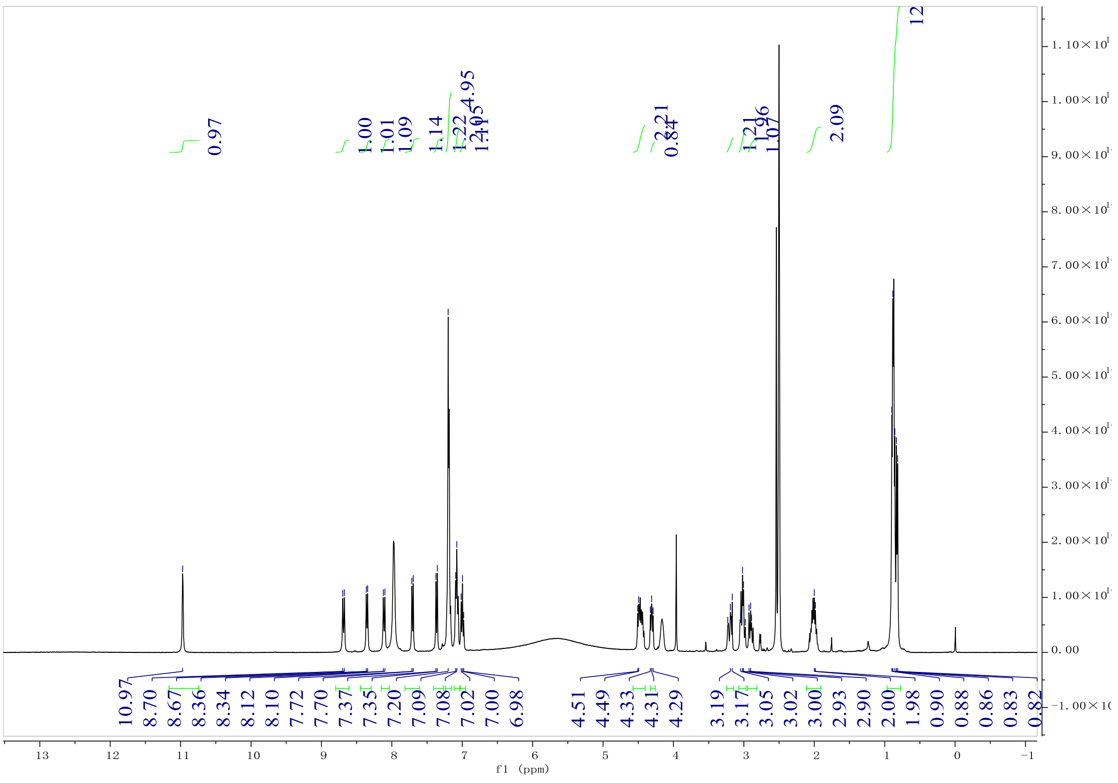

Figure S60. ^{1}H-NMR spectrum of **1a** (400 MHz, DMSO-d_6)

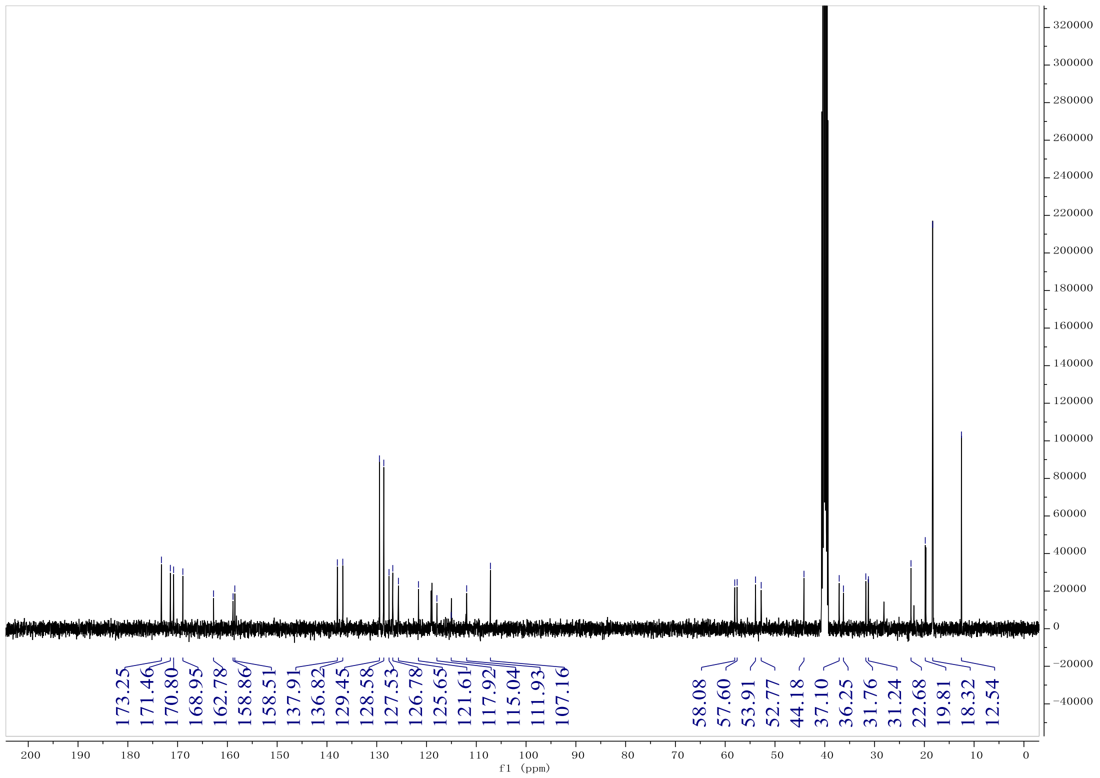

Figure S61. ^{13}C-NMR spectrum of **1a** (100 MHz, DMSO-d_6)

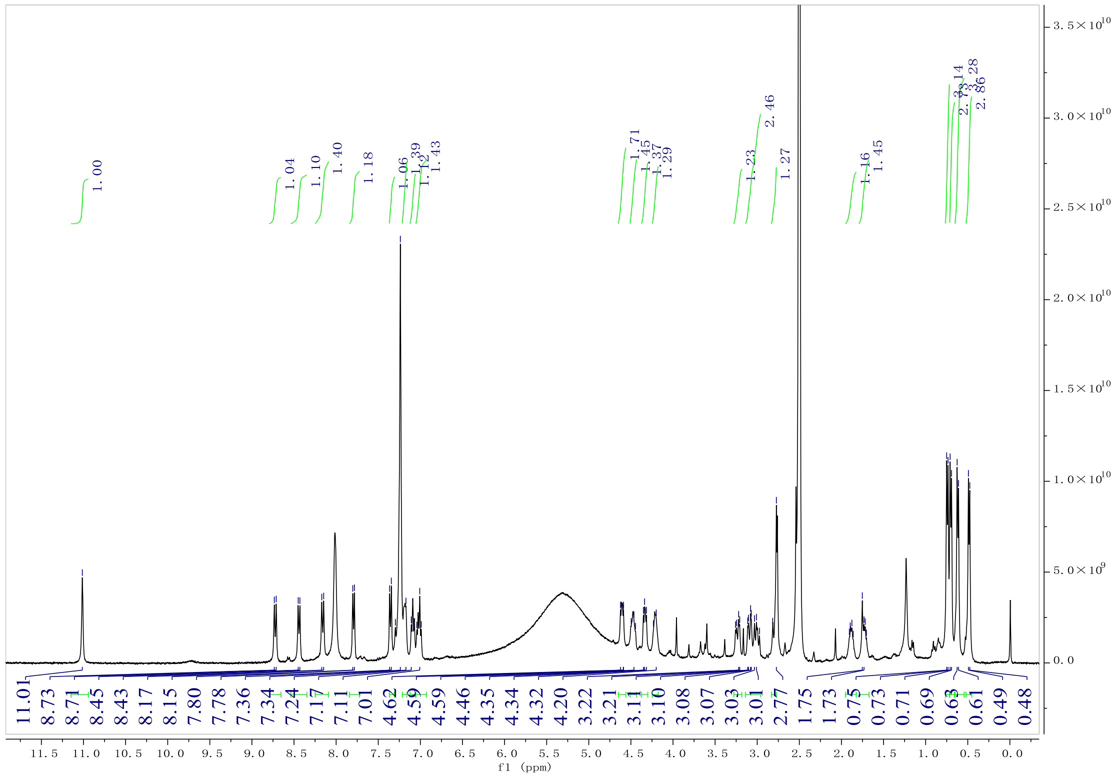

Figure S62. ^{1}H-NMR spectrum of **1b** (400 MHz, DMSO-d_6)

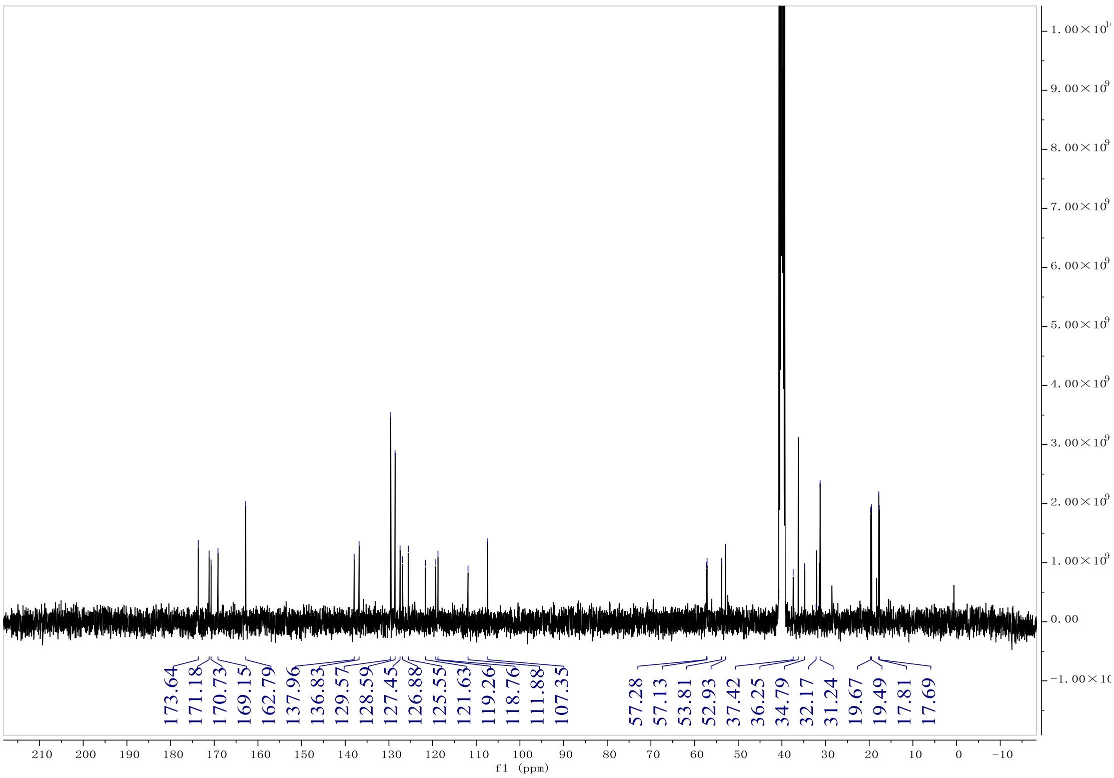

Figure S63. ^{13}C-NMR spectrum of **1b** (100 MHz, DMSO-d_6)

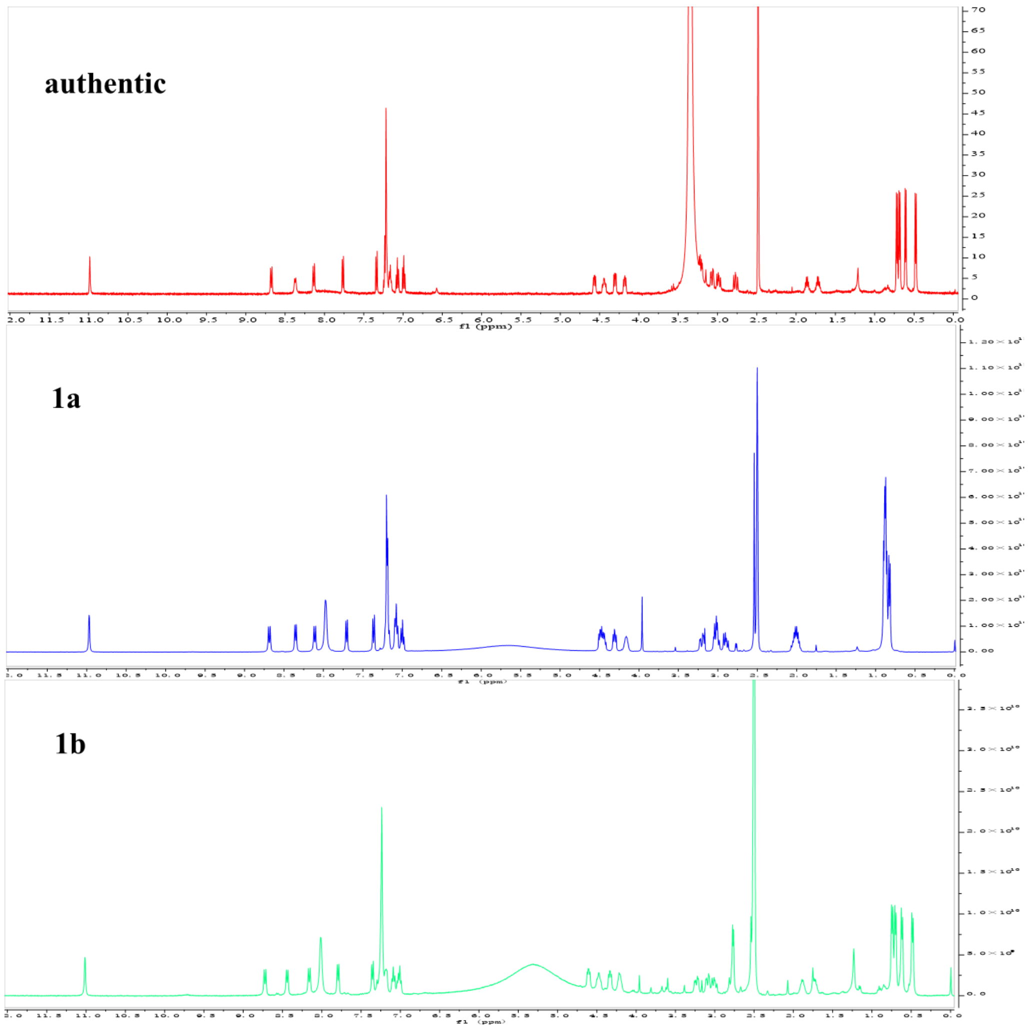

Figure S64. Comparison of ^{1}H-NMR spectra of authentic **1, 1a,** and **1b**

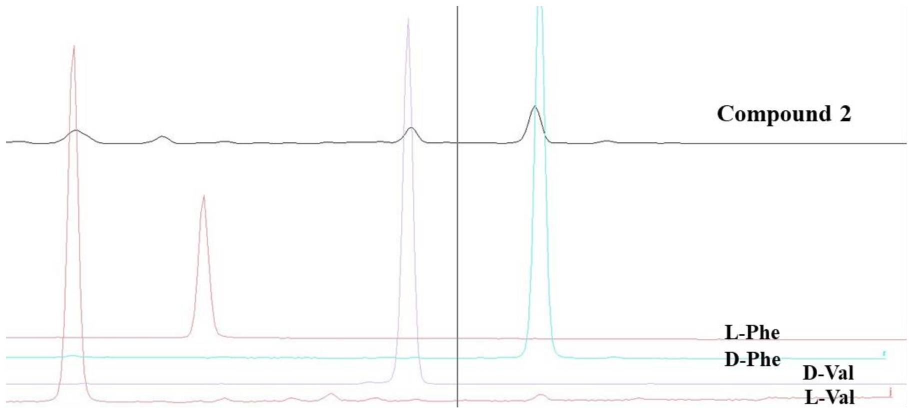

Figure S65. Advanced Marfey's acid hydrolytic analysis of compound **2**

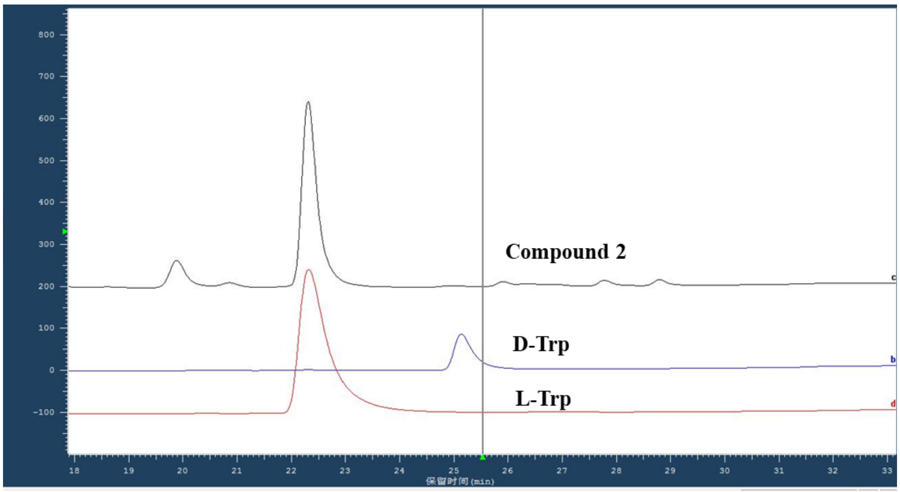

Figure S66. Advanced Marfey's alkaline hydrolytic analysis of compound **2**

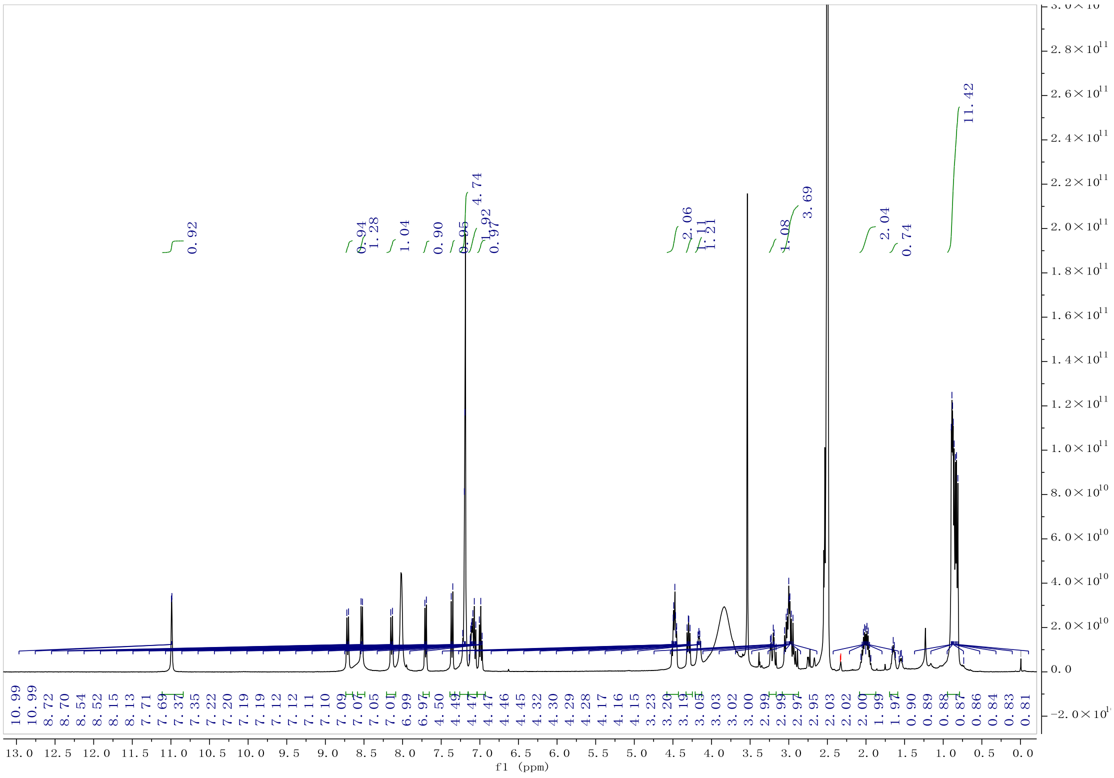

Figure S67. ^{1}H-NMR spectrum of **2a** (400 MHz, DMSO-d_6)

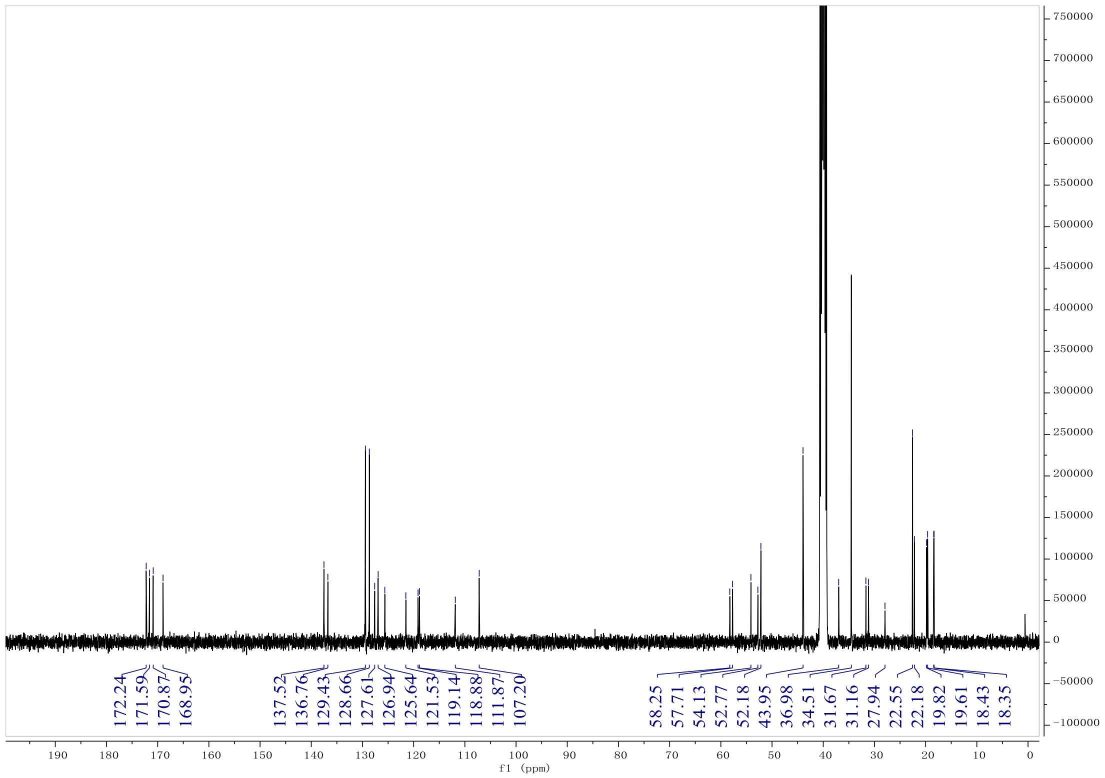

Figure S68. ^{13}C-NMR spectrum of **2a** (100 MHz, DMSO-d_6)

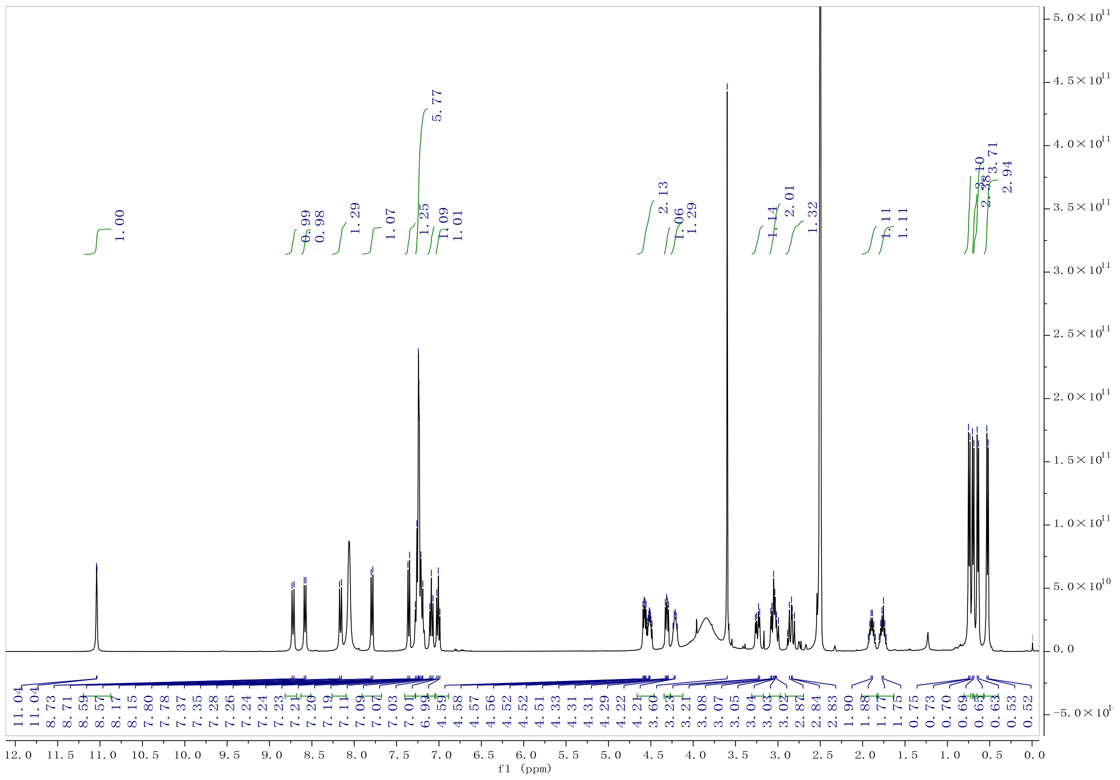

Figure S69. ^{1}H-NMR spectrum of **2b** (400 MHz, DMSO-d_6)

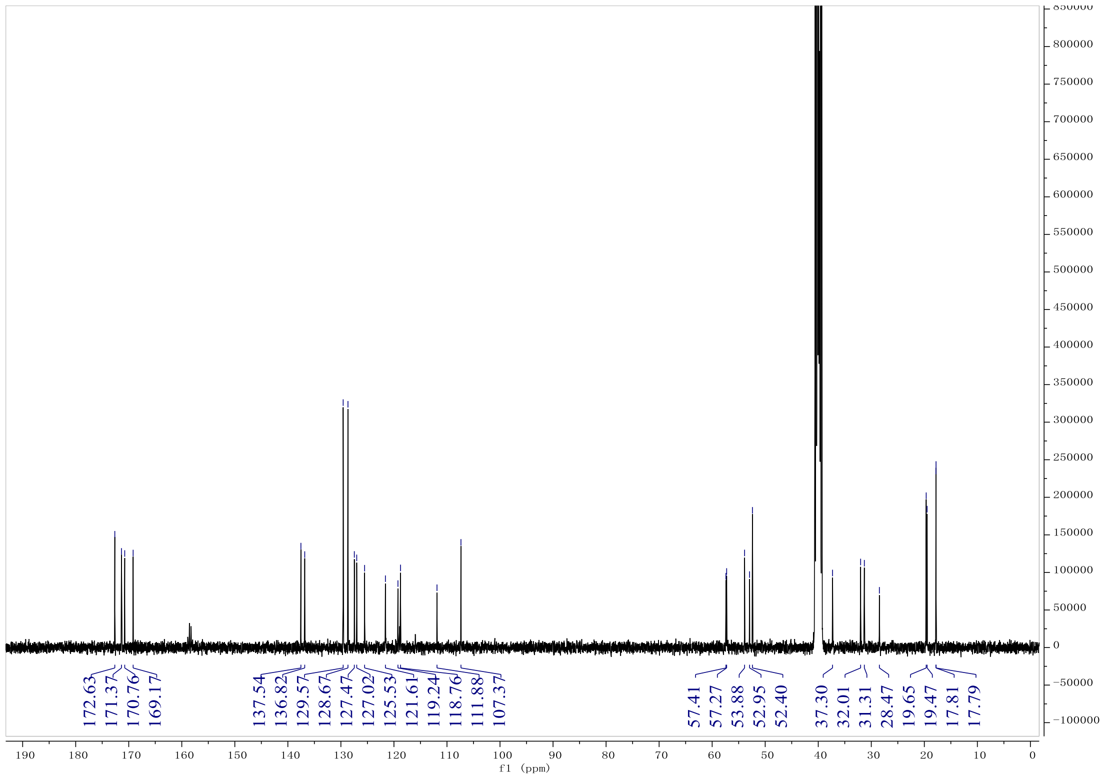

Figure S70. ^{13}C-NMR spectrum of **2b** (100 MHz, DMSO-d_6)

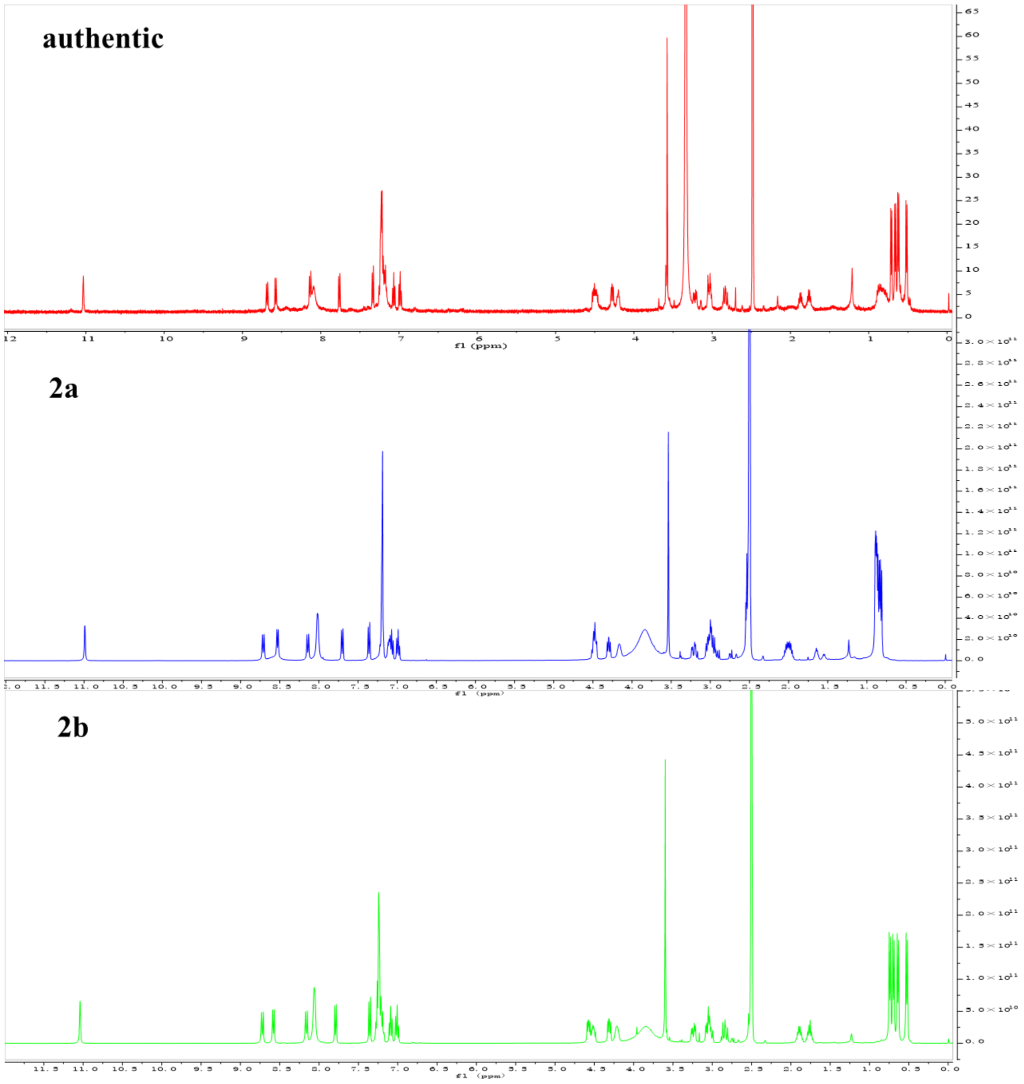

Figure S71. Comparison of ^{1}H-NMR spectra of authentic **2, 2a,** and **2b**

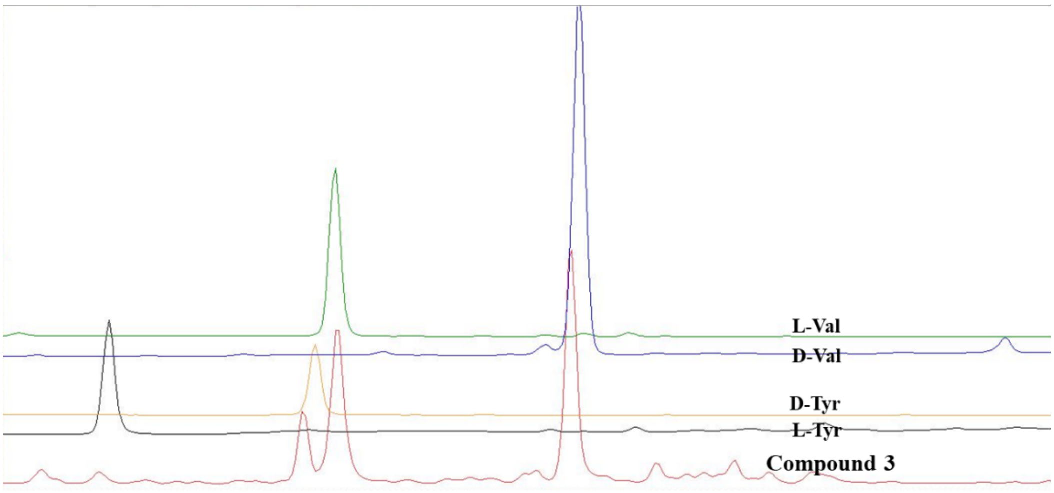

Figure S72. Advanced Marfey's acid hydrolytic analysis of compound **3**

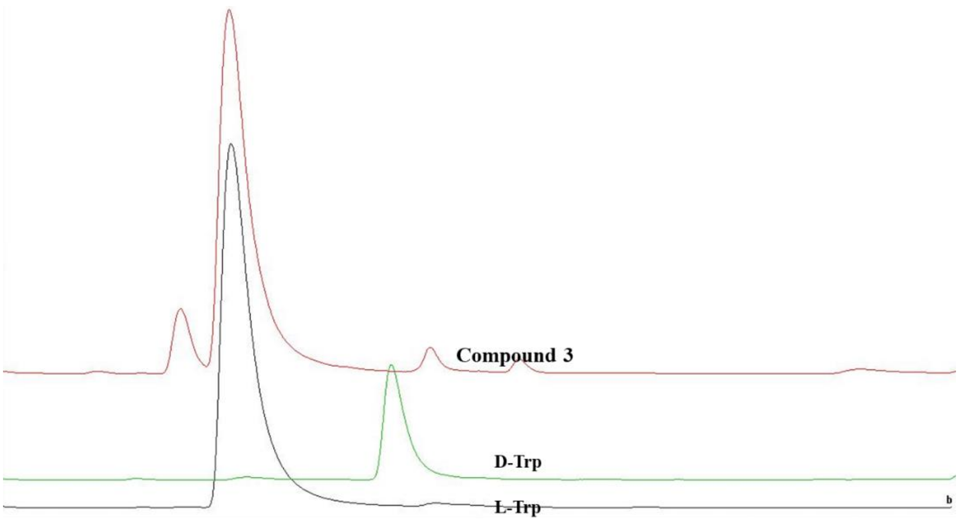

Figure S73. Advanced Marfey's alkaline hydrolytic analysis of compound **3**

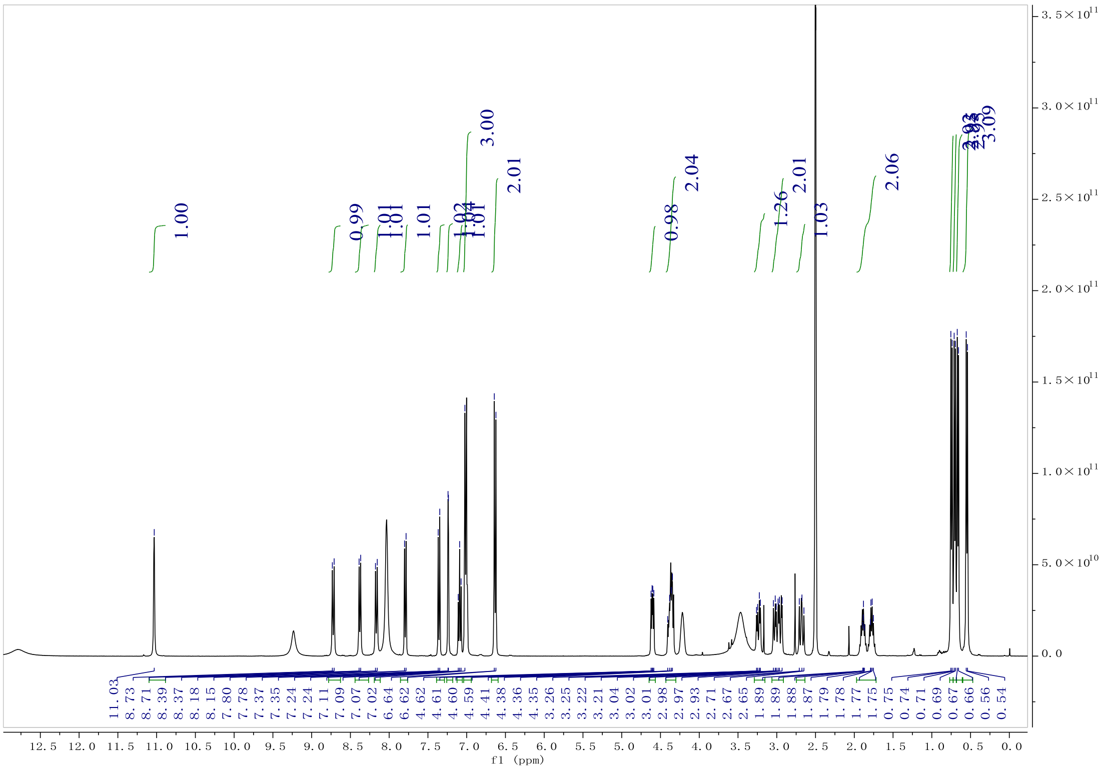

Figure S74. ^{1}H-NMR spectrum of synthetic **3** (400 MHz, DMSO-d_6)

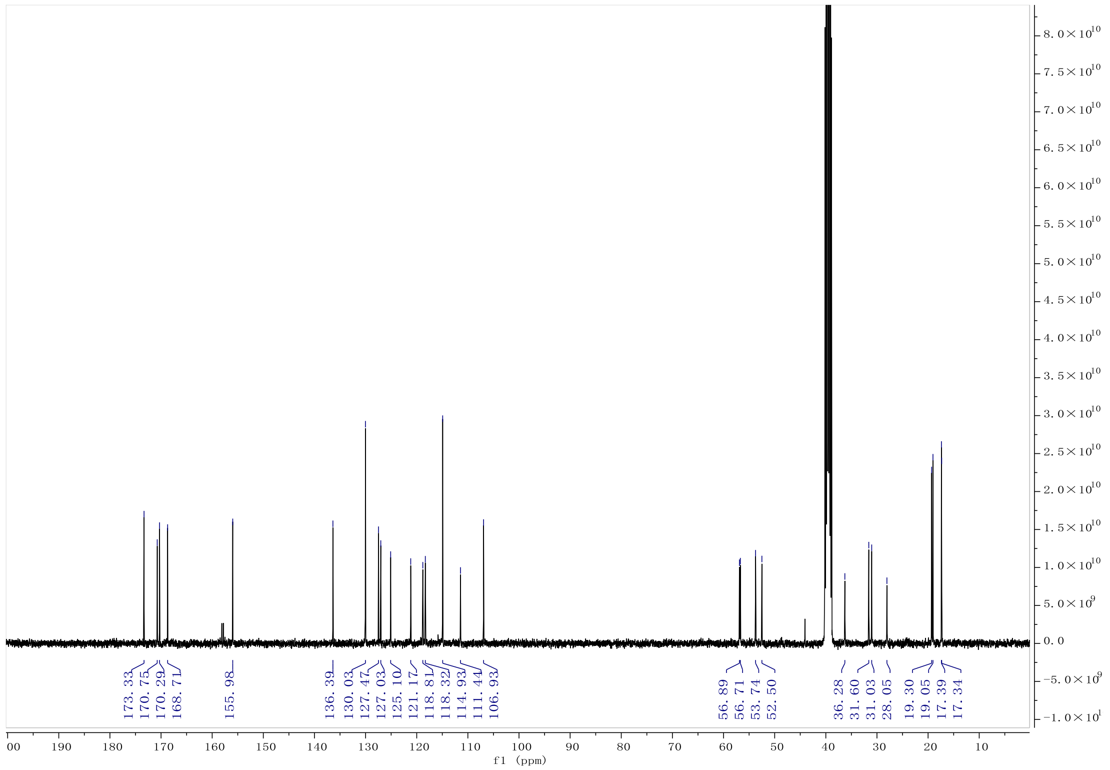

Figure S75. ^{13}C-NMR spectrum of synthetic **3** (100 MHz, DMSO-d_6)

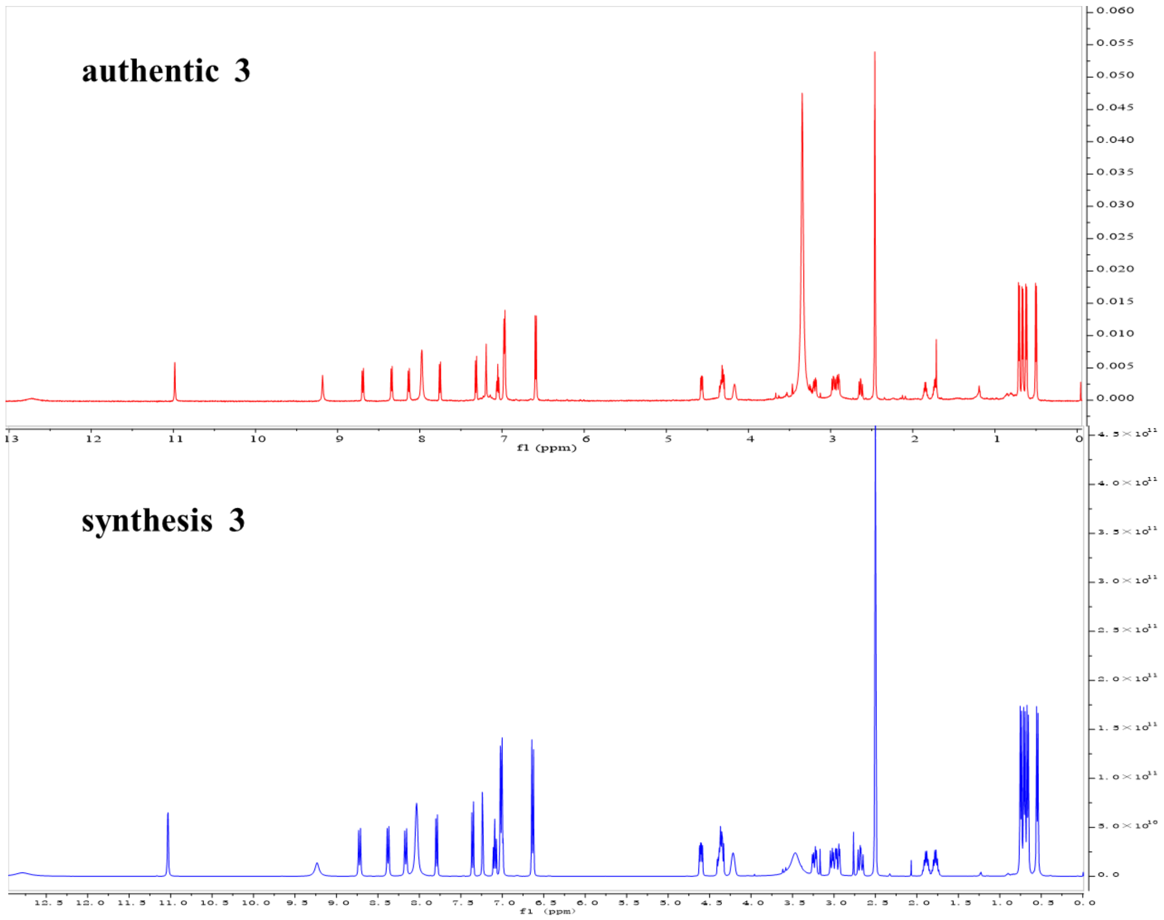

Figure S76. Comparison of ^{1}H-NMR spectra of **3** with synthetic **3**

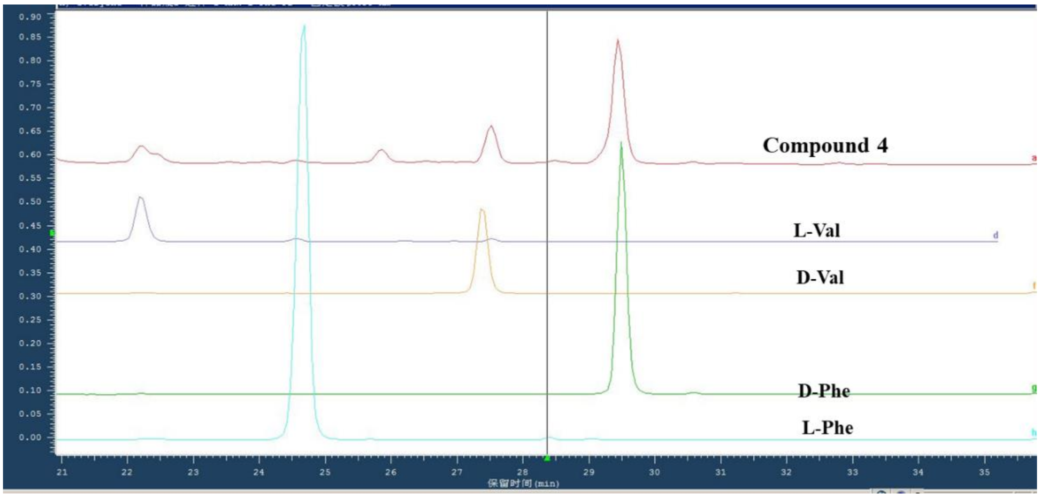

Figure S77. Advanced Marfey's acid hydrolytic analysis of compound **4**

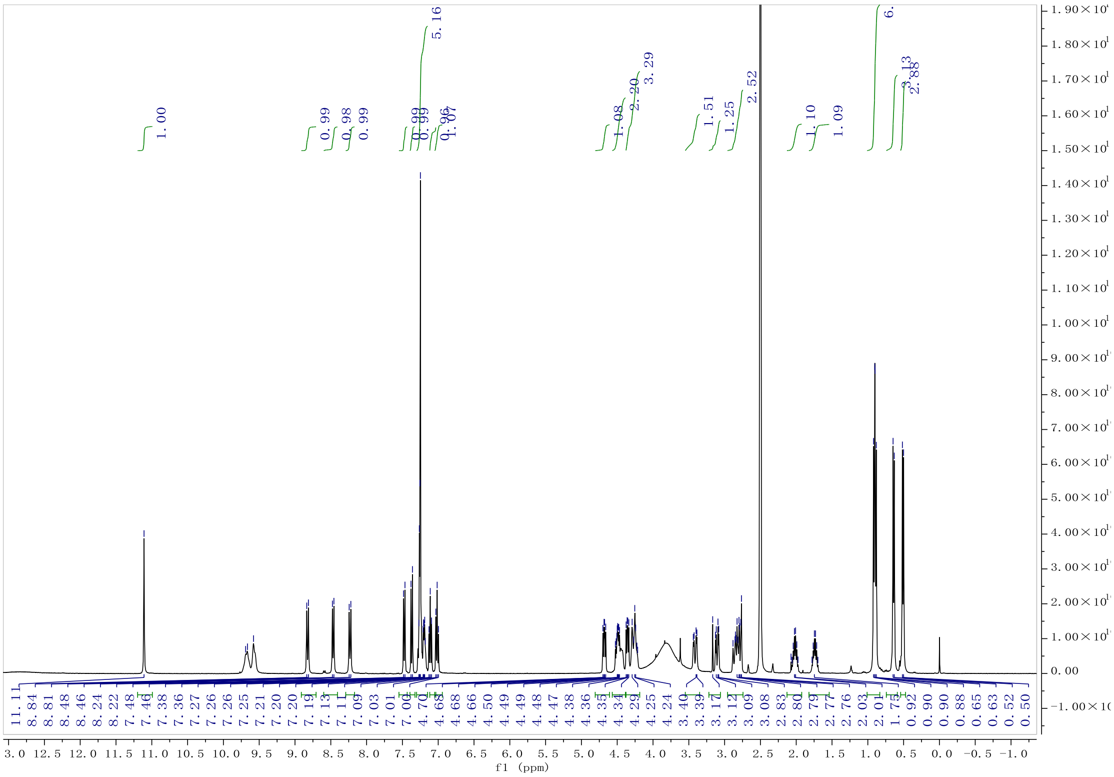

Figure S78. ^{1}H-NMR spectrum of synthetic **4** (400 MHz, DMSO-d_6)

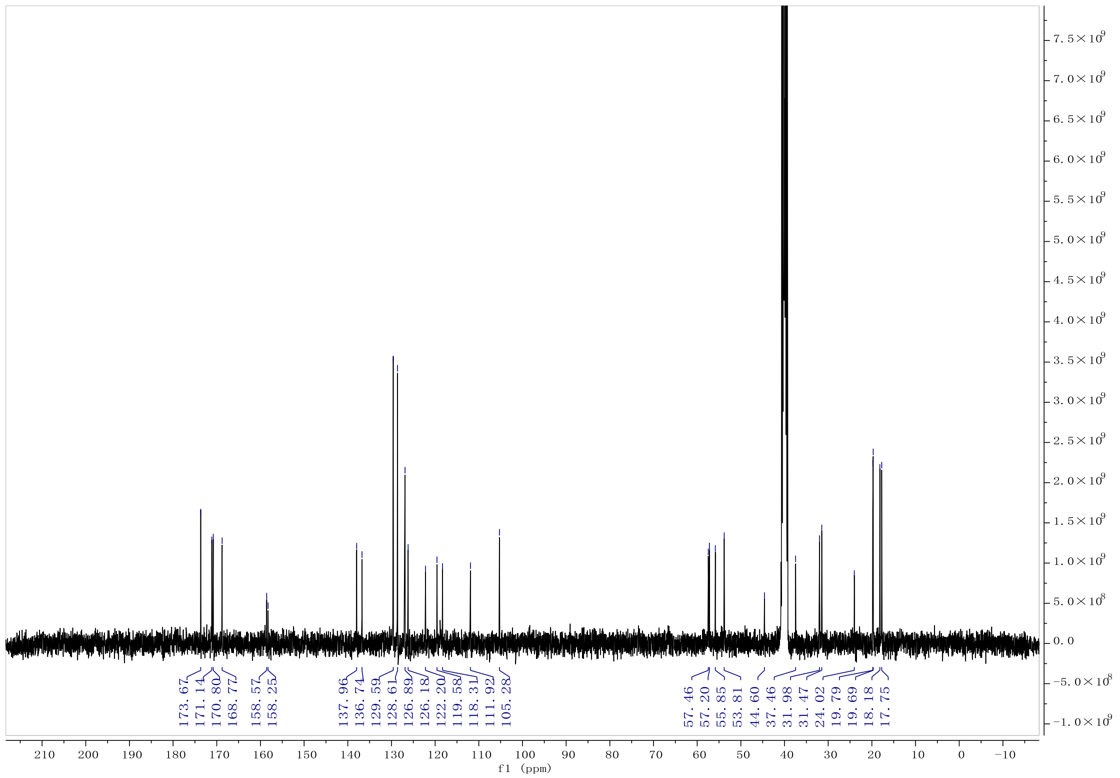

Figure S79. ^{13}C-NMR spectrum of synthetic **4** (100 MHz, DMSO-d_6)

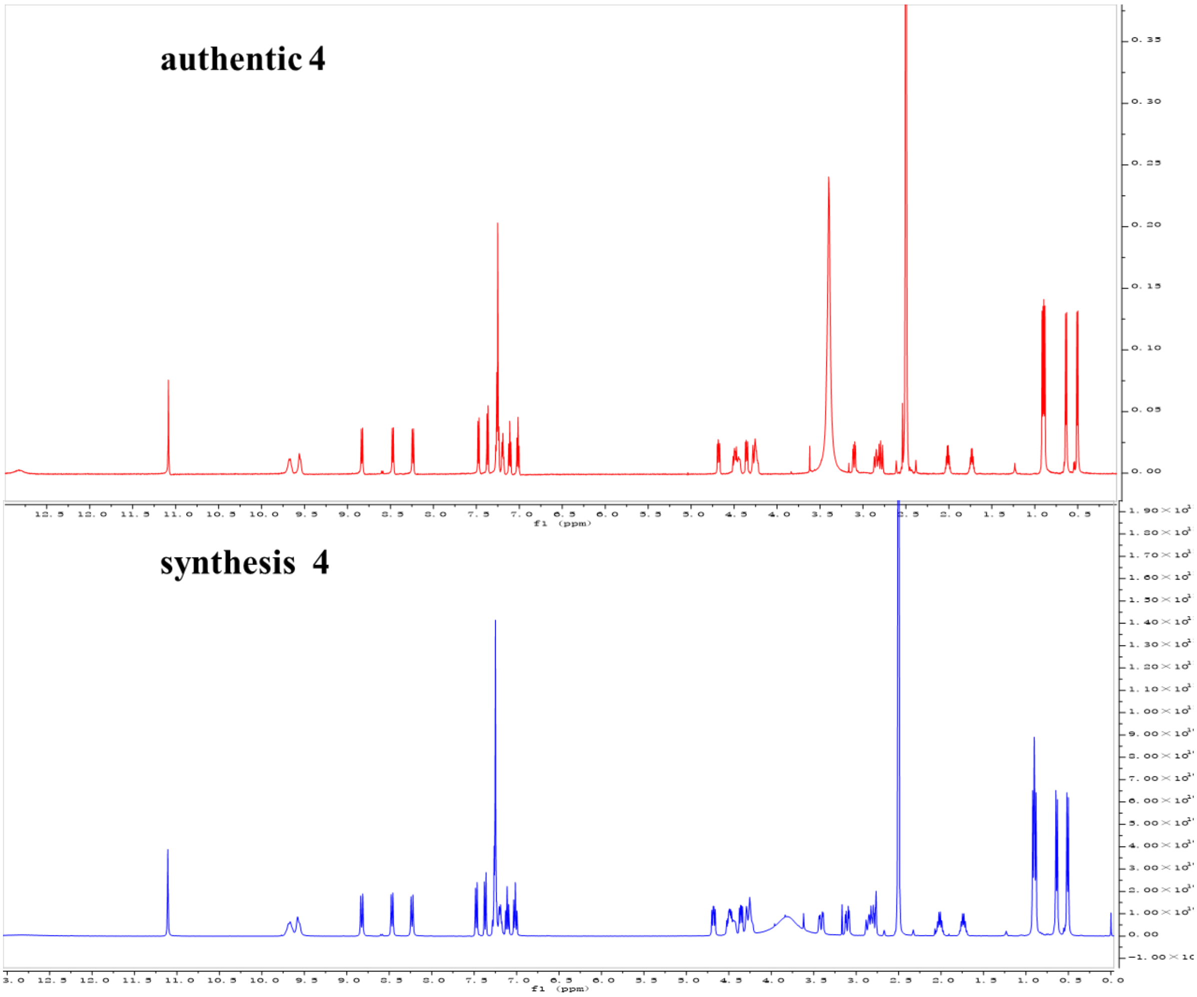

Figure S80. Comparison of ^{1}H-NMR spectra of **4,** with synthestic **4**

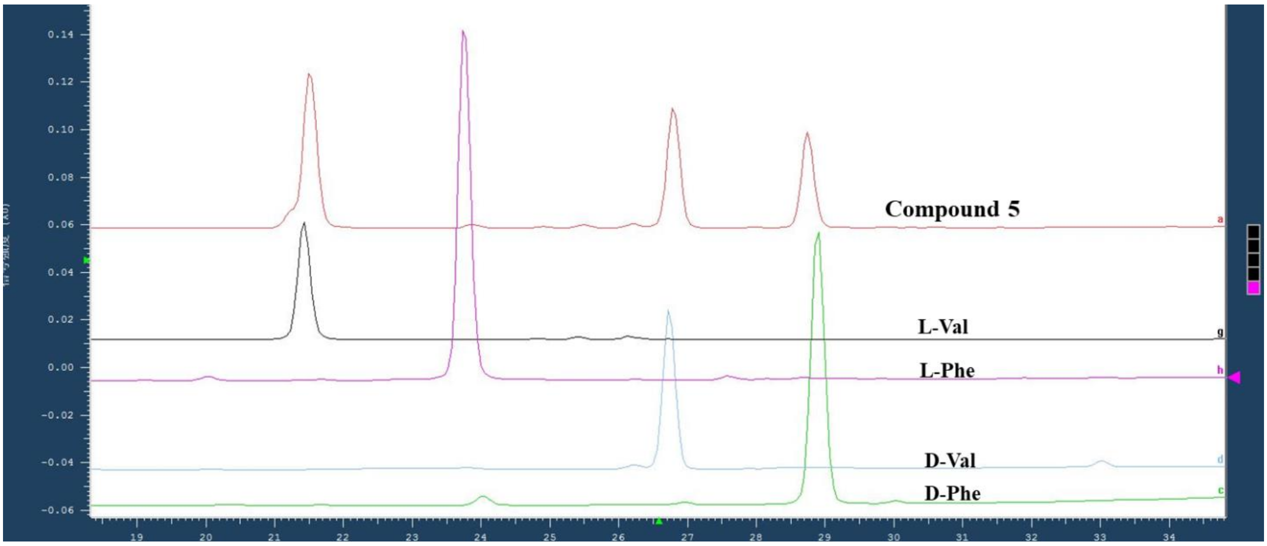

Figure S81. Advanced Marfey's acid hydrolytic analysis of compound **5**

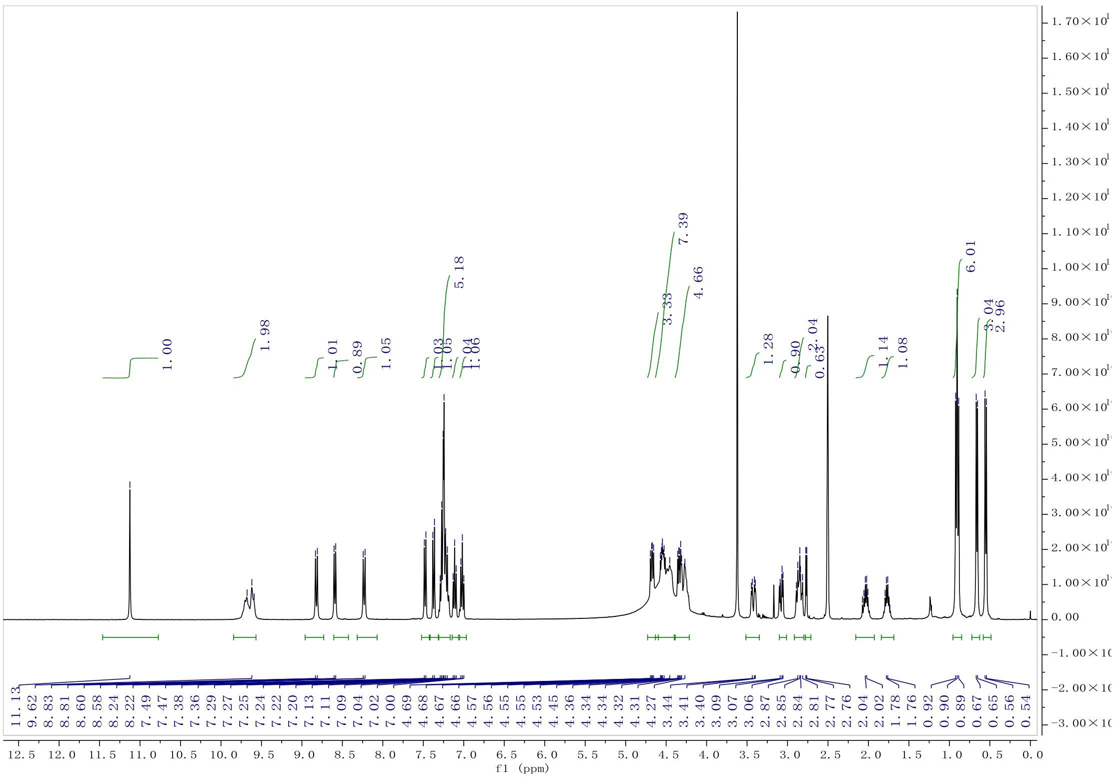

Figure S82. ^{1}H-NMR spectrum of synthetic **5** (400 MHz, DMSO-d_6)

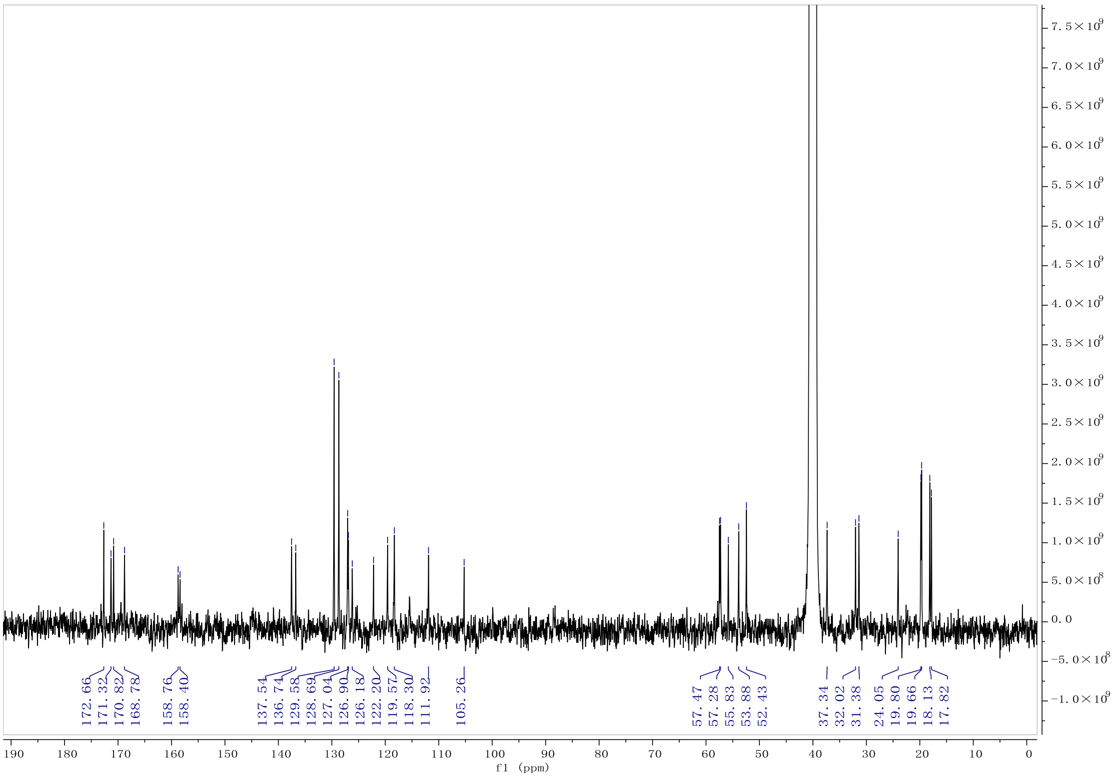

Figure S83. ^{13}C-NMR spectrum of synthetic **5** (100 MHz, DMSO-d_6)

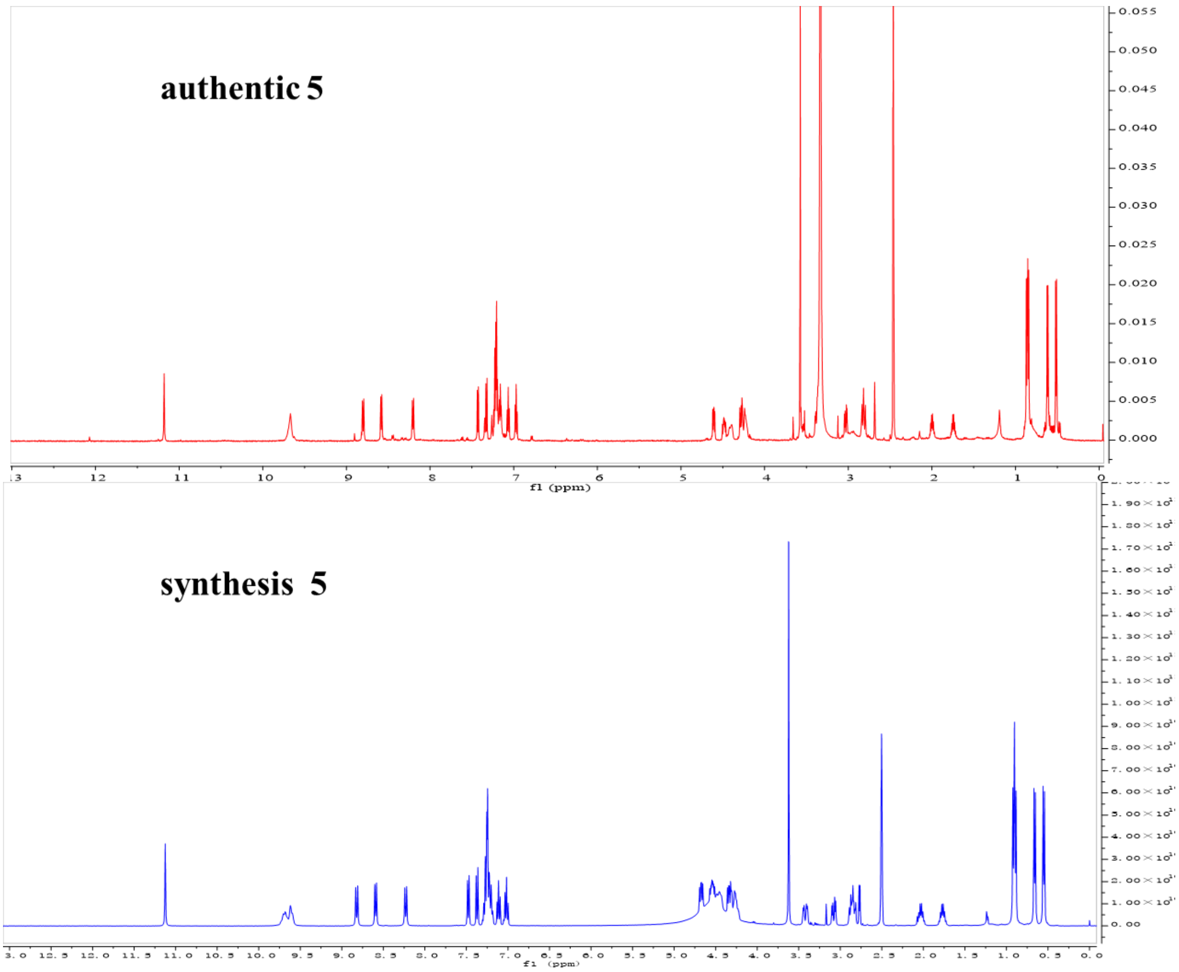

Figure S84. Comparison of ^{1}H-NMR spectra of **5** with synthetic **5**

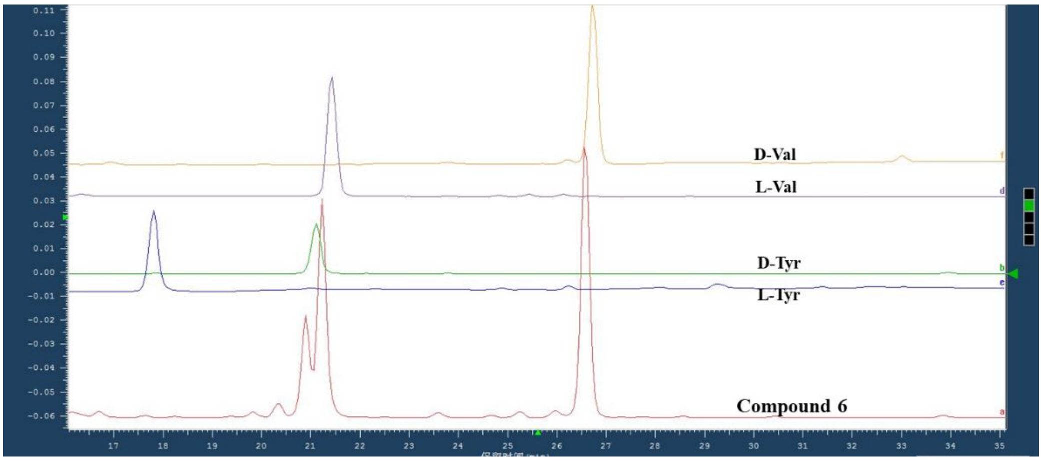

Figure S85. Advanced Marfey's acid hydrolytic analysis of compound **6**

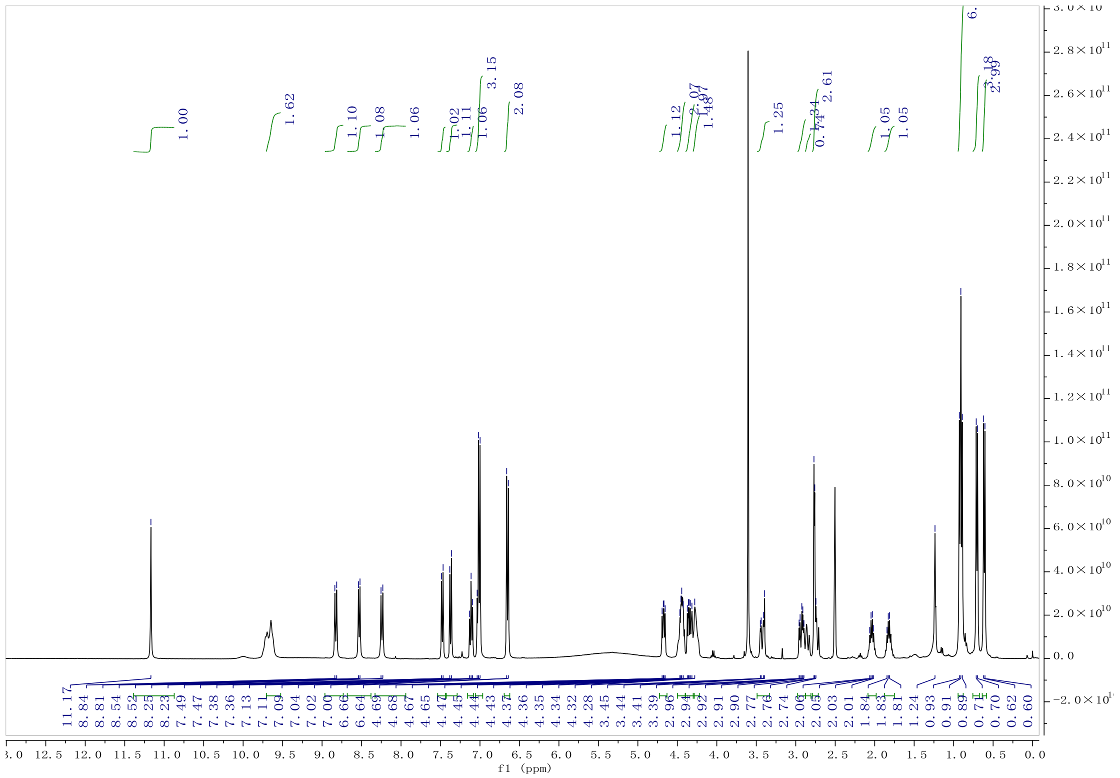

Figure S86. ^{1}H-NMR spectrum of synthetic **6** (400 MHz, DMSO-d_6)

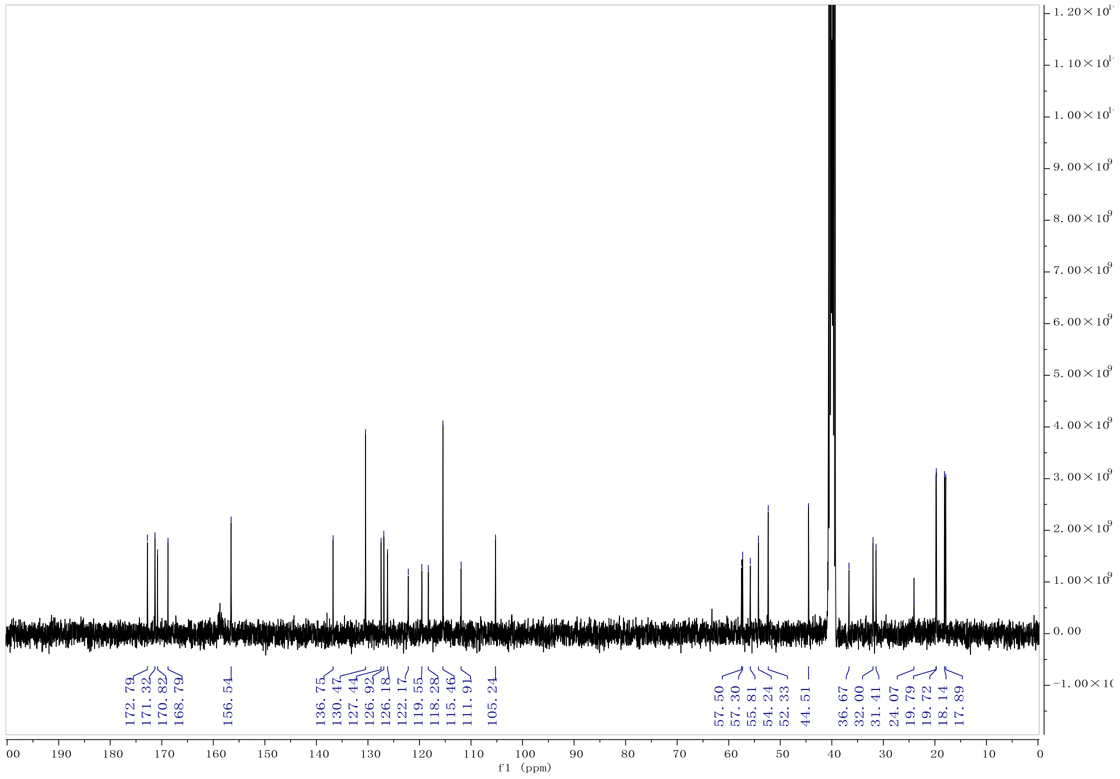

Figure S87. ^{13}C-NMR spectrum of synthetic **6** (100 MHz, DMSO-d_6)

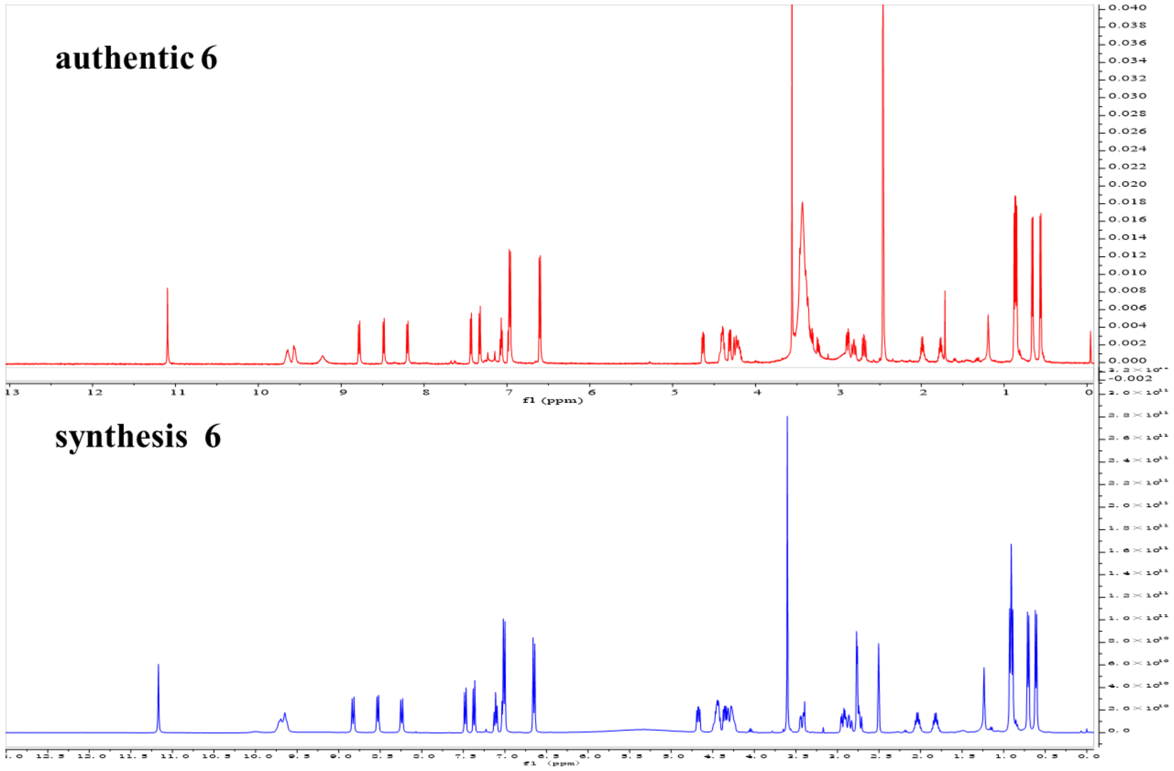

Figure S88. Comparison of ^{1}H-NMR spectra of **6** with synthetic **6**

Table S1. Table of compounds (**1-6**) (100 μM) inhibition of ACh-evoked peak current amplitude mediated by human (h) $\alpha1\beta1\varepsilon\delta$, $\alpha1\beta1\gamma\delta$, $\alpha3\beta2$, $\alpha3\beta4$, $\alpha4\beta2$, $\alpha7$ and $\alpha9\alpha10$ nAChRs. Whole-cell currents at h$\alpha1\beta1\varepsilon\delta$ and h$\alpha1\beta1\gamma\delta$ were activated by 5 μM ACh, h$\alpha3\beta2$ and h$\alpha9\alpha10$ were activated by 6 μM ACh, and h$\alpha3\beta4$, h$\alpha4\beta2$, and h$\alpha7$ were activated by 300, 3, and 100 μM ACh, respectively (mean ± SD, n = 6 – 11).

Compounds	Relative current amplitude						
	(h) α1β1εδ	(h)α1β1γδ	(h)α3β2	(h)α3β4	(h)α4β2	(h)α7	(h)α9α10
**1**	0.190 ± 0.056	0.664 ± 0.062	0.435 ± 0.071	0.462 ± 0.069	0.637± 0.053	0.293 ± 0.074	0.103 ± 0.027
**2**	0.024 ± 0.011	0.017 ± 0.014	0.236 ± 0.069	0.071 ± 0.028	0.301 ± 0.039	0.566 ± 0.072	0.075 ± 0.036
**3**	0.648 ± 0.067	0.861 ± 0.084	0.451 ± 0.056	0.675 ± 0.040	0.793 ± 0.078	0.368 ± 0.088	0.509 ± 0.041
**4**	0.757 ± 0.065	0.985 ± 0.019	0.924 ± 0.069	0.670 ± 0.049	0.972 ± 0.057	0.228 ± 0.049	0.824 ± 0.064
**5**	0.392 ± 0.069	0.799 ± 0.063	0.656 ± 0.074	0.656 ± 0.051	0.837 ± 0.061	0.399 ± 0.061	0.374 ± 0.073
**6**	0.618 ± 0.047	0.802 ± 0.096	0.579 ± 0.077	0.777 ± 0.053	0.938 ± 0.076	0.838 ± 0.066	0.760 ± 0.062

Table S2. Free binding energy estimation and molecular interactions of compounds **1-6**

ligand	free bindingenergy (kcal/mol)	molecular interaction
compound **1**	-4.0	Trp[1]: H-donor with PHE106(B)
		Phe[4] : H-acceptor with SER84(B)
compound **2**	-4.2	Trp[1]: H-donor with SER180(A) and ARG48(B); pi-H with GLN50(B)
		Val[3]: H-donor with ASP49(A)
		Phe[4]-OCH_3:H-acceptor with ARG48(B)
compound **3**	-3.9	Trp[1]: H-donor with ALA108(B)
		Val[2]: H-acceptor with LYS85(B)
compound **4**	-2.8	Val[3]: H-acceptor with ASN109(B)
		Phe[4]: pi-H with ASP96(A)
compound **5**	-3.1	Val[3]:H-pi with PHE144(A)
		Phe[4]-OCH_3: pi-H with ARG214(A)
compound **6**	-3.4	Val[3]: H-pi with PHE144(A)
